# Crebanine mitigates glucocorticoid‐induced osteonecrosis of the femoral head by restoring bone remodelling homeostasis via attenuating oxidative stress

**DOI:** 10.1111/jcmm.70044

**Published:** 2024-08-28

**Authors:** Shankun Dong, Jianxun Ge, Qi Meng, Tao Yuan, Yi Wang, Yi Li, Qizhen Lu, Wenao Song, Ziqing Li, Shui Sun

**Affiliations:** ^1^ Department of Joint Surgery Shandong Provincial Hospital, Cheeloo College of Medicine, Shandong University Jinan Shandong China; ^2^ Department of Joint Surgery Shandong Provincial Hospital Affiliated to Shandong First Medical University Jinan Shandong China; ^3^ Orthopaedic Research Laboratory, Medical Science and Technology Innovation Center Shandong First Medical University and Shandong Academy of Medical Sciences Jinan Shandong China; ^4^ Department of Clinical Laboratory Shandong Provincial Hospital, Cheeloo College of Medicine, Shandong University Jinan Shandong China

**Keywords:** crebanine, network pharmacology, Nrf2, ONFH, osteoblast, osteoclast, ROS

## Abstract

The onset of osteonecrosis of the femoral head (ONFH) is intimately associated with the extensive administration of glucocorticoids (GCs). Long‐term stimulation of GCs can induce oxidative stress in both osteoclasts (OCs) and osteoblasts (OBs), resulting in the disturbance of bone remodelling. An alkaloid named crebanine (CN) demonstrates pharmacological properties including anti‐inflammation and reactive oxygen species (ROS) modulation. Our objective is to assess the therapeutic potential of CN in treating ONFH and elucidate the associated underlying mechanisms. The network pharmacology analysis uncovered that CN played a role in regulating ROS metabolism. In vitro, CN demonstrated its ability to reduce the dexamethasone (DEX)‐stimulated generation of OCs and suppress their resorptive function by downregulating the level of osteoclast marker genes. Concurrently, CN also mitigated DEX‐induced damage to OBs, facilitating the restoration of osteoblast marker gene expression, cellular differentiation and function. These effects were achieved by CN augmenting the antioxidant system to reduce intracellular ROS levels. Furthermore, in vitro results were corroborated by micro‐CT and histological data, which also showed that CN attenuated MPS‐induced ONFH in mice. This study highlights the therapeutic potential of CN in counteracting GCs‐induced ONFH.

## 1 INTRODUCTION

Osteonecrosis of the femoral head (ONFH) is an ischaemic disorder affecting the femoral head, characterized by severe pain, progressive devastation of the femoral head and dysfunction of the hip joint. The pathogenesis of ONFH is uncertain, with primary risk factors entail prolonged or high‐dose glucocorticoids (GCs) treatment and excessive alcohol consumption.^1^ To date, treatments for ONFH include medications such as vasodilators and bisphosphonates, as well as surgical interventions such as core decompression and total hip arthroplasty, among others.^1^, ^2^, ^3^ While surgical therapies prove effective for late‐stage ONFH, they are not suitable for early‐stage treatment. Likewise, the medications meant to retard the disease progression have proven ineffective and can cause adverse effects.^4^, ^5^ Therefore, the search for safer, more effective medications based on the understanding of pathogenesis to prevent or decelerate the progression of ONFH continues.

Earlier studies demonstrated that GCs contribute to increased levels of reactive oxygen species (ROS) in both osteoblasts (OBs) and osteoclasts (OCs).^6^ ROS impedes the differentiation of OBs while promoting osteoclastogenesis, results in augmented bone loss.^7^, ^8^ Consequently, reducing the ROS levels in OBs and OCs may curb bone destruction caused by GCs. The nuclear factor erythroid‐derived 2‐related factor‐2 (Nrf2) is recognized as an essential regulator of oxidant resistance, as it instigates enzymes that manage oxidative stress. Kelch ECH associating protein 1 (Keap1) is an Nrf2‐binding protein that facilitates Nrf2 degradation.^9^, ^10^ Prior studies have indicated that Keap1/Nrf2 signalling is a crucial regulator of bone homeostasis, primarily because Nrf2 activation intensifies the endogenous antioxidant response to ROS.^11^

In recent times, the exploration for efficient and secure antioxidants originating from vegetation has intensively increased. *Stephania* is a plant genus comprised of a wide variety of species rich in alkaloids which possess vast pharmacological properties.^12^ Crebanine (CN), an isoquinoline alkaloid extracted from *Stephania venosa*, exhibits promising pharmacological effects in terms of anti‐arrhythmic, Alzheimer's disease and analgesic activities.^13^, ^14^, ^15^ A study suggested that CN could inhibit inflammatory activity in LPS‐induced mouse macrophages via suppressing MAPKs and Akt signalling.^16^ Past research also investigated CN's capability to inhibit oxidative stress, which ameliorates ischaemia‐reperfusion brain damage, and triggering apoptosis in human hepatocellular carcinoma cells through a ROS‐dependent mechanism.^17^, ^18^ However, the biological effects of CN on OCs and OBs have not been defined yet.

With the swift advancement of bioinformatics, network pharmacology provides a systematic approach to understanding disease pathogenesis.^19^ By utilizing methods including target prediction from databases, Gene Ontology (GO), Kyoto Encyclopedia of Genes Genomes (KEGG) enrichment analyses and protein–protein interaction (PPI) networks, network pharmacology may be a valuable tool to explore the possible efficacy of CN in treating ONFH. Therefore, in this study, we used web‐based resources to elucidate the pharmacological network of CN in preventing and treating ONFH. Furthermore, we conducted experiments to verify that CN can mitigate GCs‐induced osteoclastogenesis and osteoblast damage via inhibiting ROS, thereby slowing the progression of ONFH in mice. This investigation may present a new strategy for molecular intervention targeting ONFH through the use of natural compounds.

## 2 MATERIALS AND METHODS

### 2.1 Bioinformatics data sources and network analysis

The structural information of CN was obtained by searching on ChemSpider (ChemSpider|Search and share chemistry). Predicted targets were identified from the Swiss Target Prediction database (http://www.swisstargetprediction.ch/) based on CN's structural information.^20^ Relevant targets associated with ONFH were gathered from the GeneCards database (https://www.genecards.org/) using ‘femoral head necrosis’ as the keyword.^21^ Subsequently, the Webgestalt database (www.webgestalt.org/) was employed for GO and KEGG enrichment analyses of the overlapping gene targets.^22^ The PPI network for these gene targets was then constructed using the String database (www.string‐db.org/).^23^

The molecular dockings were made by Vina software 1.1.2. Downloaded protein structures of Nrf2 and Keap1 from the Protein Data Bank (https://www.rcsb.org) and processed them using AutoDock Tools 1.5.6 along with the molecular structure of CN. The results were analysed and visualized using PyMOL software.

### 2.2 Reagents and antibodies

CN with a purity exceeding 99% was purchased from MCE. Cell culture reagents, including minimum essential medium α (α‐MEM; C12571500BT), fetal bovine serum (FBS; 10099141C) and penicillin/streptomycin (P/S; 15140122), were purchased from Gibco. Reagents for cell differentiation and stimulation included ascorbic acid (AA; A8960, Sigma–Aldrich), β‐glycerophosphate disodium (β‐GP; G9422, Sigma–Aldrich), receptor activator of nuclear factor‐κB ligand (RANKL; 462‐TEC‐010, R&D Systems), dexamethasone (DEX; HY‐14648, MCE), macrophage colony‐stimulating factor (M‐CSF; 416‐ML‐050, R&D Systems) and *N*‐acetylcysteine (NAC; A7250, Sigma–Aldrich). Primary antibodies used in western blotting (WB), such as OPN (22952‐1‐AP; 1:1000), Keap1 (10503‐2‐AP; 1:1000) and Nrf2 (16396‐1‐AP; 1:1000) were purchased from Proteintech. Antibodies for Col1α1 (72026s; 1:1000) and RUNX2 (8486; 1:1000) detection were sourced from Cell Signaling Technology; for NFATc1 (A1539; 1:1000) detection was from Abclonal; for Ctsk (sc‐48353; 1:1000) detection were from Santa Cruz. All secondary antibodies including anti‐mouse (SA00001‐1; 1:3000) and anti‐rabbit (SA00001‐2; 1:3000) were purchased from Proteintech.

### 2.3 Cell culture

The cell culture methods were similar to those used in our previous study.^24^ In brief, bone marrow‐derived monocytes (BMMs) were obtained from C57BL/6J male mice aged from 8 to 10 weeks. Bone marrow cells, flushed out from the femur, were cultured in a complete medium (CM; α‐MEM with 1% P/S and 10% FBS) for 16–24 h at 37°C in a 5% CO_2_ condition. Red blood cells (RBCs) were removed using a RBC Lysis Buffer (BL503A; Biosharp), and the remaining cells were cultured at a density of 2 × 10^5^ cells/mL in CM supplemented with M‐CSF (25 ng/mL) for 2 days. The adherent cells were then treated with osteoclastogenesis medium (CM with 10 ng/mL M‐CSF and 40 ng/mL RANKL) for a five‐day differentiation period. Alternatively, the cells could be stimulated with osteoclastogenesis medium and DEX in CM for a five‐day differentiation period to simulate the microenvironment of ONFH. The culture medium was replaced daily.

Osteoblast precursor cells (OPCs) were isolated from the cranium of C57BL/6J mice during postnatal days 3–5. The cranium was immersed in a collagenase solution, composed of collagenase II (2 mg/mL) (Gibco, 17101015) and 0.25% Trypsin (Gibco, 15090046), for 20 min. Afterwards, the collagenase solution was replaced, and the cranium was cut into small portions and incubated for another 20 min, followed by the addition of α‐MEM with 15% FBS and 1% P/S. After 24 h, the medium was replaced by the CM. For osteogenesis induction, cells from third to fifth generation were used and cultured at 5 × 10^4^ cells/mL in culture plates. When the cells reached 80% confluency in plates, the CM was replaced with osteogenic induction medium (OIM; CM with 10 mM β‐glycerophosphate disodium salt hydrate, 50 μg/mL ascorbic acid, and 100 nM DEX), with or without 10 μM DEX to damage OBs.^25^, ^26^ The OIM was replaced every 3–4 days.

### 2.4 Cell viability assay

BMMs and OPCs were cultured in 96‐well plates, and cell viability was determined using the Enhanced Cell Counting Kit 8 (CCK‐8; E‐CK‐A362, Elabscience) assay. BMMs were exposed to various concentrations (0, 2.5, 5, 7.5, 10, 12.5 and 15 μM) of CN in CM containing 10 ng/mL M‐CSF, with or without 40 ng/mL RANKL, for a duration of 48 h. Similarly, OPCs were stimulated with different concentrations of CN (0, 1, 2, 3, 4 and 5 μM) in CM or OIM over the same 48 h period. Subsequently, CCK‐8 was added to each well, and then cells were incubated at 37°C for 1–2 h away from light, followed by the absorbance measurement via a Microplate Photometer (Multiskan FC; ThermoFisher Scientific) at a wavelength of 450 nm.

### 2.5 Tartrate‐resistant alkaline phosphatase (TRAP) staining

TRAP staining was performed as described previously with slight modifications.^25^, ^27^ The acetate‐tartrate buffer composed of sodium acetate trihydrate (19 mg/mL), glacial acetic acid 100% (0.45%) and sodium tartrate trihydrate (150 μg/mL) in Milli‐Q water. Then dissolved the Fast violet B salt (7 mg/mL) in the acetate‐tartrate buffer and filtered through a 0.45‐μm filter after agitation. Naphthol solution was prepared with 2 mg/mL of naphthol AS‐TR phosphate disodium salt dissolved in the acetate‐tartrate buffer. Finally, equal volumes of Fast Violet B solution and naphthol solution were mixed completely to form the TRAP staining solution. OCs were fixed with 4% paraformaldehyde (PFA; BL539A, Biosharp) for 20 min and then stained with the TRAP staining solution for 30 min at 37°C. Impurities were later eliminated using a sodium fluoride solution. TRAP‐positive cells containing three or more nuclei were defined as mature OCs, and their numbers were counted via light microscopy (10 visual fields were observed for each group).

### 2.6 F‐actin staining

F‐actin staining was accomplished as previously described.^28^ Cells were fixed with PFA for 20 min, followed by staining with Phalloidin‐iFlour 594 (ab176757, Abcam) in light‐avoiding conditions at room temperature, for a period of 45 min. Subsequently, the nuclei were counterstained with 4′,6‐diamidino‐2‐phenylindole (DAPI; C0065, Solarbio) for 5 min. The structures of the actin ring were imaged using a fluorescence microscope (EVOS M7000; ThermoFisher), which randomly selects multiple fields of view to identify F‐actin rings based on the characteristic circular structures bordering the OCs periphery, and assess the structure and integrity of these F‐actin rings. The quantitative analysis of the F‐actin ring ratio was calculated by dividing the number of OCs (containing more than three nuclei) with complete F‐actin rings by the total number of OCs.

### 2.7 Acridine orange (AO) staining

AO staining was carried out as previously described with slight modifications.^27^, ^28^ BMMs were cultured and induced in 24‐well plates until mature OCs were generated. These OCs were then incubated with a solution of AO (A6014; Sigma–Aldrich), diluted in α‐MEM at a working concentration of 10 μg/mL, at 37°C for a duration of 15 min. After three rounds of washing with α‐MEM, the acid vesicles of OCs were visualized using a fluorescence microscope (EVOS M7000; ThermoFisher). Fluorescent staining images were used for qualitative analysis. Subsequently, a microplate reader (TECAN SPARK, TECAN) has been utilized for quantitative analysis by measuring the ratio of the absorbance at a wavelength of 625 nm (red light) over 485 nm (green light).

### 2.8 ALP staining and alizarin red (AR) staining

ALP and AR staining was performed as described previously with slight modifications.^24^, ^28^ OPCs were seeded into 24‐well plates and cultured in OIM for 3–5 days to conduct ALP staining or 21–28 days to perform AR staining. After washing with PBS and then fixing with PFA for 20 min, cells were subjected to BCIP/NBT alkaline phosphatase colour development kit (ALP; C3206, Beyotime) or alizarin red S solution (1%, pH 4.2) (ARS; G1452, Solarbio) for a duration of 10 min at room temperature in dark conditions. All images were captured using a camera phone.

### 2.9 Intracellular ROS measurement

Intracellular ROS measurement was accomplished as previously described with slight modifications.^28^ Intracellular ROS level was determined using the Reactive Oxygen Species Assay Kit (DCFH‐DA; s0033, Beyotime). BMMs and OPCs were cultured in 24‐well plates. BMMs were stimulated with osteoclastogenesis medium and DEX (10^−8^ M), and treated with or without CN for a period of 24 h, whereas OPCs were stimulated with OIM and DEX (10^−5^ M), and treated with or without CN for a period of 48 h. Subsequently, cells were incubated in phenol red‐free α‐MEM, containing 10 μM DCFH‐DA probe, for 20 min at 37°C. After washing with phenol red‐free α‐MEM, the fluorescence images were captured using a Nikon fluorescence microscope, and the fluorescence intensity was quantified at wavelengths of 488–525 nm via a microplate reader (TECAN SPARK, TECAN).

### 2.10 Western blotting (WB)

WB was performed as described previously.^20^, ^24^ Cultured cells from various treatment groups were washed twice with pre‐cooled PBS, and then lysed by RIPA lysis buffer (R0020; Solarbio) supplemented with protease inhibitor (CW2200; Cwbio) and phosphatase inhibitors (CW2383; Cwbio). After undergoing centrifugation at 13,500 rpm for 20 min, the supernatant of the cells was collected. The protein concentration was determined using the BCA Protein Assay Kit (PC0020; Solarbio). Quantified proteins (20 μg) were combined with a fifth volume of 5 × SDS loading buffer and heated to 95°C for 5 min. The proteins were then separated by 10% SDS–PAGE gel and transferred to a 0.2 μm polyvinylidene difluoride membrane (PVDF, ISEQ00010, Merk Millipore). The membranes were then blocked using 5% non‐fat skim milk at room temperature for 1 h, before being co‐incubated with primary antibodies overnight at 4°C: NFATc1, Ctsk, Calm, PP2B‐Aα, RUNX2, OPN, Col1α1, Nrf2 and Keap1. After washing with Tris Buffered Saline (TBS; G0001, Servicebio) the membranes were incubated with a secondary antibody for 1–2 h at room temperature. The protein bands were visualized using the Clarity Western ECL Substrate (1705061; Biorad), and the relative grey values were analysed using ImageJ software.

### 2.11 Reverse transcription quantitative polymerase chain reaction (RT‐qPCR)

Following 3 days of OCs induction or 7 days of OBs induction, total RNA of the cells was extracted using RNAiso Plus (9109, Takara). According to manufacturer's instruction, cDNA was synthesized by the RT reaction of total RNA using PrimeScript™ RT Reagent Kits (RR047A, Takara). qPCR was performed by using the SYBR Green PCR kit (AG11701, Accurate Biology) in a RT fluorescence quantitative PCR system (LightCycler® 96 SW 1.1, Roche Ltd). The obtained values were then normalized to the GAPDH levels and analysed via 2‐^ΔΔ^Ct method. The primer sequences used in the study are listed in Table S1.

### 2.12 Glucocorticosteroid (GC)‐induced murine femoral head necrosis model

The establishment of animal models of GC‐induced ONFH was performed as previous studies.^29^, ^30^ Fifteen male C57BL/6J mice at 8 weeks old were used for in vivo study and housed at SPF condition, provided with food and drinking water in an environment at 24 ± 2°C and a 12 h light/dark cycle. These mice were randomly allocated into three groups: the control group (*n* = 5), the methylprednisolone (MPS) group (*n* = 5) and the MPS + CN group (*n* = 5). The mice from the MPS group underwent intramuscular injections around the hip joint of MPS (20 mg/kg), while the MPS + CN group was subjected to intramuscular injections of MPS (20 mg/kg) and CN (5 mg/kg). The control group received an equivalent amount of saline thrice per week over a duration of 3 weeks. Mice were harvested using CO_2_ for 6 weeks following the model's establishment. Prior to being euthanized, mice were intraperitoneally injected with calcein (20 mg/kg) 10 days and 3 days before. The femoral bone tissues were collected, fixed by 4% PFA and kept at 4°C PBS pending further analysis.

### 2.13 Micro‐CT scanning

A quantitative analysis of the microarchitecture of the femoral head was conducted with the micro‐CT system (Quantum GX2, PerkinElmer). The complete femoral head tissue from mice of each group were fixed with 4% PFA for 24 h, rinsed with PBS and immersed in 70% alcohol to save. The femoral head were scanned with the following settings: High resolution scan mode, voltage 70 kV, current 114 μA and voxel size 144 μm. The proximal femur was quantified as the region of interest, analysed 0–270 slices and evaluated to determine percentage of bone volume (BV/TV, %), trabecular number (Tb.N, 1/mm), trabecular separation (Tb.Sp, mm) and trabecular thickness (Tb. Th, mm) as previously descried with slight modifications.^24^

### 2.14 Histological analysis

After 12 h of dehydration with 20% sucrose, the femoral head tissue were then embedded with an optimum cutting temperature compound (OCT; 4583, Sakura) and sectioned at 6 μm thickness using a freeze microtome (Minux FS800; RWD). Subsequently, these femoral head sections were stained for 5 min using TRAP staining solution, followed by counterstaining for 5 min with Weigert's haematoxylin (G1142, Solarbio). Images were captured using a Nikon optical microscope.

### 2.15 Statistical analysis

All quantitative data were presented as mean ± standard deviation (SD) derived from biological triplicate results. For the comparison of multiple data sets, a one‐way analysis of variance was employed. Statistical significance was set at *p* < 0.05. All statistical analyses were performed using the GraphPad Prism 8.0 software (GraphPad Software Inc).

## 3 RESULTS

### 3.1 Potential targets and biological pathways of CN in the prevention and treatment of ONFH

**FIGURE 1:**
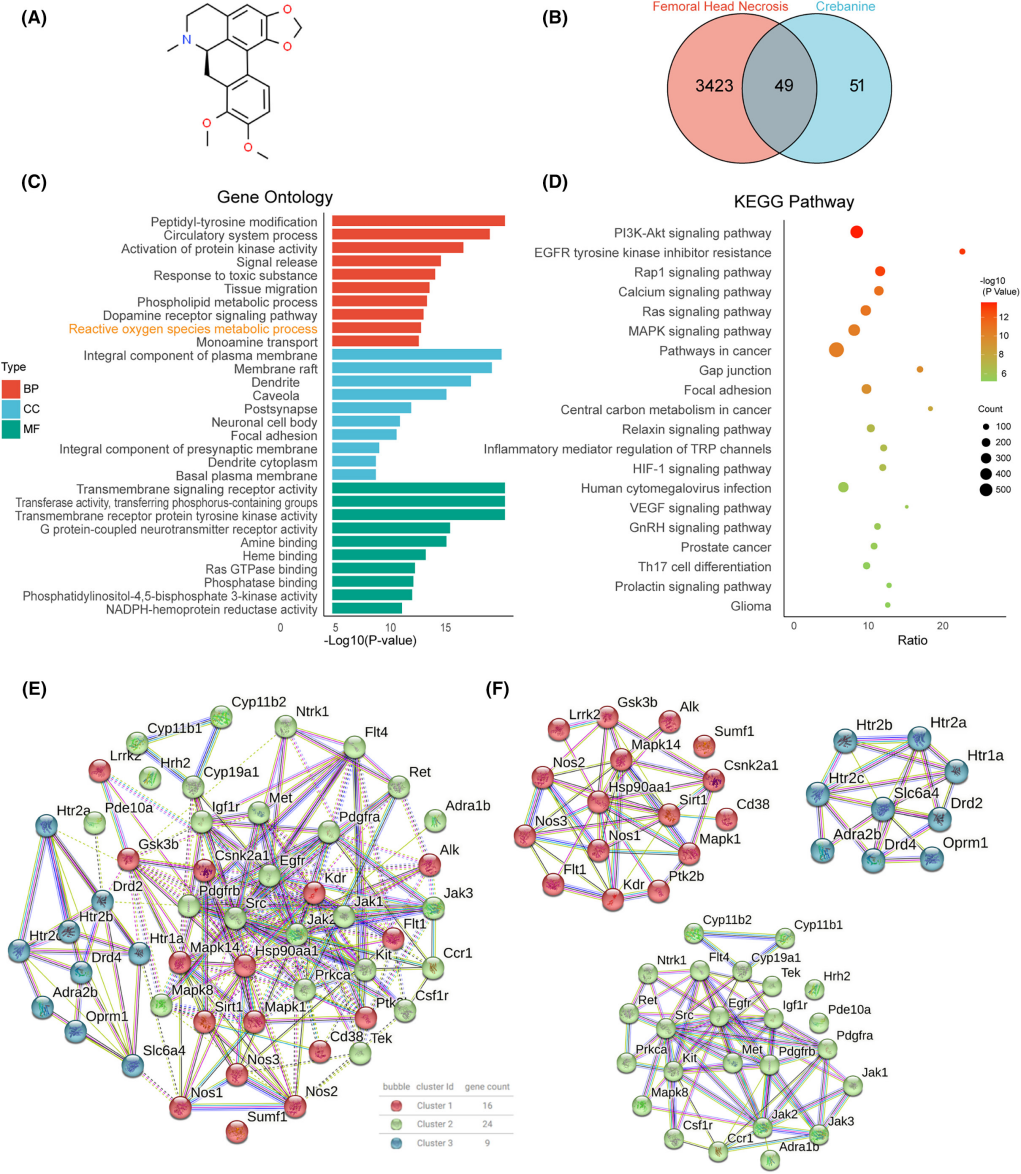
Potential targets and biological pathways of CN in the prevention and treatment of ONFH. (A) CN chemical structure. (B) Venn diagram summarized CN's candidate targets for ONFH. (C) GO enrichment analysis of CN, including the top 10 terms of BP, CC and MF. (D) Top 20 terms of KEGG pathway enrichment. (E, F) PPI network and cluster annotation of CN.

First, we conducted a network pharmacological analysis to predict the potential molecular mechanisms utilized by CN in preventing and treating ONFH (Figure 1A). Our results led to the identification of 100 potential genes from the Swiss Target Prediction database, utilizing CN's molecular structure (Table S2), and yielded in the retrieval of 3472 human genes linked to osteonecrosis from the GeneCards database using the term ‘femoral head necrosis’ (Table S3). We proceeded to cross‐reference these genes with potential CN targets and found CN within the set of ONFH‐related human genes. Forty‐nine genes that intersected were consequently chosen as putative targets for the intervention of ONFH (Figure 1B).

We subsequently employed Webgestalt to explore the biological attributes of these potential CN targets. Delineation of biological processes and metabolic pathways was accomplished via GO annotation and KEGG enrichment analyses. The top 20 terms revealed in the KEGG enrichment analysis, together with the leading 10 terms underscored in each category, including biological process (BP), cellular component (CC) and molecular function (MF), satisfied the statistical significance criteria, marked by a count ≥2 and a *p*‐value <0.01. The GO analysis outcomes indicated that CN may regulate the metabolic process of ROS through activities of transmembrane signalling receptors and transferases that are located in the plasma membrane (Figure 1C). Moreover, the KEGG pathway enrichment and PPI unveiled a potential correlation between CN with PI3K‐Akt signalling pathway, calcium signalling pathway, MAPK signalling pathway and redox regulation (Figure 1D–F). Previous studies have confirmed the close correlation of these signalling pathways with the biological functions of OCs and OBs.^31^, ^32^, ^33^ In addition, our previous research has also confirmed that redox metabolism has significant effects on OCs and OBs.^27^, ^28^ Therefore, we hypothesize that CN exerts a therapeutic effect on ONFH by affecting OCs or OBs. These findings imply a role for CN in ONFH treatment, underscoring its therapeutic potential.

### 3.2 CN inhibits DEX‐stimulated osteoclastogenesis

**FIGURE 2:**
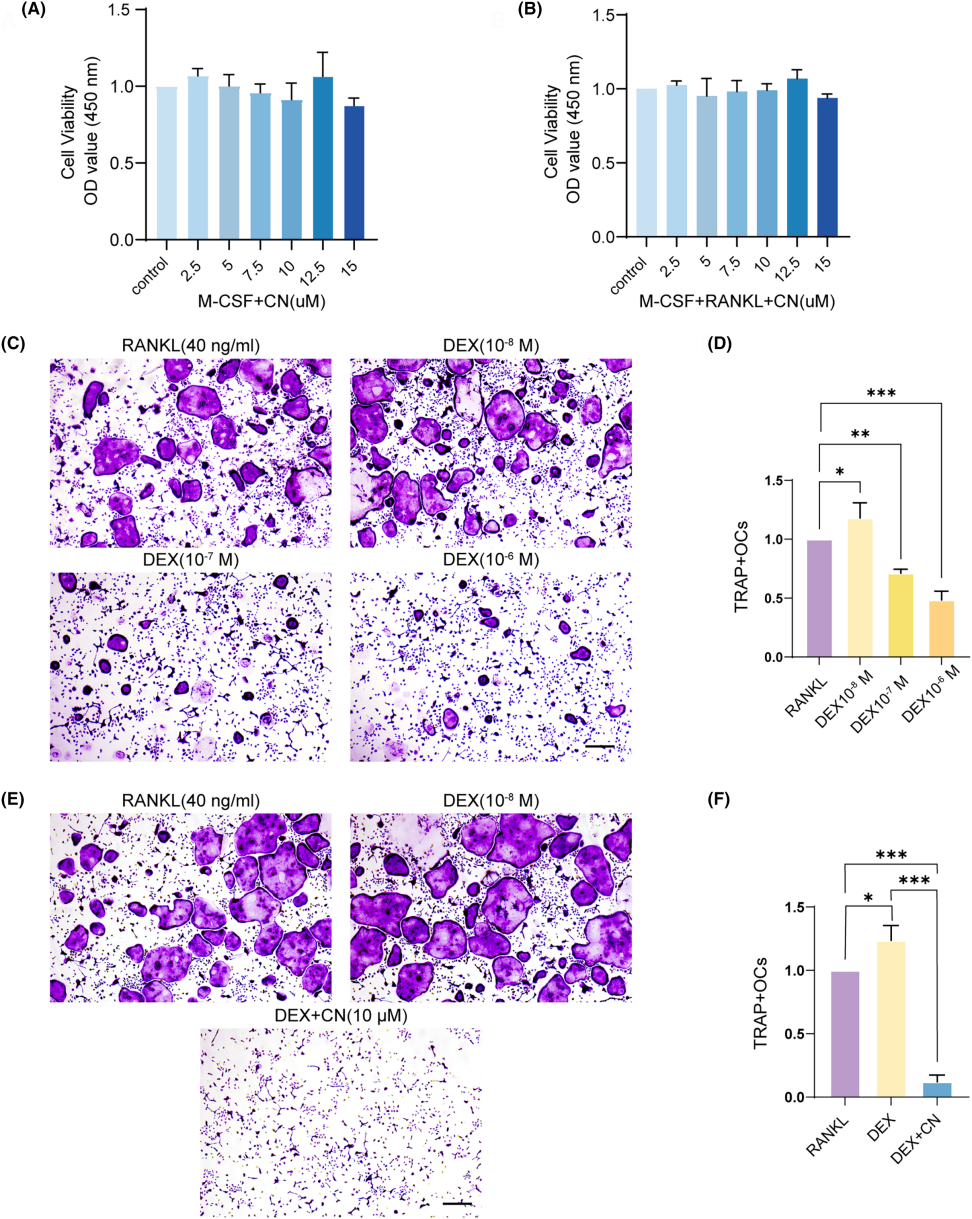
CN inhibits DEX‐stimulated osteoclastogenesis. (A, B) CCK‐8 assay for cytotoxicity test. BMMs were subjected to various concentrations of CN in complete medium (CM) containing M‐CSF (10 ng/mL) for 48 h (*n* = 3); (B) BMMs were subjected to varying concentrations of CN in osteoclastogenesis medium for 48 h (*n* = 3). (C) TRAP staining of osteoclasts (OCs) under different stimuli. BMMs were cultured in osteoclastogenesis medium alone or combined with various dexamethasone (DEX) concentrations for a five‐day differentiation. Scale bars = 200 μm. (D) Quantitative analysis of TRAP‐positive cells (containing more than three nuclei) (*n* = 3). Data were presented as mean ± SD. *p < 0.05, ***p < 0.001. (E) TRAP staining of OCs under CN treatment. BMMs were cultured in osteoclastogenesis medium for a five‐day differentiation, or cultured in osteoclastogenesis medium and DEX (10^−8^ M) for a five‐day differentiation with or without CN (10 μM). Scale bar = 200 μm. (F) Quantitative analysis of TRAP‐positive cells (*n* = 3). Data were presented as mean ± SD. *p < 0.05, **p < 0.01, ***p < 0.001.

To verify the therapeutic potential of CN in treating ONFH from osteoclastic perspective, we firstly conducted a CCK‐8 cell viability experiment to ascertain the optimal dosage of CN for BMMs. BMMs were exposed to various concentrations of CN (2.5, 5, 7.5, 10, 12.5 and 15 μM) in the presence of M‐CSF and in conditions with or without RANKL for a period of 24 h. The outcomes of the CCK‐8 assay results suggested that CN did not display significant cytotoxic effects on BMMs at concentrations below 15 μM (Figure 2A,B). Therefore, in subsequent experiments, 10 μM of CN was administered to BMMs that had been treated with DEX. To discern the appropriate DEX concentration for the stimulation of osteoclastogenesis, BMMs were exposed to different DEX dosages (10^−8^, 10^−7^ and 10^−6^ M) under M‐CSF and RANKL condition. TRAP staining revealed that at a concentration of 10^−8^ M, DEX promoted osteoclast formation, whereas it impeded this process at concentrations greater than 10^−7^ M (Figure 2C,D). This finding corroborates previously conducted studies.^34^, ^35^ Consequently, we induced BMMs with 10^−8^ M DEX to emulate the microenvironment of ONFH. Surprisingly, following CN treatment, our results revealed a considerable decrease in OC formation (Figure 2E,F), thereby signifying a notably inhibitory impact of CN on osteoclastogenesis.

### 3.3 CN inhibits the resorption activity of OCs

**FIGURE 3:**
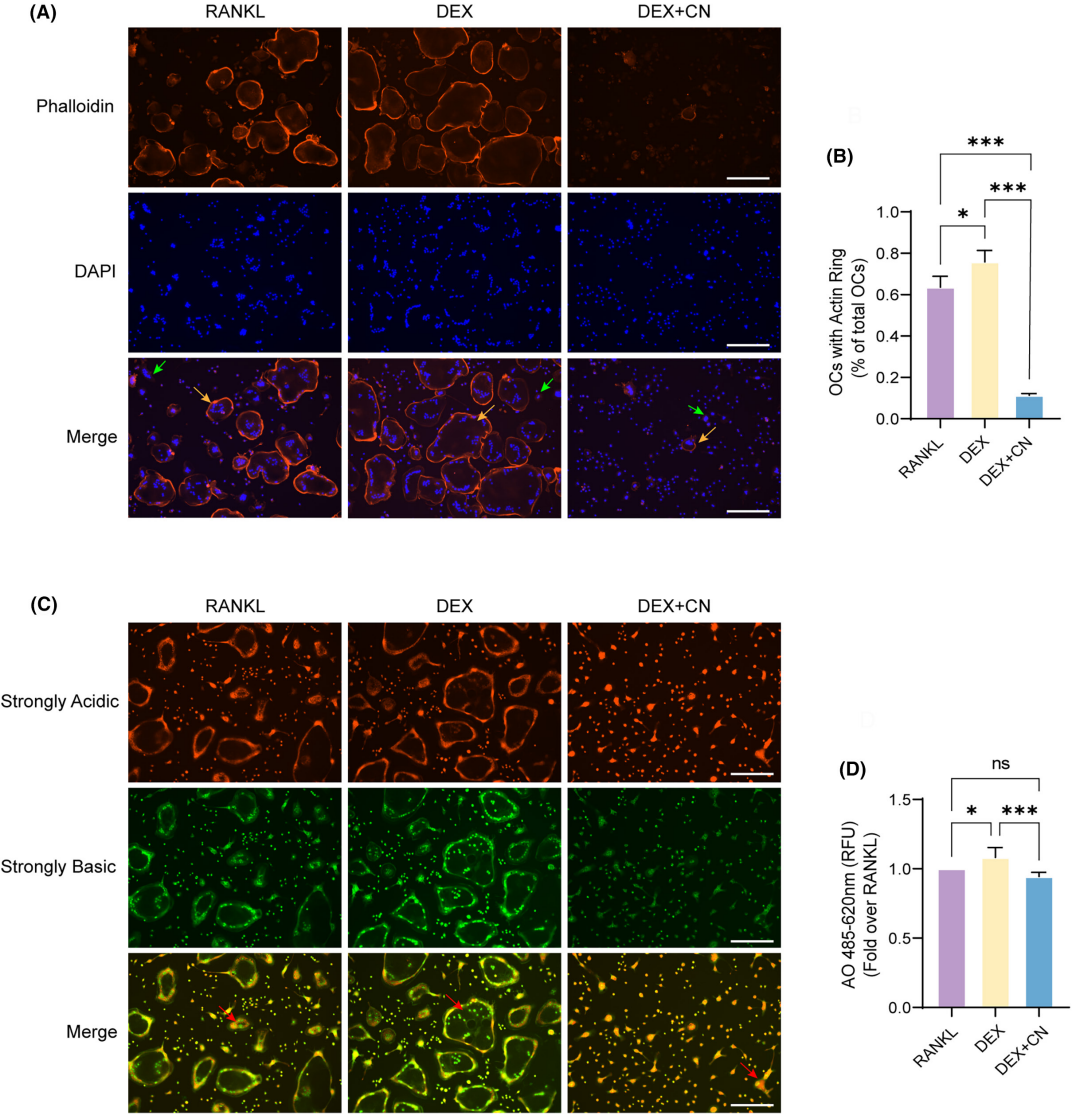
CN inhibits the resorption activity of OCs. (A) F‐actin ring staining. The F‐actin rings were observed by fluorescence microscopy subsequent to staining with Phalloidin‐iFlour 594 and DAPI. Scale bar = 100 μm. Yellow arrows mark OCs with intact F‐actin rings, and green arrows mark OCs that have not formed F‐actin rings. (B) The proportion of OCs with F‐actin rings (*n* = 3). Data were presented as mean ± SD. *p < 0.05, ***p < 0.001. (C) AO staining. The acid vesicles of OCs were observed through a fluorescence microscope following staining with AO dye. Scale bar = 100 μm. Red arrows mark acidic vesicles. (D) Quantitative analysis of fluorescence intensity was measured by a microplate reader (Ex/Em = 485/520 nm for green and Ex/Em = 485/625 nm for red) (*n* = 3). Data were presented as mean ± SD. *p < 0.05, ***p < 0.001, ns, no significance.

Subsequently, to validate the effect of CN on OC functionality, the formation of the F‐actin ring and the ability of osteoclastic acidification were evaluated, representing essential prerequisites for bone resorption. We undertook Phalloidin‐iFlour 594 and AO staining for the detection of the F‐actin ring and acidic vesicle, respectively. The results from Phalloidin‐iflour 594 staining revealed that 63% of the OCs stimulated by RANKL and 76% by DEX had intact actin rings. However, the inclusion of CN at a concentration of 10 μM demonstrated a reduction in ring formation to 11% (Figure 3A,B). Concurrently, AO staining illustrated a diminish in the red‐to‐green fluorescence ratio in the OCs belonging to the CN group (Figure 3C,D). This consequently suggests an impaired capacity for acid secretion and matrix dissolution in the CN‐treated OCs, thereby suggesting an inhibitory effect of CN on the resorption function of OCs.

### 3.4 CN downregulates OC marker gene expression

**FIGURE 4:**
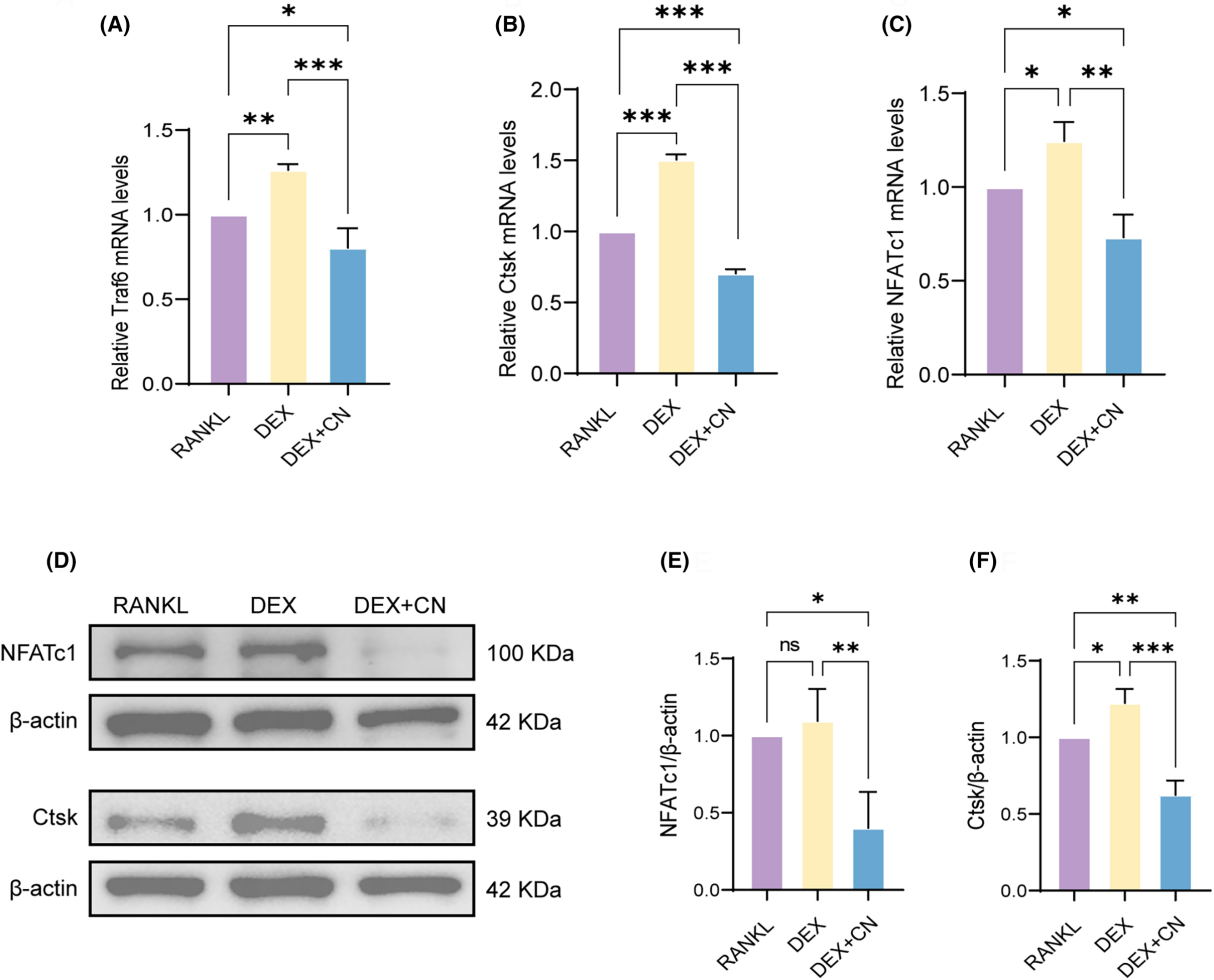
CN downregulates OC marker genes expression. (A–C) mRNA levels of Traf6, Ctsk and NFATc1 of OCs under different culture conditions, normalized to GAPDH expression. Data were presented as mean ± SD (*n* = 3). *p < 0.05, **p < 0.01, ***p < 0.001. (D) WB analysis of the expression of Ctsk, NFATc1 and β‐actin in OCs under different culture conditions. (E, F) Quantitative results were normalized to β‐actin (*n* = 3). Data were presented as mean ± SD (*n* = 3). *p < 0.05, **p < 0.01, ***p < 0.001.

To achieve a more profound comprehension of the biological mechanisms underlying the inhibitory effects of CN on DEX‐stimulated OCs function, we examined changes in OC markers at the transcriptomic and proteomic levels. As demonstrated by the results of RT‐qPCR, the CN‐treated group showed notable decreases in the expression of OC marker genes associated with both differentiation (NFATc1, Traf6) and function (Ctsk) (Figure 4A–C). WB analyses further substantiated these findings, confirming reduced expression of both NFATc1 and Ctsk at protein levels following CN treatment (Figure 4D–F). Collectively, these results suggest that CN effectively inhibits DEX‐stimulated OC differentiation and function.

### 3.5 CN alleviates DEX‐induced osteogenic damage and reverses DEX‐induced reduction of osteoblast marker genes

**FIGURE 5:**
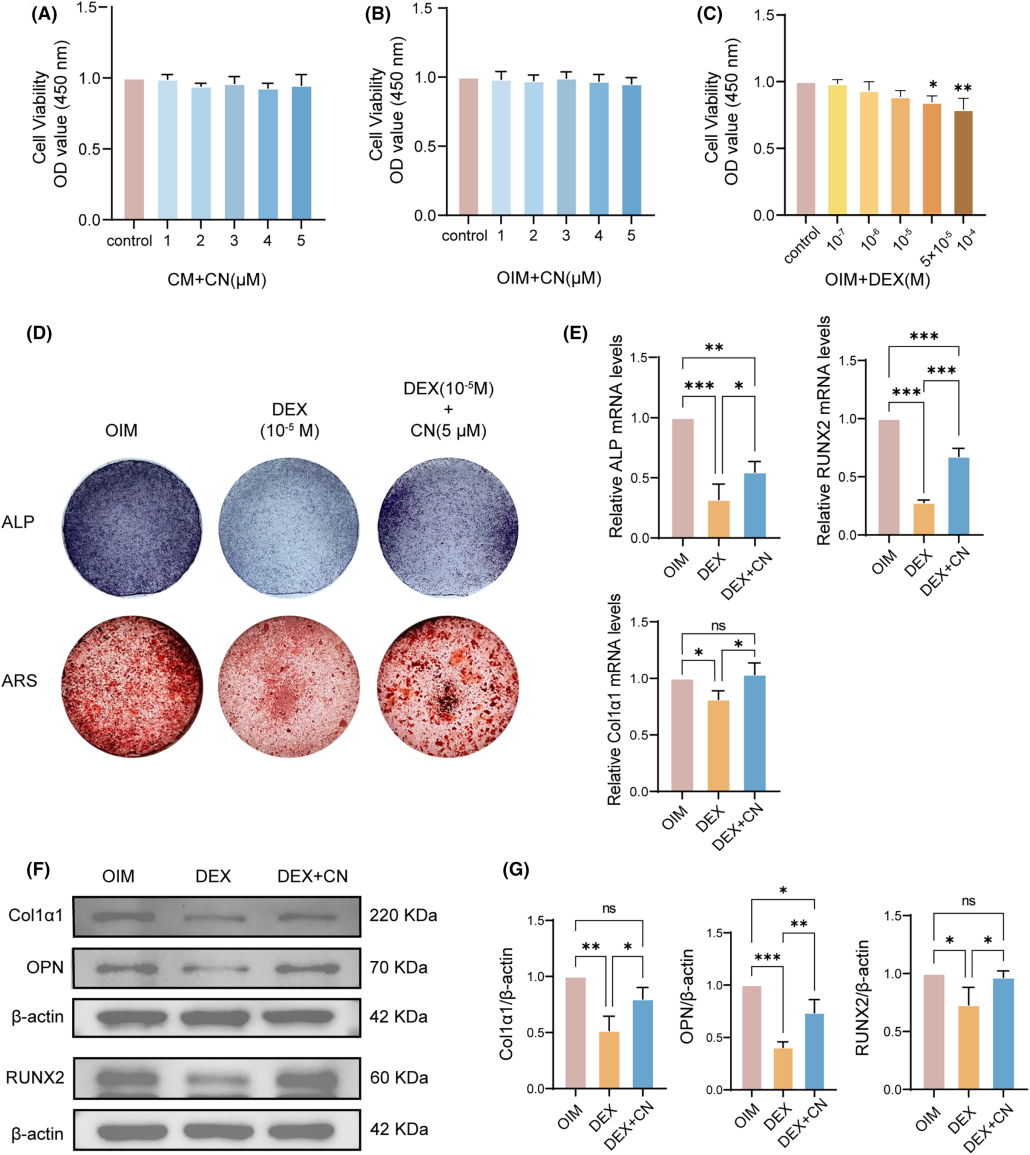
CN alleviates DEX‐induced osteogenic damage and reverses DEX‐induced reduction of osteoblast marker genes. (A–C) CCK‐8 assay for cytotoxicity test. Osteoblast precursor cells (OPCs) were subjected to various dosages of CN in complete medium (CM) or osteogenic induction medium (OIM) for 48 h, or with different dosage of DEX in OIM for 48 h (*n* = 3). (D) ALP and ARS staining of osteoblasts (OBs) under various conditions. OPCs were cultured in OIM with or without DEX stimulation for 3 or 28 days, with the addition of 5 μM CN to the designated groups. Staining results were taken by a camera with 1× magnification. (E) mRNA levels of ALP, RUNX2 and Col1α1 of OBs under different culture conditions, normalized to GAPDH expression. Data were presented as mean ± SD (*n* = 3). *p < 0.05, **p < 0.01, ***p < 0.001. (F) WB analysis of the expression of RUNX2, OPN, Col1α1 and β‐actin in OBs under different culture conditions. (G) Quantitative results were normalized to β‐actin (*n* = 3). Data were presented as mean ± SD (*n* = 3). *p < 0.05, **p < 0.01, ***p < 0.001.

In order to discern if CN also influences another aspect of bone remodelling, we explored its impact on osteogenic differentiation and started with assessing variations in cell viability at different CN concentrations via a CCK‐8 assay. The results revealed that CN did not portray significant cytotoxicity towards OPCs at concentrations below 5 μM (Figure 5A,B). To discern the appropriate DEX concentration for the suppression of osteogenesis, OPCs were induced in OIM and exposed to different DEX dosages (10^−7^, 10^−6^, 10^−5^, 5 × 10^−5^ and 10^−4^ M). The outcomes of the CCK‐8 assay results indicated that DEX did not display significant cytotoxic effects on OPCs at concentrations below 10^−5^ M (Figure 5C). The impact of CN on the osteogenic process under DEX circumstances was then examined. OPCs were stimulated with OIM, either with or without 10^−5^ M DEX, and examined the ALP expression and calcium nodule formation. Our results revealed that the application of CN at 5 μM concentration significantly mitigated the inhibitory effect initiated by DEX on ALP activity and formation of calcium nodules (Figure 5D). This suggests that CN potentially alleviates the damage to osteogenesis caused by DEX.

To gain a deeper insight into the biological mechanisms underlying the cellular phenotype, we investigated changes in OB‐specific markers at both transcriptomic and proteomic levels. The results of RT‐qPCR demonstrated a downregulation of OB markers associated with both differentiation (RUNX2, ALP) and function (Col1α1) at the mRNA level under DEX conditions. However, CN treatment could partially mitigate the extent of this downregulation (Figure 5E). Additionally, the trends observed at the protein level pertaining to all marker genes in the CN group were congruent with those seen at the RNA level (Figure 5F,G). These findings provide support for the hypothesis that CN may play a beneficial role in mitigating DEX‐induced osteogenic damage.

### 3.6 CN reduces DEX‐induced oxidative stress in both OCs and OBs

**FIGURE 6:**
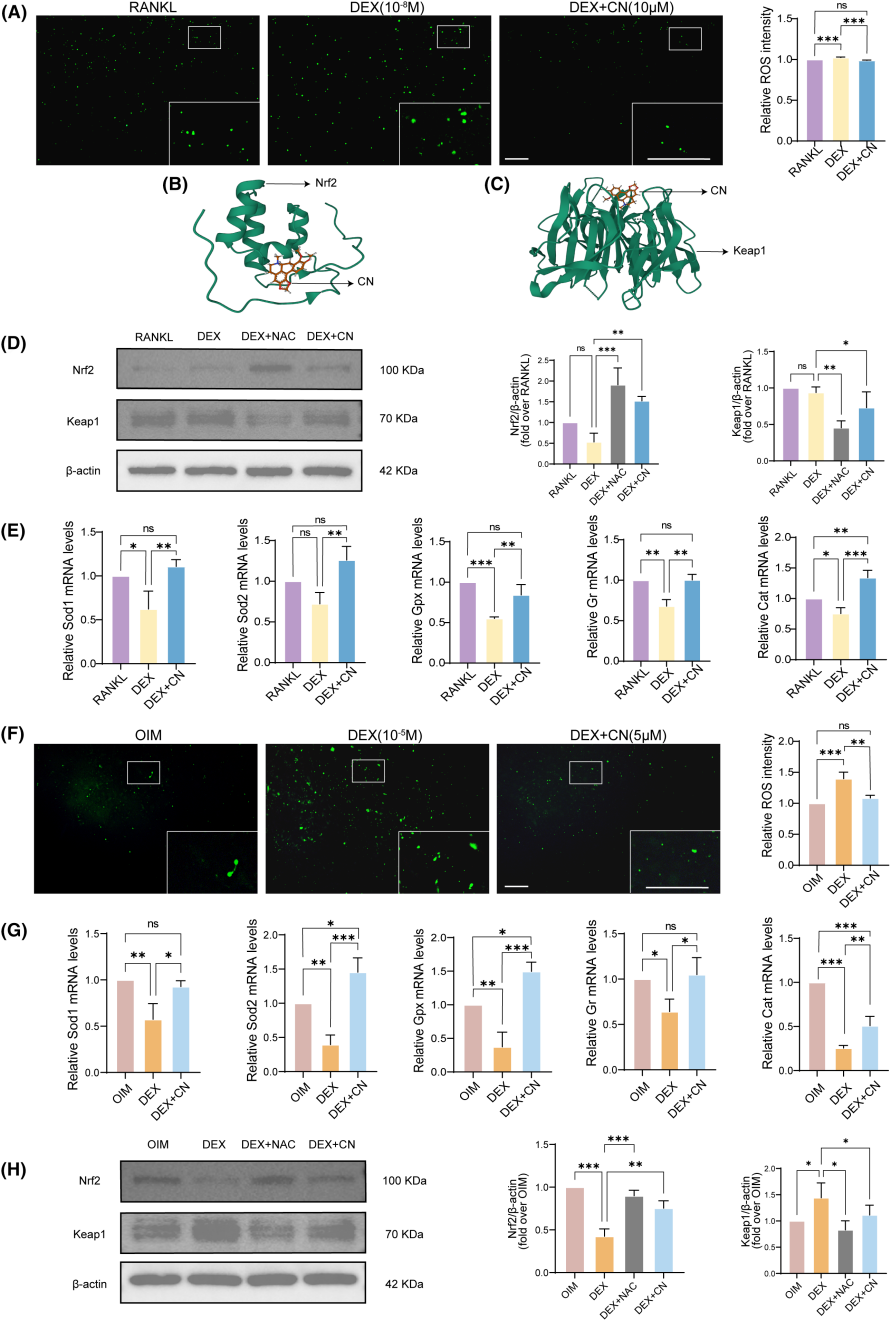
CN reduces DEX‐induced oxidative stress in both OCs and OBs. Molecular docking diagram between CN and Nrf2 (B) or Keap1 (C). Representative images of DCFH‐DA staining of BMMs (A) or OPCs (F) under various treatments. The intracellular ROS intensity in BMMs or in OPCs were quantified using a microplate reader (Ex/Em = 488/525 nm). Scale bar = 200 μm. Relative mRNA levels of antioxidant enzymes in OCs (E) or in OBs (G) under different culture conditions, normalized to GAPDH expression. Data were presented as mean ± SD (*n* = 3). *p < 0.05, **p < 0.01, ***p < 0.001. WB analysis of the expression of Nrf2, Keap1 and β‐actin in OCs (D) or in OBs (H) under different culture conditions. Quantitative results were normalized to β‐actin (*n* = 3). Data were presented as mean ± SD (*n* = 3). *p < 0.05, **p < 0.01, ***p < 0.001.

Since ROS have been established as essential for the biological functions of both OCs and OBs,^7^, ^8^ and our bioinformatics data also indicated that CN may be involved in the regulation of ROS metabolism (Figure 1C), we therefore investigated whether the impact of CN on OCs and OBs is regulated by cellular redox state. The results of DCFH‐DA staining demonstrated that DEX stimulation significantly increased intracellular ROS generation in both BMM and OPC cells, which was substantially diminished following CN treatment (Figure 6A,F). To determine if the observed ROS reduction induced by CN is associated with the Nrf2‐triggered antioxidant system, molecular docking techniques were utilized and demonstrated that there were potential binding sites between CN and Nrf2 or Keap1 (Figure 6B,C). Meanwhile, we added the ROS scavenger NAC (4 mM) to further confirm that CN attenuates the effects of DEX by inhibiting oxidative stress. Next, we assessed the protein levels of Nrf2 and Keap1, alongside the mRNA levels of several downstream antioxidant enzymes. The WB results revealed a substantial increase in Nrf2 expression and reduction in Keap1 expression in CN‐treated OCs (Figure 6D). For OBs, Nrf2 expression was also elevated in response to CN treatment even under DEX condition, but did not reach an equal level as compared with the OIM group (Figure 6H). Further, RT‐qPCR analysis from both OCs and OBs exhibited a decrease in specific antioxidant levels under DEX condition, which were restored following the treatment of CN (Figure 6E,G). Taken together, these findings suggest CN's potentiality in modulating the cellular redox state by attenuating the DEX‐induced ROS formation.

### 3.7 CN prevents glucocorticoid‐induced ONFH in vivo

**FIGURE 7:**
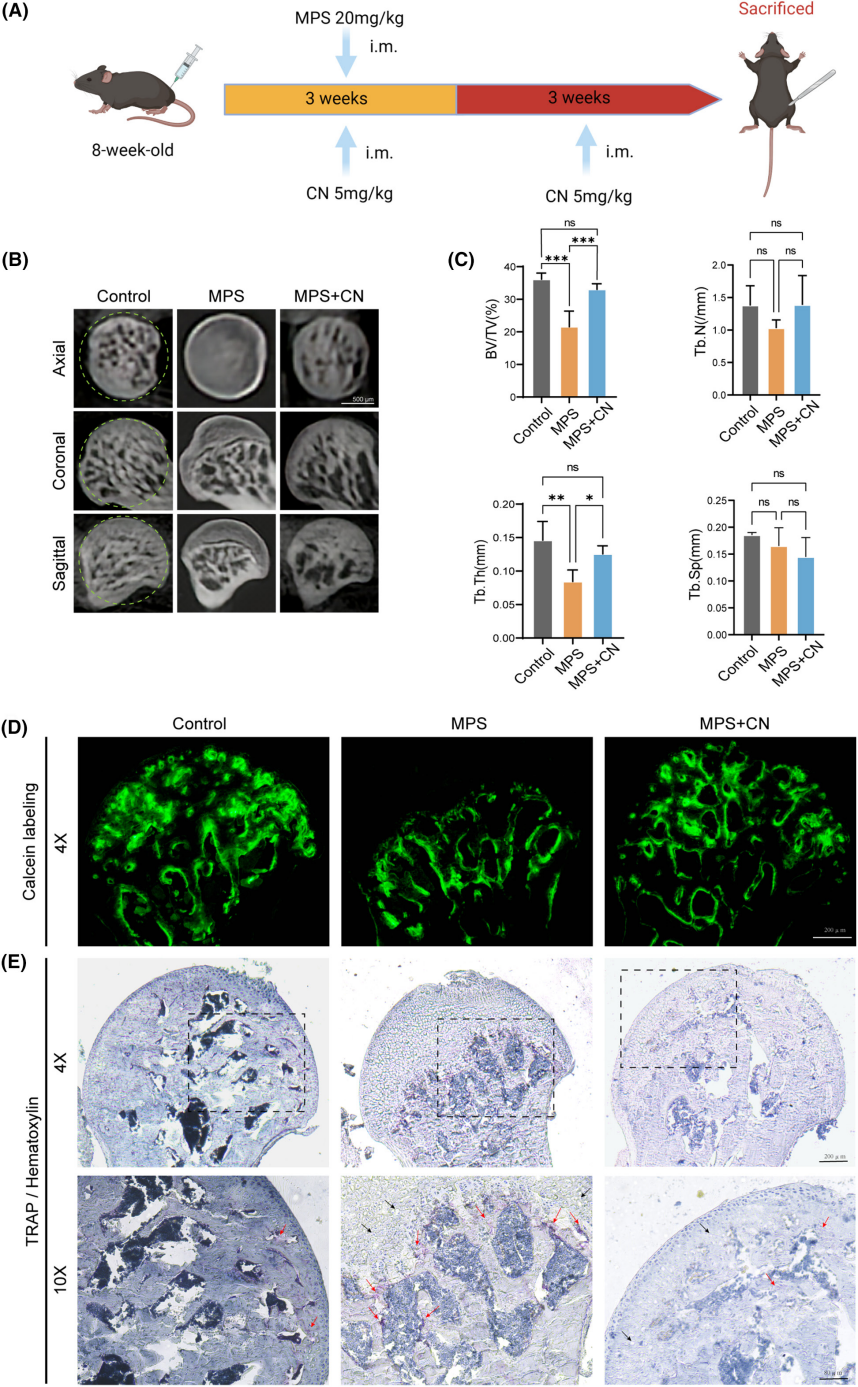
CN prevents glucocorticoid‐induced ONFH in vivo. (A) The chronological sequence of the in vivo establishment of glucocorticosteroids‐induced ONFH model and CN administration. (B) Representative images of micro‐CT. The green dotted line marks the ROI. Scale bar = 500 μm. (C) Quantitative analyses of bone parameters, including bone volume fraction (BV/TV), trabecular number (Tb.N), trabecular separation (Tb.Sp) and trabecular thickness (Tb.Th) (*n* = 5 per group). (D) Histomorphometry of the femoral head were determined through calcein labeling (*n* = 5 per group). Scale bar = 200 μm. (E) TRAP staining and haematoxylin staining of the femoral head. Black arrows indicate vacant osteocyte lacunae, red arrows denote TRAP‐positive cells. Scale bar = 200 μm (4×) or 80 μm (10×). MPS, methylprednisolone.

Based on the data obtained from bioinformatics and in vitro experiments, our subsequent investigation aims to determine whether CN maintains its therapeutic efficacy in an in vivo setting. An ONFH mouse model was then developed via intramuscular injection of MPS around the hip joint. CN was given at the same time as MPS and extendedly treated for another 3 weeks after the last MPS injection. The results of micro‐CT imaging and analysis showed deterioration of the subchondral trabeculae in the femoral head following MPS administration, as indicated by the diminished values of BV/TV, Tb.N, Tb.Sp and Tb.Th. Interestingly, treatment with CN significantly improved the values of BV/TV and Tb.Th (Figure 7B,C). Calcein labelling also corroborated the loss of trabeculae within the femoral head induced by MPS, further validating the successful establishment of the ONFH mouse model (Figure 7D). In contrast, CN treatment resulted in partial restoration of the subchondral trabecular bone. Histopathological staining revealed a significant presence of osteocyte lacunae within the femoral head, accompanied by the aggregation of OCs at the boundary between the lesion region and adjacent normal bone tissue. In contrast, CN treatment led to a partial recovery of the subchondral trabecular bone, a reduction in the number of empty osteocytic lacunae and an inhibition of OC activation (Figure 7E). These results affirmed CN's potential to alleviate or even prevent GCs‐induced ONFH in mice.

## 4 DISCUSSION

There has been a significant increase in the exploration of efficient and safe antioxidants derived from plants in recent years. CN, a naturally‐occurring alkaloid, has been recognized for its multifaceted biological activities, including the influence on the cellular redox system.^15^ In our previous research, we demonstrated a correlation between the suppression of ROS and the improvement of treating GCs‐induced ONFH.^36^ Therefore, in this study, from a translational aspect, we provide the first evidence suggesting CN's involvement in interfering with the differentiation and function of OCs and OBs under DEX condition, showing significant potential in treating ONFH. Furthermore, based on our bioinformatic and biological validations, this potential of CN is primarily exerted through the activation of Nrf2‐dependent antioxidant enzymes in OCs and OBs.

Many plants within the *Stephania* genus have a history of medicinal use. Since alkaloids display a range of pharmacological effects, including, but not limited to, anti‐inflammatory and antioxidant actions,^12^ CN is expected to be a promising option for ONFH treatment. As a result, we set out to explore the connection between CN's autochthonous targets and pathogenic genes of ONFH, utilizing approaches of network pharmacology and bioinformatics. Our target prediction of CN, supported by GO analysis, suggests it may potentially affect genes that participate in the metabolic process of ROS. This aligns with earlier studies demonstrating the efficacy of CN in treating various diseases by modulating ROS.^17^, ^18^ While the KEGG enrichment analysis does not directly indicate a correlation between crebanine and the oxidative stress, numerous KEGG‐demonstrated signalling pathways including but not limited to PI3K‐AKT signalling, calcium signalling and MAPK signalling, have close associations with the oxidative stress. The Keap1‐Nrf2 signalling, acknowledged as one of the classical antioxidant pathways, also interacts synergistically with those signalling pathways to regulate cellular survival, proliferation, metabolism and growth. For instance, the PI3K‐Akt signalling potentially enhances the stability and activity of Nrf2 by inhibiting GSK‐3β, thereby mitigating Nrf2 degradation.^37^, ^38^ In addition, CaMKII phosphorylation, prompted by TRPV1‐driven Ca^2+^ influx, facilitates Nrf2 activation directly.^39^ Also, the MAPK signalling can enhance Nrf2 activity by phosphorylating Nrf2, promoting its release from Keap1.^40^ We, therefore, assume that crebanine primarily targets the redox signalling pathway. Nrf2, a key transcription factor, regulates the expression of a host of antioxidant and detoxification enzymes. Its activation escalates the expression of ROS scavenging enzymes, thereby reducing the overall oxidative burden in bone cells.^35^ Nrf2 also involves in redox signalling through reactive cysteine thiols and contributes to the regulation of autophagy.^41^, ^42^ Specifically, within the ONFH model, the activation of the Nrf2 pathway has exhibited an ability to attenuate oxidative stress and partially recuperate apoptosis in BMSCs.^43^ Given the well‐documented links between these pathways and the differentiation and function of OCs and OBs,^31^, ^32^, ^33^ it is reasonable to hypothesize that CN may potentially impact OCs and OBs via by ROS regulation. Research, including ours, indicated that the pathological progression of ONFH correlates strongly with the disruption of bone remodelling, wherein GCs‐induced hyperactivation of OCs activity and impairment of OBs functionality via inciting oxidative stress ranks one of the prominent causes.^8^, ^36^, ^43^, ^44^ Therefore, given these insights drawn from the literature studies and our bioinformatic prediction, CN demonstrates a promising potential as a preventive or therapeutic agent for ONFH.

In the event of femoral head necrosis, disturbed trabecular architecture and bone loss is typically observed in the region of proximal femoral head.^30^ By carrying out steroid injections, a common method that employed to model ONFH in animals, our study found better osteometric indices (BV, BV/TV and Tb.Th) and histomorphometry result in the CN‐treated group over those of the MPS‐treated group. Further, we undertook cellular experiments to elucidate the specific mechanisms of CN's effects on ONFH. By focusing our investigation on the differentiation and function of OCs and OBs, our data displayed DEX at concentrations of 10^−8^ M obviously enhanced OC formation and at concentrations of 10^−5^ M suppressed OB differentiation, which aligns with previous researches.^34^, ^45^ In contrast, OC generation was significantly reduced after CN treatment, as well as the DEX‐stimulated formation of F‐actin rings and acidic vesicles. Notably, the formation of a sealing zone, constituted by F‐actin rings, and the generation of acidified compartments through acidic vesicles, are fundamental prerequisites for the bone resorption function.^46^ On the other aspect of bone remodelling,^47^ CN exhibited the ability to partially counteract the DEX‐induced impairment of osteogenic differentiation and function. Further traced back to the molecular level, CN downregulated the expression of Nfatc1, Traf6 and Ctsk in OCs, and upregulated the expression of Runx2, OPN and Col1α1 in OBs, subsequently dictating phenotype changes in cell behaviours.^46^, ^48^

Under pathological conditions like ONFH, the excessive accumulation of intracellular ROS significantly influences the differentiation and function of both OCs and OBs, demonstrating promotion of osteoclastogenesis and deterioration of osteogenesis.^7^, ^8^ GCs can suppress the expression levels of antioxidant enzymes, leading to an insufficient capability of the antioxidants and thereby pushing the ROS homeostasis into the pathological state.^49^ Our findings indicated that DEX exposure elevated the intracellular ROS level in BMMs, whereas the treatment of CN activated the Nrf2 expression and enhanced the antioxidant enzymes including Sod1, Sod2, Gr, Gpx and Cat, effectively curbing ROS formation, which aligns with previous studies.^28^, ^42^, ^50^ Notably, ROS contribute to osteoclastogenesis by functioning as intracellular signalling molecules subsequent to RANKL signalling.^51^, ^52^ In contrast, other reports stated high doses of DEX can cause oxidative stress which impairs the mineralization of OBs, while ROS scavengers can mitigate DEX‐induced OB damage.^25^, ^53^, ^54^ Similarly, our observations demonstrated that CN upregulated the Nrf2 expression in OPCs, in turn increasing the expression the antioxidant enzyme and alleviating the osteogenic damage by diminishing the DEX‐induced oxidative stress. Prior research indicates that alkaloids have the capacity to activate the Nrf2 pathway through various mechanisms, such as direct enhancement of Nrf2 synthesis and its nuclear translocation, or indirectly activating Nrf2 via the regulation of upstream pathways.^55^, ^56^, ^57^, ^58^ Combined with our results, it is our contention that CN indirectly stimulates Nrf2 expression by modulating Keap1. These findings suggest that rebalancing ROS may present a promising therapeutic approach for GCs‐induced ONFH. Future investigations could delve deeper into the mechanisms using advanced techniques.^59^

## 5 CONCLUSION

In conclusion, our study suggests that CN manifests therapeutic potential against GCs‐induced ONFH by reducing ROS in OCs and OBs, probably through the activation of the Nrf2 signalling pathway. These findings imply that CN may as a promising candidate for the prevention and treatment of ONFH.

## AUTHOR CONTRIBUTIONS

Shankun Dong: writing—original draft (equal), investigation (equal), data curation (equal), formal analysis (equal), validation (equal) and visualization (equal). Jianxun Ge: writing—original draft (equal), investigation (equal), data curation (equal), formal analysis (equal) and validation (equal). Qi Meng: investigation (equal) and software (equal). Tao Yuan: methodology (equal) and validation (equal). Yi Wang: methodology (equal) and validation (equal). Yi Li: funding acquisition (equal) and resources (equal). Qizhen Lu: investigation (equal) and resources (equal). Wenao Song: data curation (equal) and visualization (equal). Ziqing Li: writing—review and editing (equal), supervision (equal), project administration (equal) and funding acquisition (equal). Shui Sun: writing—review and editing (equal), supervision (equal), project administration (equal) and funding acquisition (equal).

## CONFLICT OF INTEREST STATEMENT

The authors declare no conflict of interest.

## Supporting information


Table S1.

Table S2.

Table S3.

## Data Availability

The datasets used and/or analysed during the current study are available from the corresponding author on reasonable request.
