# Supplementary material for: Crebanine mitigates glucocorticoid‐induced osteonecrosis of the femoral head by restoring bone remodelling homeostasis via attenuating oxidative stress
Source: J Cell Mol Med. 2024 Aug 28;28(16):e70044. doi: 10.1111/jcmm.70044 (PMC11358393; doi:10.1111/jcmm.70044)
Supplement: Supplementary file 1 — Table S1. [file JCMM-28-e70044-s001.docx]

Supplement Table 1: Primer sequences of RT-qPCR

| **Gene Symbol (GenBank Accession No.)** | **Primers** |
| --- | --- |
| ALP (NM_007431.3) | F: GCACCTGCCTTACCAACTCT |
|  | R: GTGGAGACGCCCATACCATC |
| RUNX2 (NM_001271631.1) | F: TCAAGGGAATAGAGGGGATGC |
|  | R: GGGAGGACAGAGGGAAACAAC |
| Col1α1 (NM_007742.4) | F: GACATGTTCAGCTTTGTGGACCTC |
|  | R: GGGACCCTTAGGCCATTGTGTA |
| Traf6 (NM_001303273.1) | F: AAAGCGAGAGATTCTTTCCCTG |
|  | R: ACTGGGGACAATTCACTAGAGC |
| NFATc1 (NM_001164112.1) | F: CCGTTGCTTCCAGAAAATAACA |
|  | R: TGTGGGATGTGAACTCGGAA |
| Ctsk (NM_007802.4) | F: CTTCCAATACGTGCAGCAGA |
|  | R: TCTTCAGGGCTTTCTCGTTC |
| Sod1 (NM_011434.2) | F: AACCAGTTGTGTTGTCAGGAC |
|  | R: CCACCATGTTTCTTAGAGTGAGG |
| Sod2 (NM_013671.3) | F: TGGACAAACCTGAGCCCTAAG |
|  | R: CCCAAAGTCACGCTTGATAGC |
| Cat (NM_009804.2) | F: GGAGTCTTCGTCCCGAGTCT |
|  | R: CGGTCTTGTAATGGAACTTGC |
| Gpx (NM_001329527.1) | F: AGTCCACCGTGTATGCCTTCT |
|  | R: GAGACGCGACATTCTCAATGA |
| Gr (NM_010344.4) | F: GCGTGAATGTTGGATGTGTACC |
|  | R: GTTGCATAGCCGTGGATAATTTC |
| GAPDH (NM_001289726.1) | F: ACTTTGTCAAGCTCATTTCC |
|  | R: TGCAGCGAACTTTATTGATG |
|  |  |
