# Supplementary material for: Crebanine mitigates glucocorticoid‐induced osteonecrosis of the femoral head by restoring bone remodelling homeostasis via attenuating oxidative stress
Source: J Cell Mol Med. 2024 Aug 28;28(16):e70044. doi: 10.1111/jcmm.70044 (PMC11358393; doi:10.1111/jcmm.70044)
Supplement: Supplementary file 2 — Table S2. [file JCMM-28-e70044-s003.docx]

Supplement Table 2: 100 potential genes of crebanine

| Target | Common name | Uniprot ID |
| --- | --- | --- |
| Alpha-1a adrenergic receptor | ADRA1A | P35348 |
| Serotonin 2b (5-HT2b) receptor | HTR2B | P41595 |
| Dopamine D5 receptor | DRD5 | P21918 |
| Alpha-2a adrenergic receptor | ADRA2A | P08913 |
| Serotonin 1b (5-HT1b) receptor | HTR1B | P28222 |
| Adrenergic receptor alpha-2 | ADRA2C | P18825 |
| Histamine H2 receptor | HRH2 | P25021 |
| Alpha-2b adrenergic receptor | ADRA2B | P18089 |
| Serotonin 1d (5-HT1d) receptor | HTR1D | P28221 |
| Dopamine D1 receptor | DRD1 | P21728 |
| Beta-1 adrenergic receptor | ADRB1 | P08588 |
| Dopamine D2 receptor | DRD2 | P14416 |
| Dopamine D4 receptor | DRD4 | P21917 |
| Alpha-1d adrenergic receptor | ADRA1D | P25100 |
| Serotonin 2a (5-HT2a) receptor | HTR2A | P28223 |
| Serotonin 2c (5-HT2c) receptor | HTR2C | P28335 |
| Serotonin transporter | SLC6A4 | P31645 |
| Alpha-1b adrenergic receptor | ADRA1B | P35368 |
| Mu opioid receptor | OPRM1 | P35372 |
| Dopamine D3 receptor | DRD3 | P35462 |
| Serotonin 7 (5-HT7) receptor | HTR7 | P34969 |
| Serotonin 6 (5-HT6) receptor | HTR6 | P50406 |
| Serotonin 5a (5-HT5a) receptor | HTR5A | P47898 |
| Dopamine transporter (by homology) | SLC6A3 | Q01959 |
| Translocator protein | TSPO | P30536 |
| Serotonin 1a (5-HT1a) receptor | HTR1A | P08908 |
| Neuronal acetylcholine receptor; alpha4/beta2 | CHRNA4 CHRNB2 | P43681 P17787 |
| Protein tyrosine phosphatase receptor type C-associated protein | PTPRCAP | Q14761 |
| HERG | KCNH2 | Q12809 |
| Vascular endothelial growth factor receptor 2 | KDR | P35968 |
| Steryl-sulfatase | STS | P08842 |
| Tyrosine 3-hydroxylase | TH | P07101 |
| c-Jun N-terminal kinase 1 | MAPK8 | P45983 |
| Dual specificity protein kinase TTK | TTK | P33981 |
| Glycogen synthase kinase-3 beta | GSK3B | P49841 |
| Protein kinase C alpha | PRKCA | P17252 |
| Anandamide amidohydrolase | FAAH | O00519 |
| Macrophage colony stimulating factor receptor | CSF1R | P07333 |
| Tyrosine-protein kinase JAK3 | JAK3 | P52333 |
| Tyrosine-protein kinase JAK1 | JAK1 | P23458 |
| Leucine-rich repeat serine/threonine-protein kinase 2 | LRRK2 | Q5S007 |
| MAP kinase p38 alpha | MAPK14 | Q16539 |
| Cyclin-dependent kinase 2/cyclin E1 | CCNE1 CDK2 | P24864 P24941 |
| Cyclin-dependent kinase 2/cyclin A | CDK2 CCNA1 CCNA2 | P24941 P78396 P20248 |
| Glycogen synthase kinase-3 alpha | GSK3A | P49840 |
| 7,8-dihydro-8-oxoguanine triphosphatase | NUDT1 | P36639 |
| Bromodomain adjacent to zinc finger domain protein 2B | BAZ2B | Q9UIF8 |
| Bromodomain adjacent to zinc finger domain protein 2A | BAZ2A | Q9UIF9 |
| Casein kinase II alpha | CSNK2A1 | P68400 |
| Casein kinase II alpha (prime) | CSNK2A2 | P19784 |
| Tyrosine-protein kinase JAK2 | JAK2 | O60674 |
| Quinone reductase 2 | NQO2 | P16083 |
| ALK tyrosine kinase receptor | ALK | Q9UM73 |
| Phosphodiesterase 5A | PDE5A | O76074 |
| Cyclin-dependent kinase 2 | CDK2 | P24941 |
| Nitric oxide synthase, inducible | NOS2 | P35228 |
| MAP kinase signal-integrating kinase 2 | MKNK2 | Q9HBH9 |
| Epidermal growth factor receptor erbB1 | EGFR | P00533 |
| MAP kinase ERK2 | MAPK1 | P28482 |
| PI3-kinase p110-delta subunit | PIK3CD | O00329 |
| NAD-dependent deacetylase sirtuin 1 | SIRT1 | Q96EB6 |
| Hepatocyte growth factor receptor | MET | P08581 |
| Dual specificty protein kinase CLK1 | CLK1 | P49759 |
| ATP-binding cassette sub-family G member 2 | ABCG2 | Q9UNQ0 |
| Vascular endothelial growth factor receptor 1 | FLT1 | P17948 |
| Platelet-derived growth factor receptor beta | PDGFRB | P09619 |
| Stem cell growth factor receptor | KIT | P10721 |
| Vascular endothelial growth factor receptor 3 | FLT4 | P35916 |
| Insulin-like growth factor I receptor | IGF1R | P08069 |
| Platelet-derived growth factor receptor alpha | PDGFRA | P16234 |
| Long-chain fatty acid transport protein 1 | SLC27A1 | Q6PCB7 |
| Tyrosine-protein kinase TIE-2 | TEK | Q02763 |
| Phosphodiesterase 10A | PDE10A | Q9Y233 |
| Protein tyrosine kinase 2 beta | PTK2B | Q14289 |
| Cyclin-dependent kinase 5/CDK5 activator 1 | CDK5R1 CDK5 | Q15078 Q00535 |
| Kinesin-1 heavy chain/ Tyrosine-protein kinase receptor RET | RET | P07949 |
| Serine/threonine-protein kinase PIM1 | PIM1 | P11309 |
| Dual-specificity tyrosine-phosphorylation regulated kinase 1A (by homology) | DYRK1A | Q13627 |
| C-C chemokine receptor type 1 | CCR1 | P32246 |
| Tyrosine-protein kinase SRC | SRC | P12931 |
| Dual specificity protein kinase CLK3 (by homology) | CLK3 | P49761 |
| Dual-specificity tyrosine-phosphorylation regulated kinase 2 | DYRK2 | Q92630 |
| Glutaminyl-peptide cyclotransferase | QPCT | Q16769 |
| Cytochrome P450 11B1 | CYP11B1 | P15538 |
| Cytochrome P450 19A1 | CYP19A1 | P11511 |
| Cytochrome P450 11B2 | CYP11B2 | P19099 |
| Nerve growth factor receptor Trk-A | NTRK1 | P04629 |
| Nitric-oxide synthase, brain | NOS1 | P29475 |
| Heat shock protein HSP 90-alpha | HSP90AA1 | P07900 |
| Serine/threonine-protein kinase Chk1 | CHEK1 | O14757 |
| Nitric-oxide synthase, endothelial | NOS3 | P29474 |
| Phosphatidylinositol-5-phosphate 4-kinase type-2 gamma | PIP4K2C | Q8TBX8 |
| Tyrosine-protein kinase LCK (by homology) | LCK | P06239 |
| Phosphodiesterase 4B | PDE4B | Q07343 |
| Serine/threonine-protein kinase B-raf | BRAF | P15056 |
| Tyrosine-protein kinase SYK | SYK | P43405 |
| Tyrosine-protein kinase Lyn | LYN | P07948 |
| Tyrosine-protein kinase TXK | TXK | P42681 |
| Lymphocyte differentiation antigen CD38 | CD38 | P28907 |
| Tyrosine-protein kinase BTK | BTK | Q06187 |
