# Supplementary material for: Crebanine mitigates glucocorticoid‐induced osteonecrosis of the femoral head by restoring bone remodelling homeostasis via attenuating oxidative stress
Source: J Cell Mol Med. 2024 Aug 28;28(16):e70044. doi: 10.1111/jcmm.70044 (PMC11358393; doi:10.1111/jcmm.70044)
Supplement: Supplementary file 3 — Table S3. [file JCMM-28-e70044-s002.docx]

Supplement Table 3: 3472 human genes linked to ONFH

| Gene Symbol | Description | Uniprot ID |
| --- | --- | --- |
| COL2A1 | Collagen Type II Alpha 1 Chain | P02458 |
| TNF | Tumor Necrosis Factor | P01375 |
| TP53 | Tumor Protein P53 | P04637 |
| TRPV4 | Transient Receptor Potential Cation Channel Subfamily V Member 4 | Q9HBA0 |
| TNFRSF1A | TNF Receptor Superfamily Member 1A | P19438 |
| IL6 | Interleukin 6 | P05231 |
| EGFR | Epidermal Growth Factor Receptor | P00533 |
| TNFRSF1B | TNF Receptor Superfamily Member 1B | P20333 |
| TNFSF10 | TNF Superfamily Member 10 | P50591 |
| CDKN2A | Cyclin Dependent Kinase Inhibitor 2A | Q8N726 |
| IL1B | Interleukin 1 Beta | P01584 |
| TNFSF11 | TNF Superfamily Member 11 | O14788 |
| IL10 | Interleukin 10 | P22301 |
| FGFR3 | Fibroblast Growth Factor Receptor 3 | P22607 |
| PTEN | Phosphatase And Tensin Homolog | P60484 |
| CTNNB1 | Catenin Beta 1 | P35222 |
| SMAD4 | SMAD Family Member 4 | Q13485 |
| FAS | Fas Cell Surface Death Receptor | P25445 |
| LTA | Lymphotoxin Alpha | P01374 |
| MAPK1 | Mitogen-Activated Protein Kinase 1 | P28482 |
| TNFRSF11B | TNF Receptor Superfamily Member 11b | O00300 |
| CXCL8 | C-X-C Motif Chemokine Ligand 8 | P10145 |
| CREBBP | CREB Binding Protein | Q92793 |
| CASP8 | Caspase 8 | Q14790 |
| AKT1 | AKT Serine/Threonine Kinase 1 | P31749 |
| IFNG | Interferon Gamma | P01579 |
| VEGFA | Vascular Endothelial Growth Factor A | P15692 |
| FGFR2 | Fibroblast Growth Factor Receptor 2 | P21802 |
| CRP | C-Reactive Protein | P02741 |
| TGFB1 | Transforming Growth Factor Beta 1 | P01137 |
| EP300 | E1A Binding Protein P300 | Q09472 |
| TNFRSF10A | TNF Receptor Superfamily Member 10a | O00220 |
| H19 | H19 Imprinted Maternally Expressed Transcript |  |
| MIR21 | MicroRNA 21 |  |
| ERBB2 | Erb-B2 Receptor Tyrosine Kinase 2 | P04626 |
| MMP9 | Matrix Metallopeptidase 9 | P14780 |
| CASP3 | Caspase 3 | P42574 |
| B2M | Beta-2-Microglobulin | P61769 |
| TNFRSF11A | TNF Receptor Superfamily Member 11a | Q9Y6Q6 |
| NFE2L2 | NFE2 Like BZIP Transcription Factor 2 | Q16236 |
| GNPTAB | N-Acetylglucosamine-1-Phosphate Transferase Subunits Alpha And Beta | Q3T906 |
| COL1A1 | Collagen Type I Alpha 1 Chain | P02452 |
| COMP | Cartilage Oligomeric Matrix Protein | P49747 |
| IL1A | Interleukin 1 Alpha | P01583 |
| NOTCH1 | Notch Receptor 1 | P46531 |
| FBN1 | Fibrillin 1 | P35555 |
| TRAF2 | TNF Receptor Associated Factor 2 | Q12933 |
| MAP2K1 | Mitogen-Activated Protein Kinase Kinase 1 | Q02750 |
| MIR210 | MicroRNA 210 |  |
| TNFSF13B | TNF Superfamily Member 13b | Q9Y275 |
| CCL2 | C-C Motif Chemokine Ligand 2 | P13500 |
| TNFAIP3 | TNF Alpha Induced Protein 3 | P21580 |
| PTGS2 | Prostaglandin-Endoperoxide Synthase 2 | P35354 |
| RHOA | Ras Homolog Family Member A | P61586 |
| SMAD3 | SMAD Family Member 3 | P84022 |
| MIR221 | MicroRNA 221 |  |
| TNFSF12 | TNF Superfamily Member 12 | O43508 |
| CD40LG | CD40 Ligand | P29965 |
| RIPK1 | Receptor Interacting Serine/Threonine Kinase 1 | Q13546 |
| CSF2 | Colony Stimulating Factor 2 | P04141 |
| TLR4 | Toll Like Receptor 4 | O00206 |
| BCL2 | BCL2 Apoptosis Regulator | P10415 |
| LOX | Lysyl Oxidase | P28300 |
| RAC1 | Rac Family Small GTPase 1 | P63000 |
| NFKB1 | Nuclear Factor Kappa B Subunit 1 | P19838 |
| MIR155 | MicroRNA 155 |  |
| MEG3 | Maternally Expressed 3 |  |
| TGFBR2 | Transforming Growth Factor Beta Receptor 2 | P37173 |
| CD40 | CD40 Molecule | P25942 |
| CCND1 | Cyclin D1 | P24385 |
| HLA-DRB1 | Major Histocompatibility Complex, Class II, DR Beta 1 | P01911 |
| ACE | Angiotensin I Converting Enzyme | P12821 |
| TRADD | TNFRSF1A Associated Via Death Domain | Q15628 |
| MIR146B | MicroRNA 146b |  |
| ESR1 | Estrogen Receptor 1 | P03372 |
| ADAM17 | ADAM Metallopeptidase Domain 17 | P78536 |
| IGF1 | Insulin Like Growth Factor 1 | P05019 |
| SERPINE1 | Serpin Family E Member 1 | P05121 |
| MIR195 | MicroRNA 195 |  |
| IL18 | Interleukin 18 | Q14116 |
| ICAM1 | Intercellular Adhesion Molecule 1 | P05362 |
| TNFSF4 | TNF Superfamily Member 4 | P23510 |
| F2 | Coagulation Factor II, Thrombin | P00734 |
| MAPK8 | Mitogen-Activated Protein Kinase 8 | P45983 |
| U2AF1 | U2 Small Nuclear RNA Auxiliary Factor 1 | Q01081 |
| SDHD | Succinate Dehydrogenase Complex Subunit D | O14521 |
| TERT | Telomerase Reverse Transcriptase | O14746 |
| BMP2 | Bone Morphogenetic Protein 2 | P12643 |
| MMP3 | Matrix Metallopeptidase 3 | P08254 |
| MMP1 | Matrix Metallopeptidase 1 | P03956 |
| SELE | Selectin E | P16581 |
| SDHB | Succinate Dehydrogenase Complex Iron Sulfur Subunit B | P21912 |
| MATN3 | Matrilin 3 | O15232 |
| FGFR1 | Fibroblast Growth Factor Receptor 1 | P11362 |
| FADD | Fas Associated Via Death Domain | Q13158 |
| BRCA2 | BRCA2 DNA Repair Associated | P51587 |
| F5 | Coagulation Factor V | P12259 |
| LEP | Leptin | P41159 |
| ADIPOQ | Adiponectin, C1Q And Collagen Domain Containing | Q15848 |
| STAT3 | Signal Transducer And Activator Of Transcription 3 | P40763 |
| RUNX2 | RUNX Family Transcription Factor 2 | Q13950 |
| IL4 | Interleukin 4 | P05112 |
| MMP13 | Matrix Metallopeptidase 13 | P45452 |
| MIR122 | MicroRNA 122 |  |
| MYLK | Myosin Light Chain Kinase | Q15746 |
| F3 | Coagulation Factor III, Tissue Factor | P13726 |
| GNAS | GNAS Complex Locus | P84996 |
| UFSP2 | UFM1 Specific Peptidase 2 | Q9NUQ7 |
| ALB | Albumin | P02768 |
| HIF1A | Hypoxia Inducible Factor 1 Subunit Alpha | Q16665 |
| IL2 | Interleukin 2 | P60568 |
| MAP2K2 | Mitogen-Activated Protein Kinase Kinase 2 | P36507 |
| MIR214 | MicroRNA 214 |  |
| CYCS | Cytochrome C, Somatic | P99999 |
| FGF2 | Fibroblast Growth Factor 2 | P09038 |
| MIR142 | MicroRNA 142 |  |
| ACAN | Aggrecan | P16112 |
| IL1RN | Interleukin 1 Receptor Antagonist | P18510 |
| MIR31 | MicroRNA 31 |  |
| BRCA1 | BRCA1 DNA Repair Associated | P38398 |
| MMP2 | Matrix Metallopeptidase 2 | P08253 |
| RET | Ret Proto-Oncogene | P07949 |
| CDKN1A | Cyclin Dependent Kinase Inhibitor 1A | P38936 |
| MIR373 | MicroRNA 373 |  |
| INS | Insulin | P01308 |
| GAS5 | Growth Arrest Specific 5 |  |
| HOTAIR | HOX Transcript Antisense RNA |  |
| TGFBR1 | Transforming Growth Factor Beta Receptor 1 | P36897 |
| ADAR | Adenosine Deaminase RNA Specific | P55265 |
| SPP1 | Secreted Phosphoprotein 1 | P10451 |
| TGFB2 | Transforming Growth Factor Beta 2 | P61812 |
| SLC2A1 | Solute Carrier Family 2 Member 1 | P11166 |
| TNFRSF9 | TNF Receptor Superfamily Member 9 | Q07011 |
| MIRLET7D | MicroRNA Let-7d |  |
| EGF | Epidermal Growth Factor | P01133 |
| JUN | Jun Proto-Oncogene, AP-1 Transcription Factor Subunit | P05412 |
| CSF3 | Colony Stimulating Factor 3 | P09919 |
| SOX9 | SRY-Box Transcription Factor 9 | P48436 |
| XIAP | X-Linked Inhibitor Of Apoptosis | P98170 |
| FLNA | Filamin A | P21333 |
| NOS2 | Nitric Oxide Synthase 2 | P35228 |
| NFKBIA | NFKB Inhibitor Alpha | P25963 |
| BGLAP | Bone Gamma-Carboxyglutamate Protein | P02818 |
| VCAM1 | Vascular Cell Adhesion Molecule 1 | P19320 |
| PARP1 | Poly(ADP-Ribose) Polymerase 1 | P09874 |
| MTHFR | Methylenetetrahydrofolate Reductase | P42898 |
| MIR125A | MicroRNA 125a |  |
| MYH11 | Myosin Heavy Chain 11 | P35749 |
| WNT7A | Wnt Family Member 7A | O00755 |
| COL1A2 | Collagen Type I Alpha 2 Chain | P08123 |
| MIR148A | MicroRNA 148a |  |
| IGFBP3 | Insulin Like Growth Factor Binding Protein 3 | P17936 |
| BAX | BCL2 Associated X, Apoptosis Regulator | Q07812 |
| FZD2 | Frizzled Class Receptor 2 | Q14332 |
| FN1 | Fibronectin 1 | P02751 |
| IFNA1 | Interferon Alpha 1 | P01562 |
| EDN1 | Endothelin 1 | P05305 |
| IL1R1 | Interleukin 1 Receptor Type 1 | P14778 |
| TRAF6 | TNF Receptor Associated Factor 6 | Q9Y4K3 |
| MIR19A | MicroRNA 19a |  |
| WNT5A | Wnt Family Member 5A | P41221 |
| MYC | MYC Proto-Oncogene, BHLH Transcription Factor | P01106 |
| APOE | Apolipoprotein E | P02649 |
| HLA-B | Major Histocompatibility Complex, Class I, B | P01889 |
| CERNA3 | Competing Endogenous LncRNA 3 For MiR-645 |  |
| PPARG | Peroxisome Proliferator Activated Receptor Gamma | P37231 |
| MIR98 | MicroRNA 98 |  |
| ANXA5 | Annexin A5 | P08758 |
| IL17A | Interleukin 17A | Q16552 |
| MIR222 | MicroRNA 222 |  |
| HGF | Hepatocyte Growth Factor | P14210 |
| RB1 | RB Transcriptional Corepressor 1 | P06400 |
| PRKD1 | Protein Kinase D1 | Q15139 |
| MAPK14 | Mitogen-Activated Protein Kinase 14 | Q16539 |
| BGN | Biglycan | P21810 |
| MIR494 | MicroRNA 494 |  |
| GLI3 | GLI Family Zinc Finger 3 | P10071 |
| GSTM1 | Glutathione S-Transferase Mu 1 | P09488 |
| TYMS | Thymidylate Synthetase | P04818 |
| LMNA | Lamin A/C | P02545 |
| DYM | Dymeclin | Q7RTS9 |
| VDR | Vitamin D Receptor | P11473 |
| STAT1 | Signal Transducer And Activator Of Transcription 1 | P42224 |
| CSF1 | Colony Stimulating Factor 1 | P09603 |
| TGFB3 | Transforming Growth Factor Beta 3 | P10600 |
| SMAD6 | SMAD Family Member 6 | O43541 |
| COL9A1 | Collagen Type IX Alpha 1 Chain | P20849 |
| EGFR-AS1 | EGFR Antisense RNA 1 |  |
| SLC26A2 | Solute Carrier Family 26 Member 2 | P50443 |
| IHH | Indian Hedgehog Signaling Molecule | Q14623 |
| IGF1R | Insulin Like Growth Factor 1 Receptor | P08069 |
| SLC2A10 | Solute Carrier Family 2 Member 10 | O95528 |
| MIR34A | MicroRNA 34a |  |
| MIR148B | MicroRNA 148b |  |
| RNF217-AS1 | RNF217 Antisense RNA 1 (Head To Head) |  |
| CASP9 | Caspase 9 | P55211 |
| NOS3 | Nitric Oxide Synthase 3 | P29474 |
| BCL2L1 | BCL2 Like 1 | Q07817 |
| SHH | Sonic Hedgehog Signaling Molecule | Q15465 |
| HMGB1 | High Mobility Group Box 1 | P09429 |
| COL9A2 | Collagen Type IX Alpha 2 Chain | Q14055 |
| MIR30B | MicroRNA 30b |  |
| ABCB1 | ATP Binding Cassette Subfamily B Member 1 | P08183 |
| MIR17 | MicroRNA 17 |  |
| FANCG | FA Complementation Group G | O15287 |
| RNU4ATAC | RNA, U4atac Small Nuclear |  |
| MALAT1 | Metastasis Associated Lung Adenocarcinoma Transcript 1 |  |
| CD44 | CD44 Molecule (Indian Blood Group) | P16070 |
| CHUK | Component Of Inhibitor Of Nuclear Factor Kappa B Kinase Complex | O15111 |
| TWIST1 | Twist Family BHLH Transcription Factor 1 | Q15672 |
| BDNF-AS | BDNF Antisense RNA |  |
| TIMP1 | TIMP Metallopeptidase Inhibitor 1 | P01033 |
| TGFA | Transforming Growth Factor Alpha | P01135 |
| MTOR | Mechanistic Target Of Rapamycin Kinase | P42345 |
| CDH1 | Cadherin 1 | P12830 |
| TRAF1 | TNF Receptor Associated Factor 1 | Q13077 |
| SELP | Selectin P | P16109 |
| SRC | SRC Proto-Oncogene, Non-Receptor Tyrosine Kinase | P12931 |
| ELN | Elastin | P15502 |
| MIR22 | MicroRNA 22 |  |
| LRP5 | LDL Receptor Related Protein 5 | O75197 |
| HAND2-AS1 | HAND2 Antisense RNA 1 |  |
| RELA | RELA Proto-Oncogene, NF-KB Subunit | Q04206 |
| KDR | Kinase Insert Domain Receptor | P35968 |
| CASP1 | Caspase 1 | P29466 |
| HMOX1 | Heme Oxygenase 1 | P09601 |
| GBA1 | Glucosylceramidase Beta 1 | P04062 |
| IGF2 | Insulin Like Growth Factor 2 | P01344 |
| NTRK1 | Neurotrophic Receptor Tyrosine Kinase 1 | P04629 |
| GSTP1 | Glutathione S-Transferase Pi 1 | P09211 |
| CD4 | CD4 Molecule | P01730 |
| NF1 | Neurofibromin 1 | P21359 |
| NEK9 | NIMA Related Kinase 9 | Q8TD19 |
| IKBKB | Inhibitor Of Nuclear Factor Kappa B Kinase Subunit Beta | O14920 |
| CHEK2 | Checkpoint Kinase 2 | O96017 |
| BMP6 | Bone Morphogenetic Protein 6 | P22004 |
| EZH2 | Enhancer Of Zeste 2 Polycomb Repressive Complex 2 Subunit | Q15910 |
| DVL1 | Dishevelled Segment Polarity Protein 1 | O14640 |
| TLR2 | Toll Like Receptor 2 | O60603 |
| FOXE3 | Forkhead Box E3 | Q13461 |
| CD36 | CD36 Molecule | P16671 |
| ERCC6 | ERCC Excision Repair 6, Chromatin Remodeling Factor | Q03468 |
| CENPJ | Centromere Protein J | Q9HC77 |
| MIR146A | MicroRNA 146a |  |
| CDK6 | Cyclin Dependent Kinase 6 | Q00534 |
| COL9A3 | Collagen Type IX Alpha 3 Chain | Q14050 |
| NT5E | 5'-Nucleotidase Ecto | P21589 |
| CALCA | Calcitonin Related Polypeptide Alpha | P06881 |
| LTBP3 | Latent Transforming Growth Factor Beta Binding Protein 3 | Q9NS15 |
| SMAD2 | SMAD Family Member 2 | Q15796 |
| COL3A1 | Collagen Type III Alpha 1 Chain | P02461 |
| LBR | Lamin B Receptor | Q14739 |
| MET | MET Proto-Oncogene, Receptor Tyrosine Kinase | P08581 |
| SOD1 | Superoxide Dismutase 1 | P00441 |
| TINF2 | TERF1 Interacting Nuclear Factor 2 | Q9BSI4 |
| MMP14 | Matrix Metallopeptidase 14 | P50281 |
| MB | Myoglobin | P02144 |
| DVL3 | Dishevelled Segment Polarity Protein 3 | Q92997 |
| GDF5 | Growth Differentiation Factor 5 | P43026 |
| DKK1 | Dickkopf WNT Signaling Pathway Inhibitor 1 | O94907 |
| MECP2 | Methyl-CpG Binding Protein 2 | P51608 |
| MIR34C | MicroRNA 34c |  |
| MEFV | MEFV Innate Immunity Regulator, Pyrin | O15553 |
| ACTA2 | Actin Alpha 2, Smooth Muscle | P62736 |
| NGFR | Nerve Growth Factor Receptor | P08138 |
| POC1A | POC1 Centriolar Protein A | Q8NBT0 |
| ATRX | ATRX Chromatin Remodeler | P46100 |
| LUCAT1 | Lung Cancer Associated Transcript 1 |  |
| CD27 | CD27 Molecule | P26842 |
| IL2RA | Interleukin 2 Receptor Subunit Alpha | P01589 |
| HSPG2 | Heparan Sulfate Proteoglycan 2 | P98160 |
| FOS | Fos Proto-Oncogene, AP-1 Transcription Factor Subunit | P01100 |
| TIMP3 | TIMP Metallopeptidase Inhibitor 3 | P35625 |
| MIR628 | MicroRNA 628 |  |
| IFNB1 | Interferon Beta 1 | P01574 |
| MIR181A1 | MicroRNA 181a-1 |  |
| ACTB | Actin Beta | P60709 |
| GALNS | Galactosamine (N-Acetyl)-6-Sulfatase | P34059 |
| SLC39A13 | Solute Carrier Family 39 Member 13 | Q96H72 |
| CXCL12 | C-X-C Motif Chemokine Ligand 12 | P48061 |
| APOB | Apolipoprotein B | P04114 |
| MDM2 | MDM2 Proto-Oncogene | Q00987 |
| XYLT1 | Xylosyltransferase 1 | Q86Y38 |
| PIK3C2A | Phosphatidylinositol-4-Phosphate 3-Kinase Catalytic Subunit Type 2 Alpha | O00443 |
| CXCR4 | C-X-C Motif Chemokine Receptor 4 | P61073 |
| HBB | Hemoglobin Subunit Beta | P68871 |
| EXT1 | Exostosin Glycosyltransferase 1 | Q16394 |
| SETD2 | SET Domain Containing 2, Histone Lysine Methyltransferase | Q9BYW2 |
| TNFAIP2 | TNF Alpha Induced Protein 2 | Q03169 |
| TERC | Telomerase RNA Component |  |
| APOA1 | Apolipoprotein A1 | P02647 |
| THBD | Thrombomodulin | P07204 |
| NPPA | Natriuretic Peptide A | P01160 |
| VWF | Von Willebrand Factor | P04275 |
| FGF8 | Fibroblast Growth Factor 8 | P55075 |
| VIM | Vimentin | P08670 |
| SBDS | SBDS Ribosome Maturation Factor | Q9Y3A5 |
| CDKN2B-AS1 | CDKN2B Antisense RNA 1 |  |
| PCNT | Pericentrin | O95613 |
| PDCD1 | Programmed Cell Death 1 | Q15116 |
| FBN2 | Fibrillin 2 | P35556 |
| PSMC3 | Proteasome 26S Subunit, ATPase 3 | P17980 |
| SOD2 | Superoxide Dismutase 2 | P04179 |
| CDK4 | Cyclin Dependent Kinase 4 | P11802 |
| ACTA1 | Actin Alpha 1, Skeletal Muscle | P68133 |
| PTCH1 | Patched 1 | Q13635 |
| TIMP2 | TIMP Metallopeptidase Inhibitor 2 | P16035 |
| RINT1 | RAD50 Interactor 1 | Q6NUQ1 |
| CEP152 | Centrosomal Protein 152 | O94986 |
| PRKG1 | Protein Kinase CGMP-Dependent 1 | Q13976 |
| FLNB | Filamin B | O75369 |
| CANT1 | Calcium Activated Nucleotidase 1 | Q8WVQ1 |
| JAK2 | Janus Kinase 2 | O60674 |
| REN | Renin | P00797 |
| PVT1 | Pvt1 Oncogene |  |
| PTH1R | Parathyroid Hormone 1 Receptor | Q03431 |
| POR | Cytochrome P450 Oxidoreductase | P16435 |
| TREX1 | Three Prime Repair Exonuclease 1 | Q9NSU2 |
| IKBKG | Inhibitor Of Nuclear Factor Kappa B Kinase Regulatory Subunit Gamma | Q9Y6K9 |
| MIR199A1 | MicroRNA 199a-1 |  |
| GSTT1 | Glutathione S-Transferase Theta 1 | P30711 |
| ELANE | Elastase, Neutrophil Expressed | P08246 |
| CFTR | CF Transmembrane Conductance Regulator | P13569 |
| CDKN1B | Cyclin Dependent Kinase Inhibitor 1B | P46527 |
| KRT18 | Keratin 18 | P05783 |
| RBBP8 | RB Binding Protein 8, Endonuclease | Q99708 |
| KIF22 | Kinesin Family Member 22 | Q14807 |
| PTHLH | Parathyroid Hormone Like Hormone | P12272 |
| CAT | Catalase | P04040 |
| PLAT | Plasminogen Activator, Tissue Type | P00750 |
| NPR2 | Natriuretic Peptide Receptor 2 | P20594 |
| FANCA | FA Complementation Group A | O15360 |
| TBX15 | T-Box Transcription Factor 15 | Q96SF7 |
| KIT | KIT Proto-Oncogene, Receptor Tyrosine Kinase | P10721 |
| LINC01672 | Long Intergenic Non-Protein Coding RNA 1672 |  |
| MAPK3 | Mitogen-Activated Protein Kinase 3 | P27361 |
| CYP1A1 | Cytochrome P450 Family 1 Subfamily A Member 1 | P04798 |
| GPT | Glutamic--Pyruvic Transaminase | P24298 |
| SOD2-OT1 | SOD2 Overlapping Transcript 1 |  |
| TMX2-CTNND1 | TMX2-CTNND1 Readthrough (NMD Candidate) |  |
| ACP5 | Acid Phosphatase 5, Tartrate Resistant | P13686 |
| MIR143 | MicroRNA 143 |  |
| IDUA | Alpha-L-Iduronidase | P35475 |
| TRPS1 | Transcriptional Repressor GATA Binding 1 | Q9UHF7 |
| SERPINC1 | Serpin Family C Member 1 | P01008 |
| POLG | DNA Polymerase Gamma, Catalytic Subunit | P54098 |
| NIPBL | NIPBL Cohesin Loading Factor | Q6KC79 |
| PIK3CG | Phosphatidylinositol-4,5-Bisphosphate 3-Kinase Catalytic Subunit Gamma | P48736 |
| EPO | Erythropoietin | P01588 |
| GFAP | Glial Fibrillary Acidic Protein | P14136 |
| DNMT3B | DNA Methyltransferase 3 Beta | Q9UBC3 |
| CTSK | Cathepsin K | P43235 |
| APOH | Apolipoprotein H | P02749 |
| ENPP1 | Ectonucleotide Pyrophosphatase/Phosphodiesterase 1 | P22413 |
| NAGLU | N-Acetyl-Alpha-Glucosaminidase | P54802 |
| PDGFRB | Platelet Derived Growth Factor Receptor Beta | P09619 |
| BDNF | Brain Derived Neurotrophic Factor | P23560 |
| LMX1B | LIM Homeobox Transcription Factor 1 Beta | O60663 |
| COL10A1 | Collagen Type X Alpha 1 Chain | Q03692 |
| NGF | Nerve Growth Factor | P01138 |
| COL11A2 | Collagen Type XI Alpha 2 Chain | P13942 |
| GHRL | Ghrelin And Obestatin Prepropeptide | Q9UBU3 |
| IL1RAPL2 | Interleukin 1 Receptor Accessory Protein Like 2 | Q9NP60 |
| RMRP | RNA Component Of Mitochondrial RNA Processing Endoribonuclease |  |
| RTEL1 | Regulator Of Telomere Elongation Helicase 1 | Q9NZ71 |
| GAPDH | Glyceraldehyde-3-Phosphate Dehydrogenase | P04406 |
| MSX2 | Msh Homeobox 2 | P35548 |
| MIR145 | MicroRNA 145 |  |
| ADAMTSL2 | ADAMTS Like 2 | Q86TH1 |
| CCR7 | C-C Motif Chemokine Receptor 7 | P32248 |
| MIR483 | MicroRNA 483 |  |
| GJA1 | Gap Junction Protein Alpha 1 | P17302 |
| TNFAIP8 | TNF Alpha Induced Protein 8 | O95379 |
| SMARCAL1 | SWI/SNF Related, Matrix Associated, Actin Dependent Regulator Of Chromatin, Subfamily A Like 1 | Q9NZC9 |
| GLB1 | Galactosidase Beta 1 | P16278 |
| IFNGR1 | Interferon Gamma Receptor 1 | P15260 |
| EPHB4 | EPH Receptor B4 | P54760 |
| CCL5 | C-C Motif Chemokine Ligand 5 | P13501 |
| MIR100 | MicroRNA 100 |  |
| PALB2 | Partner And Localizer Of BRCA2 | Q86YC2 |
| ENG | Endoglin | P17813 |
| CTC1 | CST Telomere Replication Complex Component 1 | Q2NKJ3 |
| JAG1 | Jagged Canonical Notch Ligand 1 | P78504 |
| XIST | X Inactive Specific Transcript |  |
| ATRIP | ATR Interacting Protein | Q8WXE1 |
| IL6R | Interleukin 6 Receptor | P08887 |
| POMC | Proopiomelanocortin | P01189 |
| IL15 | Interleukin 15 | P40933 |
| TONSL | Tonsoku Like, DNA Repair Protein | Q96HA7 |
| RYR1 | Ryanodine Receptor 1 | P21817 |
| MIR223 | MicroRNA 223 |  |
| TTN | Titin | Q8WZ42 |
| VEGFC | Vascular Endothelial Growth Factor C | P49767 |
| DKC1 | Dyskerin Pseudouridine Synthase 1 | O60832 |
| MIR10B | MicroRNA 10b |  |
| IL12B | Interleukin 12B | P29460 |
| PTH | Parathyroid Hormone | P01270 |
| E2F1 | E2F Transcription Factor 1 | Q01094 |
| RETN | Resistin | Q9HD89 |
| COL11A1 | Collagen Type XI Alpha 1 Chain | P12107 |
| SOST | Sclerostin | Q9BQB4 |
| IL3 | Interleukin 3 | P08700 |
| MIR25 | MicroRNA 25 |  |
| IL23R | Interleukin 23 Receptor | Q5VWK5 |
| APC | APC Regulator Of WNT Signaling Pathway | P25054 |
| BMP7 | Bone Morphogenetic Protein 7 | P18075 |
| EDNRA | Endothelin Receptor Type A | P25101 |
| TCIRG1 | T Cell Immune Regulator 1, ATPase H+ Transporting V0 Subunit A3 | Q13488 |
| SP1 | Sp1 Transcription Factor | P08047 |
| BMP4 | Bone Morphogenetic Protein 4 | P12644 |
| PLOD1 | Procollagen-Lysine,2-Oxoglutarate 5-Dioxygenase 1 | Q02809 |
| CLCN7 | Chloride Voltage-Gated Channel 7 | P51798 |
| MIR127 | MicroRNA 127 |  |
| IFIH1 | Interferon Induced With Helicase C Domain 1 | Q9BYX4 |
| IBSP | Integrin Binding Sialoprotein | P21815 |
| ALDH2 | Aldehyde Dehydrogenase 2 Family Member | P05091 |
| ATP7A | ATPase Copper Transporting Alpha | Q04656 |
| ADAMTS2 | ADAM Metallopeptidase With Thrombospondin Type 1 Motif 2 | O95450 |
| LGALS3 | Galectin 3 | P17931 |
| KITLG | KIT Ligand | P21583 |
| RAB3GAP2 | RAB3 GTPase Activating Non-Catalytic Protein Subunit 2 | Q9H2M9 |
| CREB1 | CAMP Responsive Element Binding Protein 1 | P16220 |
| PCNA | Proliferating Cell Nuclear Antigen | P12004 |
| TRAIP | TRAF Interacting Protein | Q9BWF2 |
| CYP1B1 | Cytochrome P450 Family 1 Subfamily B Member 1 | Q16678 |
| WNT1 | Wnt Family Member 1 | P04628 |
| CDC6 | Cell Division Cycle 6 | Q99741 |
| CXCL10 | C-X-C Motif Chemokine Ligand 10 | P02778 |
| CTSD | Cathepsin D | P07339 |
| MIR30A | MicroRNA 30a |  |
| POSTN | Periostin | Q15063 |
| NSD1 | Nuclear Receptor Binding SET Domain Protein 1 | Q96L73 |
| SMAD5-AS1 | SMAD5 Antisense RNA 1 | Q9Y6J3 |
| NLRP3 | NLR Family Pyrin Domain Containing 3 | Q96P20 |
| LINC-ROR | Long Intergenic Non-Protein Coding RNA, Regulator Of Reprogramming |  |
| COL5A1 | Collagen Type V Alpha 1 Chain | P20908 |
| COL4A1 | Collagen Type IV Alpha 1 Chain | P02462 |
| CYLD | CYLD Lysine 63 Deubiquitinase | Q9NQC7 |
| ERCC2 | ERCC Excision Repair 2, TFIIH Core Complex Helicase Subunit | P18074 |
| AKT3 | AKT Serine/Threonine Kinase 3 | Q9Y243 |
| MAN2B1 | Mannosidase Alpha Class 2B Member 1 | O00754 |
| LINC02605 | Long Intergenic Non-Protein Coding RNA 2605 |  |
| ALPL | Alkaline Phosphatase, Biomineralization Associated | P05186 |
| H2AC18 | H2A Clustered Histone 18 | Q6FI13 |
| TLR3 | Toll Like Receptor 3 | O15455 |
| MIR23B | MicroRNA 23b |  |
| ANGPT2 | Angiopoietin 2 | O15123 |
| PTX3 | Pentraxin 3 | P26022 |
| C1QTNF5 | C1q And TNF Related 5 | Q9BXJ0 |
| SDHAF2 | Succinate Dehydrogenase Complex Assembly Factor 2 | Q9NX18 |
| HSPD1 | Heat Shock Protein Family D (Hsp60) Member 1 | P10809 |
| DMP1 | Dentin Matrix Acidic Phosphoprotein 1 | Q13316 |
| SMC1A | Structural Maintenance Of Chromosomes 1A | Q14683 |
| HOTTIP | HOXA Distal Transcript Antisense RNA |  |
| RAD21 | RAD21 Cohesin Complex Component | O60216 |
| AIFM1 | Apoptosis Inducing Factor Mitochondria Associated 1 | O95831 |
| PTGS1 | Prostaglandin-Endoperoxide Synthase 1 | P23219 |
| PLAU | Plasminogen Activator, Urokinase | P00749 |
| TANK | TRAF Family Member Associated NFKB Activator | Q92844 |
| PTPRC | Protein Tyrosine Phosphatase Receptor Type C | P08575 |
| CDK5RAP2 | CDK5 Regulatory Subunit Associated Protein 2 | Q96SN8 |
| IL11 | Interleukin 11 | P20809 |
| IRF3 | Interferon Regulatory Factor 3 | Q14653 |
| HSP90AA1 | Heat Shock Protein 90 Alpha Family Class A Member 1 | P07900 |
| MIR140 | MicroRNA 140 |  |
| AGTR1 | Angiotensin II Receptor Type 1 | P30556 |
| TSC1 | TSC Complex Subunit 1 | Q92574 |
| HLA-A | Major Histocompatibility Complex, Class I, A | P04439 |
| TG | Thyroglobulin | P01266 |
| STXBP1 | Syntaxin Binding Protein 1 | P61764 |
| MUC1 | Mucin 1, Cell Surface Associated | P15941 |
| MIR181D | MicroRNA 181d |  |
| MPO | Myeloperoxidase | P05164 |
| GUSB | Glucuronidase Beta | P08236 |
| TRIP11 | Thyroid Hormone Receptor Interactor 11 | Q15643 |
| SCN1A | Sodium Voltage-Gated Channel Alpha Subunit 1 | P35498 |
| IRS1 | Insulin Receptor Substrate 1 | P35568 |
| RPS27 | Ribosomal Protein S27 | P42677 |
| CCN2 | Cellular Communication Network Factor 2 | P29279 |
| CYP3A4 | Cytochrome P450 Family 3 Subfamily A Member 4 | P08684 |
| INPP5K | Inositol Polyphosphate-5-Phosphatase K | Q9BT40 |
| ARSB | Arylsulfatase B | P15848 |
| DES | Desmin | P17661 |
| WDR62 | WD Repeat Domain 62 | O43379 |
| MT-CO1 | Mitochondrially Encoded Cytochrome C Oxidase I | P00395 |
| PON1 | Paraoxonase 1 | P27169 |
| PLA2G6 | Phospholipase A2 Group VI | O60733 |
| HSPA1A | Heat Shock Protein Family A (Hsp70) Member 1A | P0DMV8 |
| CCN6 | Cellular Communication Network Factor 6 | O95389 |
| SMARCA4 | SWI/SNF Related, Matrix Associated, Actin Dependent Regulator Of Chromatin, Subfamily A, Member 4 | P51532 |
| STING1 | Stimulator Of Interferon Response CGAMP Interactor 1 | Q86WV6 |
| MIR181C | MicroRNA 181c |  |
| MAP3K14 | Mitogen-Activated Protein Kinase Kinase Kinase 14 | Q99558 |
| SIL1 | SIL1 Nucleotide Exchange Factor | Q9H173 |
| MIF | Macrophage Migration Inhibitory Factor | P14174 |
| NEAT1 | Nuclear Paraspeckle Assembly Transcript 1 |  |
| WNT10B | Wnt Family Member 10B | O00744 |
| MADD | MAP Kinase Activating Death Domain | Q8WXG6 |
| DNAJC21 | DnaJ Heat Shock Protein Family (Hsp40) Member C21 | Q5F1R6 |
| CAV1 | Caveolin 1 | Q03135 |
| HULC | Hepatocellular Carcinoma Up-Regulated Long Non-Coding RNA |  |
| PEPD | Peptidase D | P12955 |
| CCL11 | C-C Motif Chemokine Ligand 11 | P51671 |
| AR | Androgen Receptor | P10275 |
| MYD88 | MYD88 Innate Immune Signal Transduction Adaptor | Q99836 |
| ITGB1 | Integrin Subunit Beta 1 | P05556 |
| COL5A2 | Collagen Type V Alpha 2 Chain | P05997 |
| EIF2AK3 | Eukaryotic Translation Initiation Factor 2 Alpha Kinase 3 | Q9NZJ5 |
| CCAT1 | Colon Cancer Associated Transcript 1 |  |
| IFNA2 | Interferon Alpha 2 | P01563 |
| TLR9 | Toll Like Receptor 9 | Q9NR96 |
| SRCAP | Snf2 Related CREBBP Activator Protein | Q6ZRS2 |
| CCR5 | C-C Motif Chemokine Receptor 5 | P51681 |
| NR3C1 | Nuclear Receptor Subfamily 3 Group C Member 1 | P04150 |
| NHP2 | NHP2 Ribonucleoprotein | Q9NX24 |
| KMT2D | Lysine Methyltransferase 2D | O14686 |
| KRT8 | Keratin 8 | P05787 |
| TFAP2A | Transcription Factor AP-2 Alpha | P05549 |
| CEP135 | Centrosomal Protein 135 | Q66GS9 |
| KNG1 | Kininogen 1 | P01042 |
| EXTL3 | Exostosin Like Glycosyltransferase 3 | O43909 |
| LCN2 | Lipocalin 2 | P80188 |
| FGFR4 | Fibroblast Growth Factor Receptor 4 | P22455 |
| RPS6KA3 | Ribosomal Protein S6 Kinase A3 | P51812 |
| MIR130A | MicroRNA 130a |  |
| PRL | Prolactin | P01236 |
| ARID1B | AT-Rich Interaction Domain 1B | Q8NFD5 |
| MIR92A1 | MicroRNA 92a-1 |  |
| MIR125B1 | MicroRNA 125b-1 |  |
| GSK3B | Glycogen Synthase Kinase 3 Beta | P49841 |
| S100B | S100 Calcium Binding Protein B | P04271 |
| CYP1A2 | Cytochrome P450 Family 1 Subfamily A Member 2 | P05177 |
| C4A | Complement C4A (Rodgers Blood Group) | P0C0L4 |
| KRT14 | Keratin 14 | P02533 |
| MIR149 | MicroRNA 149 |  |
| ACTG1 | Actin Gamma 1 | P63261 |
| PDCD6IP | Programmed Cell Death 6 Interacting Protein | Q8WUM4 |
| SMPD1 | Sphingomyelin Phosphodiesterase 1 | P17405 |
| NOP10 | NOP10 Ribonucleoprotein | Q9NPE3 |
| APP | Amyloid Beta Precursor Protein | P05067 |
| ZMPSTE24 | Zinc Metallopeptidase STE24 | O75844 |
| EMSLR | E2F1 MRNA Stabilizing LncRNA |  |
| MMP12 | Matrix Metallopeptidase 12 | P39900 |
| PPARA | Peroxisome Proliferator Activated Receptor Alpha | Q07869 |
| TUBB3 | Tubulin Beta 3 Class III | Q13509 |
| SP7 | Sp7 Transcription Factor | Q8TDD2 |
| MYH7 | Myosin Heavy Chain 7 | P12883 |
| IL5 | Interleukin 5 | P05113 |
| ACE2 | Angiotensin Converting Enzyme 2 | Q9BYF1 |
| TBX4 | T-Box Transcription Factor 4 | P57082 |
| CASP2 | Caspase 2 | P42575 |
| CYP2D6 | Cytochrome P450 Family 2 Subfamily D Member 6 | P10635 |
| CHST3 | Carbohydrate Sulfotransferase 3 | Q7LGC8 |
| SERPINA1 | Serpin Family A Member 1 | P01009 |
| NFATC1 | Nuclear Factor Of Activated T Cells 1 | O95644 |
| TCOF1 | Treacle Ribosome Biogenesis Factor 1 | Q13428 |
| SERPINF1 | Serpin Family F Member 1 | P36955 |
| CCNA2 | Cyclin A2 | P20248 |
| PGR-AS1 | PGR Antisense RNA 1 |  |
| ASXL1 | ASXL Transcriptional Regulator 1 | Q8IXJ9 |
| CCL3 | C-C Motif Chemokine Ligand 3 | P10147 |
| NOTCH2 | Notch Receptor 2 | Q04721 |
| MIR27A | MicroRNA 27a |  |
| LDLR | Low Density Lipoprotein Receptor | P01130 |
| ABL1 | ABL Proto-Oncogene 1, Non-Receptor Tyrosine Kinase | P00519 |
| IL12A | Interleukin 12A | P29459 |
| SPTAN1 | Spectrin Alpha, Non-Erythrocytic 1 | Q13813 |
| UMOD | Uromodulin | P07911 |
| CD34 | CD34 Molecule | P28906 |
| EXT2 | Exostosin Glycosyltransferase 2 | Q93063 |
| MAPK10 | Mitogen-Activated Protein Kinase 10 | P53779 |
| CASC2 | Cancer Susceptibility 2 | Q8IU53 |
| NOTCH3 | Notch Receptor 3 | Q9UM47 |
| LTBP2 | Latent Transforming Growth Factor Beta Binding Protein 2 | Q14767 |
| TRAPPC10 | Trafficking Protein Particle Complex Subunit 10 | P48553 |
| ITGB3 | Integrin Subunit Beta 3 | P05106 |
| FANCB | FA Complementation Group B | Q8NB91 |
| SAMHD1 | SAM And HD Domain Containing Deoxynucleoside Triphosphate Triphosphohydrolase 1 | Q9Y3Z3 |
| WRN | WRN RecQ Like Helicase | Q14191 |
| NOG | Noggin | Q13253 |
| OFD1 | OFD1 Centriole And Centriolar Satellite Protein | O75665 |
| SATB2 | SATB Homeobox 2 | Q9UPW6 |
| GATA1 | GATA Binding Protein 1 | P15976 |
| PRKN | Parkin RBR E3 Ubiquitin Protein Ligase | O60260 |
| CP | Ceruloplasmin | P00450 |
| ACVR1B | Activin A Receptor Type 1B | P36896 |
| IRF1 | Interferon Regulatory Factor 1 | P10914 |
| BMP1 | Bone Morphogenetic Protein 1 | P13497 |
| GLI2 | GLI Family Zinc Finger 2 | P10070 |
| THBS1 | Thrombospondin 1 | P07996 |
| PSTPIP1 | Proline-Serine-Threonine Phosphatase Interacting Protein 1 | O43586 |
| CDKN2B | Cyclin Dependent Kinase Inhibitor 2B | P42772 |
| MIRLET7C | MicroRNA Let-7c |  |
| ORC1 | Origin Recognition Complex Subunit 1 | Q13415 |
| CYP19A1 | Cytochrome P450 Family 19 Subfamily A Member 1 | P11511 |
| MCM7 | Minichromosome Maintenance Complex Component 7 | P33993 |
| SELL | Selectin L | P14151 |
| MAPT | Microtubule Associated Protein Tau | P10636 |
| IRAK1 | Interleukin 1 Receptor Associated Kinase 1 | P51617 |
| HNF1A-AS1 | HNF1A Antisense RNA 1 |  |
| SERPINA3 | Serpin Family A Member 3 | P01011 |
| RASSF1 | Ras Association Domain Family Member 1 | Q9NS23 |
| DLK1 | Delta Like Non-Canonical Notch Ligand 1 | P80370 |
| CXCR3 | C-X-C Motif Chemokine Receptor 3 | P49682 |
| FLNC | Filamin C | Q14315 |
| FOSL1 | FOS Like 1, AP-1 Transcription Factor Subunit | P15407 |
| CEBPB | CCAAT Enhancer Binding Protein Beta | P17676 |
| RPL13 | Ribosomal Protein L13 | P26373 |
| CPT2 | Carnitine Palmitoyltransferase 2 | P23786 |
| AGER | Advanced Glycosylation End-Product Specific Receptor | Q15109 |
| IL6ST | Interleukin 6 Cytokine Family Signal Transducer | P40189 |
| FLT1 | Fms Related Receptor Tyrosine Kinase 1 | P17948 |
| PECAM1 | Platelet And Endothelial Cell Adhesion Molecule 1 | P16284 |
| RIGI | RNA Sensor RIG-I | O95786 |
| ANGPT1 | Angiopoietin 1 | Q15389 |
| SYNE1 | Spectrin Repeat Containing Nuclear Envelope Protein 1 | Q8NF91 |
| SOX6 | SRY-Box Transcription Factor 6 | P35712 |
| ZEB2 | Zinc Finger E-Box Binding Homeobox 2 | O60315 |
| MIR133B | MicroRNA 133b |  |
| TF | Transferrin | P02787 |
| CRADD | CASP2 And RIPK1 Domain Containing Adaptor With Death Domain | P78560 |
| ADH1B | Alcohol Dehydrogenase 1B (Class I), Beta Polypeptide | P00325 |
| LPL | Lipoprotein Lipase | P06858 |
| ASAH1 | N-Acylsphingosine Amidohydrolase 1 | Q13510 |
| IGF2R | Insulin Like Growth Factor 2 Receptor | P11717 |
| WWOX | WW Domain Containing Oxidoreductase | Q9NZC7 |
| APOL1 | Apolipoprotein L1 | O14791 |
| CLCN1 | Chloride Voltage-Gated Channel 1 | P35523 |
| MIR15B | MicroRNA 15b |  |
| NTRK2 | Neurotrophic Receptor Tyrosine Kinase 2 | Q16620 |
| LAMA5 | Laminin Subunit Alpha 5 | O15230 |
| NOS1 | Nitric Oxide Synthase 1 | P29475 |
| INSR | Insulin Receptor | P06213 |
| SLC17A5 | Solute Carrier Family 17 Member 5 | Q9NRA2 |
| ORC6 | Origin Recognition Complex Subunit 6 | Q9Y5N6 |
| SNCA | Synuclein Alpha | P37840 |
| SST | Somatostatin | P61278 |
| CSGALNACT1 | Chondroitin Sulfate N-Acetylgalactosaminyltransferase 1 | Q8TDX6 |
| WNT3 | Wnt Family Member 3 | P56703 |
| APEX1 | Apurinic/Apyrimidinic Endodeoxyribonuclease 1 | P27695 |
| IL33 | Interleukin 33 | O95760 |
| SMARCA2 | SWI/SNF Related, Matrix Associated, Actin Dependent Regulator Of Chromatin, Subfamily A, Member 2 | P51531 |
| ERAP1 | Endoplasmic Reticulum Aminopeptidase 1 | Q9NZ08 |
| TGIF1 | TGFB Induced Factor Homeobox 1 | Q15583 |
| MIR181A2 | MicroRNA 181a-2 |  |
| LIF | LIF Interleukin 6 Family Cytokine | P15018 |
| SELENON | Selenoprotein N | Q9NZV5 |
| CCL4 | C-C Motif Chemokine Ligand 4 | P13236 |
| KNL1 | Kinetochore Scaffold 1 | Q8NG31 |
| MIR486-1 | MicroRNA 486-1 |  |
| SRP54 | Signal Recognition Particle 54 | P61011 |
| BRD4 | Bromodomain Containing 4 | O60885 |
| PAX1 | Paired Box 1 | P15863 |
| ROR2 | Receptor Tyrosine Kinase Like Orphan Receptor 2 | Q01974 |
| MMP10 | Matrix Metallopeptidase 10 | P09238 |
| AREG | Amphiregulin | P15514 |
| IDH1 | Isocitrate Dehydrogenase (NADP(+)) 1 | O75874 |
| THSD4 | Thrombospondin Type 1 Domain Containing 4 | Q6ZMP0 |
| GH1 | Growth Hormone 1 | P01241 |
| TUBB2B | Tubulin Beta 2B Class IIb | Q9BVA1 |
| CSNK2A1 | Casein Kinase 2 Alpha 1 | P68400 |
| GLI1 | GLI Family Zinc Finger 1 | P08151 |
| MIR106B | MicroRNA 106b |  |
| VTN | Vitronectin | P04004 |
| SMC3 | Structural Maintenance Of Chromosomes 3 | Q9UQE7 |
| GGT1 | Gamma-Glutamyltransferase 1 | P19440 |
| MMP7 | Matrix Metallopeptidase 7 | P09237 |
| ATRIP-TREX1 | ATRIP-TREX1 Readthrough |  |
| CDKN1C | Cyclin Dependent Kinase Inhibitor 1C | P49918 |
| DCN | Decorin | P07585 |
| SIRT1 | Sirtuin 1 | Q96EB6 |
| TCF4 | Transcription Factor 4 | P15884 |
| LAMA2 | Laminin Subunit Alpha 2 | P24043 |
| PDGFB | Platelet Derived Growth Factor Subunit B | P01127 |
| NRP1 | Neuropilin 1 | O14786 |
| SKIC3 | SKI3 Subunit Of Superkiller Complex | Q6PGP7 |
| CEP63 | Centrosomal Protein 63 | Q96MT8 |
| HSPA4 | Heat Shock Protein Family A (Hsp70) Member 4 | P34932 |
| ACVR1 | Activin A Receptor Type 1 | Q04771 |
| TSHR | Thyroid Stimulating Hormone Receptor | P16473 |
| MIR493HG | MIR493 Cluster Host Gene |  |
| ENSG00000276919 | |  |
| PLOD2 | Procollagen-Lysine,2-Oxoglutarate 5-Dioxygenase 2 | O00469 |
| EFL1 | Elongation Factor Like GTPase 1 | Q7Z2Z2 |
| CEBPA | CCAAT Enhancer Binding Protein Alpha | P49715 |
| LTBP1 | Latent Transforming Growth Factor Beta Binding Protein 1 | Q14766 |
| MCOLN1 | Mucolipin TRP Cation Channel 1 | Q9GZU1 |
| MIR203A | MicroRNA 203a |  |
| MIR133A1 | MicroRNA 133a-1 |  |
| PLCG1 | Phospholipase C Gamma 1 | P19174 |
| RUNX1 | RUNX Family Transcription Factor 1 | Q01196 |
| UCA1 | Urothelial Cancer Associated 1 |  |
| HSPA5 | Heat Shock Protein Family A (Hsp70) Member 5 | P11021 |
| ADAMTS4 | ADAM Metallopeptidase With Thrombospondin Type 1 Motif 4 | O75173 |
| CASK | Calcium/Calmodulin Dependent Serine Protein Kinase | O14936 |
| PGR | Progesterone Receptor | P06401 |
| NEFL | Neurofilament Light Chain | P07196 |
| PLAUR | Plasminogen Activator, Urokinase Receptor | Q03405 |
| PRKCZ | Protein Kinase C Zeta | Q05513 |
| SPARC | Secreted Protein Acidic And Cysteine Rich | P09486 |
| CHD4 | Chromodomain Helicase DNA Binding Protein 4 | Q14839 |
| AKT2 | AKT Serine/Threonine Kinase 2 | P31751 |
| ADAM10 | ADAM Metallopeptidase Domain 10 | O14672 |
| SASS6 | SAS-6 Centriolar Assembly Protein | Q6UVJ0 |
| MIR3200 | MicroRNA 3200 |  |
| MPZ | Myelin Protein Zero | P25189 |
| NPY | Neuropeptide Y | P01303 |
| KCNQ1OT1 | KCNQ1 Opposite Strand/Antisense Transcript 1 |  |
| DLX5 | Distal-Less Homeobox 5 | P56178 |
| ALX4 | ALX Homeobox 4 | Q9H161 |
| SHBG | Sex Hormone Binding Globulin | P04278 |
| CYP27B1 | Cytochrome P450 Family 27 Subfamily B Member 1 | O15528 |
| GALC | Galactosylceramidase | P54803 |
| GMNN | Geminin DNA Replication Inhibitor | O75496 |
| SREBF1 | Sterol Regulatory Element Binding Transcription Factor 1 | P36956 |
| DSPP | Dentin Sialophosphoprotein | Q9NZW4 |
| FGF1 | Fibroblast Growth Factor 1 | P05230 |
| F10 | Coagulation Factor X | P00742 |
| TTR | Transthyretin | P02766 |
| WNT10A | Wnt Family Member 10A | Q9GZT5 |
| FHIT | Fragile Histidine Triad Diadenosine Triphosphatase | P49789 |
| ALPP | Alkaline Phosphatase, Placental | P05187 |
| BCL11B | BCL11 Transcription Factor B | Q9C0K0 |
| NPPB | Natriuretic Peptide B | P16860 |
| MIR126 | MicroRNA 126 |  |
| CCNB1 | Cyclin B1 | P14635 |
| DICER1 | Dicer 1, Ribonuclease III | Q9UPY3 |
| CHKA | Choline Kinase Alpha | P35790 |
| SCARB2 | Scavenger Receptor Class B Member 2 | Q14108 |
| IFT57 | Intraflagellar Transport 57 | Q9NWB7 |
| MIAT | Myocardial Infarction Associated Transcript |  |
| EXOSC3 | Exosome Component 3 | Q9NQT5 |
| MAP3K1 | Mitogen-Activated Protein Kinase Kinase Kinase 1 | Q13233 |
| SAA1 | Serum Amyloid A1 | P0DJI8 |
| CTSB | Cathepsin B | P07858 |
| CBL | Cbl Proto-Oncogene | P22681 |
| PAX3 | Paired Box 3 | P23760 |
| EHMT1 | Euchromatic Histone Lysine Methyltransferase 1 | Q9H9B1 |
| RNASEH2C | Ribonuclease H2 Subunit C | Q8TDP1 |
| CSPP1 | Centrosome And Spindle Pole Associated Protein 1 | Q1MSJ5 |
| ADAMTSL1 | ADAMTS Like 1 | Q8N6G6 |
| MIR212 | MicroRNA 212 |  |
| MEGF8 | Multiple EGF Like Domains 8 | Q7Z7M0 |
| ZNF335 | Zinc Finger Protein 335 | Q9H4Z2 |
| HFE | Homeostatic Iron Regulator | Q30201 |
| PPT1 | Palmitoyl-Protein Thioesterase 1 | P50897 |
| SQSTM1 | Sequestosome 1 | Q13501 |
| TBX1 | T-Box Transcription Factor 1 | O43435 |
| DDRGK1 | DDRGK Domain Containing 1 | Q96HY6 |
| UFC1 | Ubiquitin-Fold Modifier Conjugating Enzyme 1 | Q9Y3C8 |
| EZR | Ezrin | P15311 |
| MBP | Myelin Basic Protein | P02686 |
| COG4 | Component Of Oligomeric Golgi Complex 4 | Q9H9E3 |
| SFRP4 | Secreted Frizzled Related Protein 4 | Q6FHJ7 |
| GRN | Granulin Precursor | P28799 |
| ATP6V0A2 | ATPase H+ Transporting V0 Subunit A2 | Q9Y487 |
| MBL2 | Mannose Binding Lectin 2 | P11226 |
| COX5A | Cytochrome C Oxidase Subunit 5A | P20674 |
| FLT4 | Fms Related Receptor Tyrosine Kinase 4 | P35916 |
| AHSG | Alpha 2-HS Glycoprotein | P02765 |
| PLG | Plasminogen | P00747 |
| IL18R1 | Interleukin 18 Receptor 1 | Q13478 |
| PRTN3 | Proteinase 3 | P24158 |
| KIF14 | Kinesin Family Member 14 | Q15058 |
| BMPR1A | Bone Morphogenetic Protein Receptor Type 1A | P36894 |
| FOXO1 | Forkhead Box O1 | Q12778 |
| ALOX5 | Arachidonate 5-Lipoxygenase | P09917 |
| PRG4 | Proteoglycan 4 | Q92954 |
| MIR124-1 | MicroRNA 124-1 |  |
| CCR1 | C-C Motif Chemokine Receptor 1 | P32246 |
| PAFAH1B1 | Platelet Activating Factor Acetylhydrolase 1b Regulatory Subunit 1 | P43034 |
| DDX3X | DEAD-Box Helicase 3 X-Linked | O00571 |
| UNC45A | Unc-45 Myosin Chaperone A | Q9H3U1 |
| OBSL1 | Obscurin Like Cytoskeletal Adaptor 1 | O75147 |
| HDAC4 | Histone Deacetylase 4 | P56524 |
| LHX4 | LIM Homeobox 4 | Q969G2 |
| ITGB2 | Integrin Subunit Beta 2 | P05107 |
| IL7 | Interleukin 7 | P13232 |
| RECQL4 | RecQ Like Helicase 4 | O94761 |
| IFI27 | Interferon Alpha Inducible Protein 27 | P40305 |
| SLC10A7 | Solute Carrier Family 10 Member 7 | Q0GE19 |
| PRKDC | Protein Kinase, DNA-Activated, Catalytic Subunit | P78527 |
| TBC1D24 | TBC1 Domain Family Member 24 | Q9ULP9 |
| JAK1 | Janus Kinase 1 | P23458 |
| MYOD1 | Myogenic Differentiation 1 | P15172 |
| LRP6 | LDL Receptor Related Protein 6 | O75581 |
| TBK1 | TANK Binding Kinase 1 | Q9UHD2 |
| PRPF8 | Pre-MRNA Processing Factor 8 | Q6P2Q9 |
| MIRLET7A1 | MicroRNA Let-7a-1 |  |
| SMN1 | Survival Of Motor Neuron 1, Telomeric | Q16637 |
| SERPINH1 | Serpin Family H Member 1 | P50454 |
| TRAPPC11 | Trafficking Protein Particle Complex Subunit 11 | Q7Z392 |
| LRP2 | LDL Receptor Related Protein 2 | P98164 |
| HBEGF | Heparin Binding EGF Like Growth Factor | Q99075 |
| TRAF3IP1 | TRAF3 Interacting Protein 1 | Q8TDR0 |
| MMP8 | Matrix Metallopeptidase 8 | P22894 |
| TAC1 | Tachykinin Precursor 1 | P20366 |
| FKRP | Fukutin Related Protein | Q9H9S5 |
| SGCA | Sarcoglycan Alpha | Q16586 |
| CRH | Corticotropin Releasing Hormone | P06850 |
| ADNP | Activity Dependent Neuroprotector Homeobox | Q9H2P0 |
| TPO | Thyroid Peroxidase | P07202 |
| TTN-AS1 | TTN Antisense RNA 1 |  |
| SCARNA5 | Small Cajal Body-Specific RNA 5 |  |
| COPB2 | COPI Coat Complex Subunit Beta 2 | P35606 |
| HDAC8 | Histone Deacetylase 8 | Q9BY41 |
| VCL | Vinculin | P18206 |
| ETS1 | ETS Proto-Oncogene 1, Transcription Factor | P14921 |
| DHFR | Dihydrofolate Reductase | P00374 |
| RPS19 | Ribosomal Protein S19 | P39019 |
| TFRC | Transferrin Receptor | P02786 |
| PFAS | Phosphoribosylformylglycinamidine Synthase | O15067 |
| NSD2 | Nuclear Receptor Binding SET Domain Protein 2 | O96028 |
| MSX1 | Msh Homeobox 1 | P28360 |
| GJB2 | Gap Junction Protein Beta 2 | P29033 |
| MIR93 | MicroRNA 93 |  |
| GREM1 | Gremlin 1, DAN Family BMP Antagonist | O60565 |
| FUCA1 | Alpha-L-Fucosidase 1 | P04066 |
| TUG1 | Taurine Up-Regulated 1 | A0A6I8PU40 |
| MYOG | Myogenin | P15173 |
| SOX4 | SRY-Box Transcription Factor 4 | Q06945 |
| THPO | Thrombopoietin | P40225 |
| OLR1 | Oxidized Low Density Lipoprotein Receptor 1 | P78380 |
| HMGCR | 3-Hydroxy-3-Methylglutaryl-CoA Reductase | P04035 |
| CSF1R | Colony Stimulating Factor 1 Receptor | P07333 |
| PRKCA | Protein Kinase C Alpha | P17252 |
| RHO | Rhodopsin | P08100 |
| RPL5 | Ribosomal Protein L5 | P46777 |
| IGFBP7 | Insulin Like Growth Factor Binding Protein 7 | Q16270 |
| LOC113939944 | Sharpr-MPRA Regulatory Region 9539 |  |
| CD163 | CD163 Molecule | Q86VB7 |
| COMT | Catechol-O-Methyltransferase | P21964 |
| SOX10 | SRY-Box Transcription Factor 10 | P56693 |
| MIR132 | MicroRNA 132 |  |
| CYP2C19 | Cytochrome P450 Family 2 Subfamily C Member 19 | P33261 |
| HOXA11-AS | HOXA11 Antisense RNA |  |
| SPHK1 | Sphingosine Kinase 1 | Q9NYA1 |
| COL4A2 | Collagen Type IV Alpha 2 Chain | P08572 |
| PTK2B | Protein Tyrosine Kinase 2 Beta | Q14289 |
| ESR2 | Estrogen Receptor 2 | Q92731 |
| SLC12A2 | Solute Carrier Family 12 Member 2 | P55011 |
| CHGA | Chromogranin A | P10645 |
| TRIP4 | Thyroid Hormone Receptor Interactor 4 | Q15650 |
| SNORD15A | Small Nucleolar RNA, C/D Box 15A |  |
| RPL26 | Ribosomal Protein L26 | P61254 |
| SMO | Smoothened, Frizzled Class Receptor | Q99835 |
| LEPR | Leptin Receptor | P48357 |
| SGSH | N-Sulfoglucosamine Sulfohydrolase | P51688 |
| DNMT3A | DNA Methyltransferase 3 Alpha | Q9Y6K1 |
| CD68 | CD68 Molecule | P34810 |
| PITX2 | Paired Like Homeodomain 2 | Q99697 |
| PORCN | Porcupine O-Acyltransferase | Q9H237 |
| TIMP4 | TIMP Metallopeptidase Inhibitor 4 | Q99727 |
| EPOR | Erythropoietin Receptor | P19235 |
| RNASEH2B | Ribonuclease H2 Subunit B | Q5TBB1 |
| PSEN1 | Presenilin 1 | P49768 |
| GDNF | Glial Cell Derived Neurotrophic Factor | P39905 |
| MSH6 | MutS Homolog 6 | P52701 |
| KL | Klotho | Q9UEF7 |
| CDK1 | Cyclin Dependent Kinase 1 | P06493 |
| GSDME | Gasdermin E | O60443 |
| PSAP | Prosaposin | P07602 |
| TPP1 | Tripeptidyl Peptidase 1 | O14773 |
| RPS20 | Ribosomal Protein S20 | P60866 |
| FGF23 | Fibroblast Growth Factor 23 | Q9GZV9 |
| OPRM1 | Opioid Receptor Mu 1 | P35372 |
| EMG1 | EMG1 N1-Specific Pseudouridine Methyltransferase | Q92979 |
| MBTPS1 | Membrane Bound Transcription Factor Peptidase, Site 1 | Q14703 |
| DONSON | DNA Replication Fork Stabilization Factor DONSON | Q9NYP3 |
| PPARGC1A | PPARG Coactivator 1 Alpha | Q9UBK2 |
| WNT3A | Wnt Family Member 3A | P56704 |
| DNAH8 | Dynein Axonemal Heavy Chain 8 | Q96JB1 |
| BSG | Basigin (Ok Blood Group) | P35613 |
| XRCC5 | X-Ray Repair Cross Complementing 5 | P13010 |
| MIR92A2 | MicroRNA 92a-2 |  |
| POU1F1 | POU Class 1 Homeobox 1 | P28069 |
| PWAR1 | Prader Willi/Angelman Region RNA 1 |  |
| ZEB1 | Zinc Finger E-Box Binding Homeobox 1 | P37275 |
| SLC2A4 | Solute Carrier Family 2 Member 4 | P14672 |
| NKX3-2 | NK3 Homeobox 2 | P78367 |
| PDHA1 | Pyruvate Dehydrogenase E1 Subunit Alpha 1 | P08559 |
| DAG1 | Dystroglycan 1 | Q14118 |
| MIR499A | MicroRNA 499a |  |
| CC2D2A | Coiled-Coil And C2 Domain Containing 2A | Q9P2K1 |
| FOXJ1 | Forkhead Box J1 | Q92949 |
| MGP | Matrix Gla Protein | P08493 |
| PROP1 | PROP Paired-Like Homeobox 1 | O75360 |
| MEN1 | Menin 1 | O00255 |
| CDC45 | Cell Division Cycle 45 | O75419 |
| RPS6KB1 | Ribosomal Protein S6 Kinase B1 | P23443 |
| IARS2 | Isoleucyl-TRNA Synthetase 2, Mitochondrial | Q9NSE4 |
| HDAC9 | Histone Deacetylase 9 | Q9UKV0 |
| MMACHC | Metabolism Of Cobalamin Associated C | Q9Y4U1 |
| SNAP25 | Synaptosome Associated Protein 25 | P60880 |
| VCP | Valosin Containing Protein | P55072 |
| HSPA9 | Heat Shock Protein Family A (Hsp70) Member 9 | P38646 |
| ALK | ALK Receptor Tyrosine Kinase | Q9UM73 |
| ITGAM | Integrin Subunit Alpha M | P11215 |
| GATA2 | GATA Binding Protein 2 | P23769 |
| MEPE | Matrix Extracellular Phosphoglycoprotein | Q9NQ76 |
| MIR28 | MicroRNA 28 |  |
| DNMT1 | DNA Methyltransferase 1 | P26358 |
| DLX2 | Distal-Less Homeobox 2 | Q07687 |
| PROS1 | Protein S | P07225 |
| DYSF | Dysferlin | O75923 |
| MIRLET7B | MicroRNA Let-7b |  |
| OGA | O-GlcNAcase | O60502 |
| NANS | N-Acetylneuraminate Synthase | Q9NR45 |
| ALX3 | ALX Homeobox 3 | O95076 |
| GNRH1 | Gonadotropin Releasing Hormone 1 | P01148 |
| ATF4 | Activating Transcription Factor 4 | P18848 |
| CYBB | Cytochrome B-245 Beta Chain | P04839 |
| PDPN | Podoplanin | Q86YL7 |
| PI4KA | Phosphatidylinositol 4-Kinase Alpha | P42356 |
| ANKH | ANKH Inorganic Pyrophosphate Transport Regulator | Q9HCJ1 |
| CARD14 | Caspase Recruitment Domain Family Member 14 | Q9BXL6 |
| CLU | Clusterin | P10909 |
| RPL11 | Ribosomal Protein L11 | P62913 |
| ZFPM2 | Zinc Finger Protein, FOG Family Member 2 | Q8WW38 |
| EED | Embryonic Ectoderm Development | O75530 |
| KDM4C | Lysine Demethylase 4C | Q9H3R0 |
| ATF2 | Activating Transcription Factor 2 | P15336 |
| SOCS3 | Suppressor Of Cytokine Signaling 3 | O14543 |
| AGT | Angiotensinogen | P01019 |
| FGA | Fibrinogen Alpha Chain | P02671 |
| XRCC6 | X-Ray Repair Cross Complementing 6 | P12956 |
| COL6A3 | Collagen Type VI Alpha 3 Chain | P12111 |
| USH2A | Usherin | O75445 |
| SREBF2 | Sterol Regulatory Element Binding Transcription Factor 2 | Q12772 |
| FGF3 | Fibroblast Growth Factor 3 | P11487 |
| DAPK1 | Death Associated Protein Kinase 1 | P53355 |
| GNS | Glucosamine (N-Acetyl)-6-Sulfatase | P15586 |
| STAMBP | STAM Binding Protein | O95630 |
| DMD | Dystrophin | P11532 |
| HSPB2 | Heat Shock Protein Family B (Small) Member 2 | Q16082 |
| S100A9 | S100 Calcium Binding Protein A9 | P06702 |
| TRA-TGC7-1 | TRNA-Ala (Anticodon TGC) 7-1 |  |
| TRA-TGC5-1 | TRNA-Ala (Anticodon TGC) 5-1 |  |
| ACADVL | Acyl-CoA Dehydrogenase Very Long Chain | P49748 |
| KDM6A | Lysine Demethylase 6A | O15550 |
| AFF3 | ALF Transcription Elongation Factor 3 | P51826 |
| MIR185 | MicroRNA 185 |  |
| ALS2 | Alsin Rho Guanine Nucleotide Exchange Factor ALS2 | Q96Q42 |
| FGG | Fibrinogen Gamma Chain | P02679 |
| TEK | TEK Receptor Tyrosine Kinase | Q02763 |
| SMAD7 | SMAD Family Member 7 | O15105 |
| F2RL1 | F2R Like Trypsin Receptor 1 | P55085 |
| CDK5 | Cyclin Dependent Kinase 5 | Q00535 |
| WDR35 | WD Repeat Domain 35 | Q9P2L0 |
| ATP1A2 | ATPase Na+/K+ Transporting Subunit Alpha 2 | P50993 |
| PDGFRA | Platelet Derived Growth Factor Receptor Alpha | P16234 |
| RASA1 | RAS P21 Protein Activator 1 | P20936 |
| PTDSS1 | Phosphatidylserine Synthase 1 | P48651 |
| NGLY1 | N-Glycanase 1 | Q96IV0 |
| DDIT3 | DNA Damage Inducible Transcript 3 | P35638 |
| XK | X-Linked Kx Blood Group Antigen, Kell And VPS13A Binding Protein | P51811 |
| CCL20 | C-C Motif Chemokine Ligand 20 | P78556 |
| LNCRNA-ATB | LncRNA Activated By TGF-Beta |  |
| CYBA | Cytochrome B-245 Alpha Chain | P13498 |
| XDH | Xanthine Dehydrogenase | P47989 |
| POLR1C | RNA Polymerase I And III Subunit C | O15160 |
| LTF | Lactotransferrin | P02788 |
| MITF | Melanocyte Inducing Transcription Factor | O75030 |
| GHR | Growth Hormone Receptor | P10912 |
| RRM2B | Ribonucleotide Reductase Regulatory TP53 Inducible Subunit M2B | Q7LG56 |
| FMR1 | Fragile X Messenger Ribonucleoprotein 1 | Q06787 |
| ORC4 | Origin Recognition Complex Subunit 4 | O43929 |
| FGF7 | Fibroblast Growth Factor 7 | P21781 |
| TRAPPC9 | Trafficking Protein Particle Complex Subunit 9 | Q96Q05 |
| HNRNPK | Heterogeneous Nuclear Ribonucleoprotein K | P61978 |
| COL4A5 | Collagen Type IV Alpha 5 Chain | P29400 |
| FGF9 | Fibroblast Growth Factor 9 | P31371 |
| ABCC9 | ATP Binding Cassette Subfamily C Member 9 | O60706 |
| TRC-GCA24-1 | TRNA-Cys (GCA) 24-1 |  |
| RNASEH2A | Ribonuclease H2 Subunit A | O75792 |
| CDT1 | Chromatin Licensing And DNA Replication Factor 1 | Q9H211 |
| COL4A3 | Collagen Type IV Alpha 3 Chain | Q01955 |
| ENTPD1 | Ectonucleoside Triphosphate Diphosphohydrolase 1 | P49961 |
| KIFBP | Kinesin Family Binding Protein | Q96EK5 |
| P2RX7 | Purinergic Receptor P2X 7 | Q99572 |
| FBLN5 | Fibulin 5 | Q9UBX5 |
| PAX7 | Paired Box 7 | P23759 |
| MYB | MYB Proto-Oncogene, Transcription Factor | P10242 |
| ABCA1 | ATP Binding Cassette Subfamily A Member 1 | O95477 |
| CYP21A2 | Cytochrome P450 Family 21 Subfamily A Member 2 | P08686 |
| GPC3 | Glypican 3 | P51654 |
| GNPTG | N-Acetylglucosamine-1-Phosphate Transferase Subunit Gamma | Q9UJJ9 |
| LEPQTL1 | Leptin, Serum Levels Of |  |
| CHD7 | Chromodomain Helicase DNA Binding Protein 7 | Q9P2D1 |
| SEC23B | SEC23 Homolog B, COPII Coat Complex Component | Q15437 |
| CDKN3 | Cyclin Dependent Kinase Inhibitor 3 | Q16667 |
| MCAM | Melanoma Cell Adhesion Molecule | P43121 |
| EWSR1 | EWS RNA Binding Protein 1 | Q01844 |
| WDR19 | WD Repeat Domain 19 | Q8NEZ3 |
| RXRA | Retinoid X Receptor Alpha | P19793 |
| FOXO3 | Forkhead Box O3 | O43524 |
| TRPV1 | Transient Receptor Potential Cation Channel Subfamily V Member 1 | Q8NER1 |
| TSR2 | TSR2 Ribosome Maturation Factor | Q969E8 |
| PHEX | Phosphate Regulating Endopeptidase X-Linked | P78562 |
| CEP290 | Centrosomal Protein 290 | O15078 |
| GCG | Glucagon | P01275 |
| EDARADD | EDAR Associated Via Death Domain | Q8WWZ3 |
| NPC2 | NPC Intracellular Cholesterol Transporter 2 | P61916 |
| ADM | Adrenomedullin | P35318 |
| SF3B2 | Splicing Factor 3b Subunit 2 | Q13435 |
| CYP17A1 | Cytochrome P450 Family 17 Subfamily A Member 1 | P05093 |
| ACVRL1 | Activin A Receptor Like Type 1 | P37023 |
| CIITA | Class II Major Histocompatibility Complex Transactivator | P33076 |
| PQBP1 | Polyglutamine Binding Protein 1 | O60828 |
| MIR4693 | MicroRNA 4693 |  |
| EFEMP1 | EGF Containing Fibulin Extracellular Matrix Protein 1 | Q12805 |
| RPS27A | Ribosomal Protein S27a | P62979 |
| DNAH9 | Dynein Axonemal Heavy Chain 9 | Q9NYC9 |
| GNB3 | G Protein Subunit Beta 3 | P16520 |
| EPAS1 | Endothelial PAS Domain Protein 1 | Q99814 |
| P4HB | Prolyl 4-Hydroxylase Subunit Beta | P07237 |
| SOX11 | SRY-Box Transcription Factor 11 | P35716 |
| HGSNAT | Heparan-Alpha-Glucosaminide N-Acetyltransferase | Q68CP4 |
| BCL10 | BCL10 Immune Signaling Adaptor | O95999 |
| GJB6 | Gap Junction Protein Beta 6 | O95452 |
| MIR532 | MicroRNA 532 |  |
| IKZF1 | IKAROS Family Zinc Finger 1 | Q13422 |
| CR2 | Complement C3d Receptor 2 | P20023 |
| SLC6A4 | Solute Carrier Family 6 Member 4 | P31645 |
| ADAMTS7 | ADAM Metallopeptidase With Thrombospondin Type 1 Motif 7 | Q9UKP4 |
| IFNAR1 | Interferon Alpha And Beta Receptor Subunit 1 | P17181 |
| PAH | Phenylalanine Hydroxylase | P00439 |
| TPM2 | Tropomyosin 2 | P07951 |
| DPAGT1 | Dolichyl-Phosphate N-Acetylglucosaminephosphotransferase 1 | Q9H3H5 |
| MECOM | MDS1 And EVI1 Complex Locus | Q03112 |
| KIF7 | Kinesin Family Member 7 | Q2M1P5 |
| MAX | MYC Associated Factor X | P61244 |
| RPS26 | Ribosomal Protein S26 | P62854 |
| SDC1 | Syndecan 1 | P18827 |
| MIR224 | MicroRNA 224 |  |
| ERN1 | Endoplasmic Reticulum To Nucleus Signaling 1 | O75460 |
| PROC | Protein C, Inactivator Of Coagulation Factors Va And VIIIa | P04070 |
| PGF | Placental Growth Factor | P49763 |
| EFEMP2 | EGF Containing Fibulin Extracellular Matrix Protein 2 | O95967 |
| CEACAM5 | CEA Cell Adhesion Molecule 5 | P06731 |
| AUTS2 | Activator Of Transcription And Developmental Regulator AUTS2 | Q8WXX7 |
| ERBB4 | Erb-B2 Receptor Tyrosine Kinase 4 | Q15303 |
| SAMD9 | Sterile Alpha Motif Domain Containing 9 | Q5K651 |
| ABCC1 | ATP Binding Cassette Subfamily C Member 1 | P33527 |
| KAT6B | Lysine Acetyltransferase 6B | Q8WYB5 |
| BRF1 | BRF1 RNA Polymerase III Transcription Initiation Factor Subunit | Q92994 |
| MIA2 | MIA SH3 Domain ER Export Factor 2 | Q96PC5 |
| UBA5 | Ubiquitin Like Modifier Activating Enzyme 5 | Q9GZZ9 |
| HYAL1 | Hyaluronidase 1 | Q12794 |
| FURIN | Furin, Paired Basic Amino Acid Cleaving Enzyme | P09958 |
| IDS | Iduronate 2-Sulfatase | P22304 |
| NXN | Nucleoredoxin | Q6DKJ4 |
| LINC01725 | Long Intergenic Non-Protein Coding RNA 1725 |  |
| LOC101927560 | Uncharacterized LOC101927560 |  |
| CX3CL1 | C-X3-C Motif Chemokine Ligand 1 | P78423 |
| CDH2 | Cadherin 2 | P19022 |
| FKBP14 | FKBP Prolyl Isomerase 14 | Q9NWM8 |
| SOX5 | SRY-Box Transcription Factor 5 | P35711 |
| MIR708 | MicroRNA 708 |  |
| LINC02895 | Long Intergenic Non-Protein Coding RNA 2895 |  |
| EPRS1 | Glutamyl-Prolyl-TRNA Synthetase 1 | P07814 |
| IGFBP5 | Insulin Like Growth Factor Binding Protein 5 | P24593 |
| SORL1 | Sortilin Related Receptor 1 | Q92673 |
| ADD1 | Adducin 1 | P35611 |
| TMEM216 | Transmembrane Protein 216 | Q9P0N5 |
| COL6A1 | Collagen Type VI Alpha 1 Chain | P12109 |
| SALL4 | Spalt Like Transcription Factor 4 | Q9UJQ4 |
| RPS24 | Ribosomal Protein S24 | P62847 |
| CSF3R | Colony Stimulating Factor 3 Receptor | Q99062 |
| GSR | Glutathione-Disulfide Reductase | P00390 |
| GDF15 | Growth Differentiation Factor 15 | Q99988 |
| DYNC2H1 | Dynein Cytoplasmic 2 Heavy Chain 1 | Q8NCM8 |
| RPS17 | Ribosomal Protein S17 | P08708 |
| MEG8 | Maternally Expressed 8, Small Nucleolar RNA Host Gene |  |
| SLPI | Secretory Leukocyte Peptidase Inhibitor | P03973 |
| WT1 | WT1 Transcription Factor | P19544 |
| NPC1 | NPC Intracellular Cholesterol Transporter 1 | O15118 |
| FASN | Fatty Acid Synthase | P49327 |
| IER3IP1 | Immediate Early Response 3 Interacting Protein 1 | Q9Y5U9 |
| CTNND2 | Catenin Delta 2 | Q9UQB3 |
| RPS10 | Ribosomal Protein S10 | P46783 |
| HTT | Huntingtin | P42858 |
| HSD11B1 | Hydroxysteroid 11-Beta Dehydrogenase 1 | P28845 |
| SEPSECS | Sep (O-Phosphoserine) TRNA:Sec (Selenocysteine) TRNA Synthase | Q9HD40 |
| SLC39A14 | Solute Carrier Family 39 Member 14 | Q15043 |
| PDE4D | Phosphodiesterase 4D | Q08499 |
| MIR135B | MicroRNA 135b |  |
| ABCC6 | ATP Binding Cassette Subfamily C Member 6 | O95255 |
| SCARF2 | Scavenger Receptor Class F Member 2 | Q96GP6 |
| GORAB | Golgin, RAB6 Interacting | Q5T7V8 |
| CRKL | CRK Like Proto-Oncogene, Adaptor Protein | P46109 |
| PRKAR1A | Protein Kinase CAMP-Dependent Type I Regulatory Subunit Alpha | P10644 |
| MEF2C | Myocyte Enhancer Factor 2C | Q06413 |
| HTR2A | 5-Hydroxytryptamine Receptor 2A | P28223 |
| AXIN1 | Axin 1 | O15169 |
| RPL15 | Ribosomal Protein L15 | P61313 |
| RPS29 | Ribosomal Protein S29 | P62273 |
| MAVS | Mitochondrial Antiviral Signaling Protein | Q7Z434 |
| ARCN1 | Archain 1 | P48444 |
| TWNK | Twinkle MtDNA Helicase | Q96RR1 |
| CHAT | Choline O-Acetyltransferase | P28329 |
| LRRK2 | Leucine Rich Repeat Kinase 2 | Q5S007 |
| TFPI | Tissue Factor Pathway Inhibitor | P10646 |
| PYY | Peptide YY | P10082 |
| ADAM9 | ADAM Metallopeptidase Domain 9 | Q13443 |
| IGFBP1 | Insulin Like Growth Factor Binding Protein 1 | P08833 |
| IL21 | Interleukin 21 | Q9HBE4 |
| FAT1 | FAT Atypical Cadherin 1 | Q14517 |
| LAMP2 | Lysosomal Associated Membrane Protein 2 | P13473 |
| CDH5 | Cadherin 5 | P33151 |
| TNC | Tenascin C | P24821 |
| CASR | Calcium Sensing Receptor | P41180 |
| DUOX2 | Dual Oxidase 2 | Q9NRD8 |
| PPIB | Peptidylprolyl Isomerase B | P23284 |
| SETBP1 | SET Binding Protein 1 | Q9Y6X0 |
| IL1R2 | Interleukin 1 Receptor Type 2 | P27930 |
| IGFBP2 | Insulin Like Growth Factor Binding Protein 2 | P18065 |
| SMAD1 | SMAD Family Member 1 | Q15797 |
| MEOX1 | Mesenchyme Homeobox 1 | P50221 |
| COL4A4 | Collagen Type IV Alpha 4 Chain | P53420 |
| NAMPT | Nicotinamide Phosphoribosyltransferase | P43490 |
| NOX4 | NADPH Oxidase 4 | Q9NPH5 |
| LTBP4 | Latent Transforming Growth Factor Beta Binding Protein 4 | Q8N2S1 |
| TBX5 | T-Box Transcription Factor 5 | Q99593 |
| EIF6 | Eukaryotic Translation Initiation Factor 6 | P56537 |
| TWIST2 | Twist Family BHLH Transcription Factor 2 | Q8WVJ9 |
| THY1 | Thy-1 Cell Surface Antigen | P04216 |
| GJB1 | Gap Junction Protein Beta 1 | P08034 |
| TPM3 | Tropomyosin 3 | P06753 |
| PRKCE | Protein Kinase C Epsilon | Q02156 |
| NFKBIB | NFKB Inhibitor Beta | Q15653 |
| CNTF | Ciliary Neurotrophic Factor | P26441 |
| CXADR | CXADR Ig-Like Cell Adhesion Molecule | P78310 |
| SNHG1 | Small Nucleolar RNA Host Gene 1 |  |
| ITGA4 | Integrin Subunit Alpha 4 | P13612 |
| EDNRB | Endothelin Receptor Type B | P24530 |
| NEU1 | Neuraminidase 1 | Q99519 |
| OSM | Oncostatin M | P13725 |
| PCYT1A | Phosphate Cytidylyltransferase 1A, Choline | P49585 |
| DRD2 | Dopamine Receptor D2 | P14416 |
| ALDH18A1 | Aldehyde Dehydrogenase 18 Family Member A1 | P54886 |
| SUZ12 | SUZ12 Polycomb Repressive Complex 2 Subunit | Q15022 |
| C3 | Complement C3 | P01024 |
| NPPC | Natriuretic Peptide C | P23582 |
| RPS28 | Ribosomal Protein S28 | P62857 |
| H1-4 | H1.4 Linker Histone, Cluster Member | P10412 |
| MIR451A | MicroRNA 451a |  |
| PRKAA1 | Protein Kinase AMP-Activated Catalytic Subunit Alpha 1 | Q13131 |
| CDH23 | Cadherin Related 23 | Q9H251 |
| GLA | Galactosidase Alpha | P06280 |
| HSD17B4 | Hydroxysteroid 17-Beta Dehydrogenase 4 | P51659 |
| SLC25A1 | Solute Carrier Family 25 Member 1 | P53007 |
| ACP1 | Acid Phosphatase 1 | P24666 |
| NFIB | Nuclear Factor I B | O00712 |
| NES | Nestin | P48681 |
| P4HA2 | Prolyl 4-Hydroxylase Subunit Alpha 2 | O15460 |
| FHL1 | Four And A Half LIM Domains 1 | Q13642 |
| SLC34A1 | Solute Carrier Family 34 Member 1 | Q06495 |
| IL9 | Interleukin 9 | P15248 |
| CCR2 | C-C Motif Chemokine Receptor 2 | P41597 |
| LIFR | LIF Receptor Subunit Alpha | P42702 |
| NR4A2 | Nuclear Receptor Subfamily 4 Group A Member 2 | P43354 |
| FGF14 | Fibroblast Growth Factor 14 | Q92915 |
| VIP | Vasoactive Intestinal Peptide | P01282 |
| F2R | Coagulation Factor II Thrombin Receptor | P25116 |
| ADAMTS5 | ADAM Metallopeptidase With Thrombospondin Type 1 Motif 5 | Q9UNA0 |
| FZD4 | Frizzled Class Receptor 4 | Q9ULV1 |
| LINC02882 | Long Intergenic Non-Protein Coding RNA 2882 |  |
| TOE1 | Target Of EGR1, Exonuclease | Q96GM8 |
| RPL35A | Ribosomal Protein L35a | P18077 |
| ADAMTS1 | ADAM Metallopeptidase With Thrombospondin Type 1 Motif 1 | Q9UHI8 |
| BCR | BCR Activator Of RhoGEF And GTPase | P11274 |
| HOXA2 | Homeobox A2 | O43364 |
| POMT1 | Protein O-Mannosyltransferase 1 | Q9Y6A1 |
| SH3PXD2B | SH3 And PX Domains 2B | A1X283 |
| IFT43 | Intraflagellar Transport 43 | Q96FT9 |
| OSTM1 | Osteoclastogenesis Associated Transmembrane Protein 1 | Q86WC4 |
| CAMP | Cathelicidin Antimicrobial Peptide | P49913 |
| AIF1 | Allograft Inflammatory Factor 1 | P55008 |
| AURKA | Aurora Kinase A | O14965 |
| FOXC1 | Forkhead Box C1 | Q12948 |
| PPBP | Pro-Platelet Basic Protein | P02775 |
| APTX | Aprataxin | Q7Z2E3 |
| CHI3L1 | Chitinase 3 Like 1 | P36222 |
| SGMS2 | Sphingomyelin Synthase 2 | Q8NHU3 |
| GC | GC Vitamin D Binding Protein | P02774 |
| ALDH1A1 | Aldehyde Dehydrogenase 1 Family Member A1 | P00352 |
| MIR379 | MicroRNA 379 |  |
| HTRA1 | HtrA Serine Peptidase 1 | Q92743 |
| WNT6 | Wnt Family Member 6 | Q9Y6F9 |
| LINC00504 | Long Intergenic Non-Protein Coding RNA 504 |  |
| ARF4-AS1 | ARF4 Antisense RNA 1 |  |
| LINC01258 | Long Intergenic Non-Protein Coding RNA 1258 |  |
| LINC02955 | Long Intergenic Non-Protein Coding RNA 2955 |  |
| ENSG00000230490 | Novel Transcript |  |
| ENSG00000245768 | Novel Transcript |  |
| ENSG00000250519 | Novel Transcript |  |
| ENSG00000258081 | Novel Transcript |  |
| ENSG00000235450 | Novel Transcript |  |
| ENSG00000251216 | Novel Transcript |  |
| ENSG00000253288 | Novel Transcript |  |
| lnc-IQCM-2 | |  |
| lnc-HMGXB4-8 | |  |
| MK280073-008 | |  |
| MK280073-007 | |  |
| MK280073-013 | |  |
| MK280073-001 | |  |
| MK280073-002 | |  |
| MK280073-003 | |  |
| MK280073-004 | |  |
| MK280073-005 | |  |
| MK280073-006 | |  |
| MK280073-009 | |  |
| MK280073-010 | |  |
| MK280073-011 | |  |
| MK280073-012 | |  |
| MK280073-014 | |  |
| MK280073-015 | |  |
| lnc-LRP5L-15 | |  |
| BICD2 | BICD Cargo Adaptor 2 | Q8TD16 |
| SUCLA2 | Succinate-CoA Ligase ADP-Forming Subunit Beta | Q9P2R7 |
| PDYN | Prodynorphin | P01213 |
| MIR330 | MicroRNA 330 |  |
| KCNJ2 | Potassium Inwardly Rectifying Channel Subfamily J Member 2 | P63252 |
| RBM8A | RNA Binding Motif Protein 8A | Q9Y5S9 |
| MIR200C | MicroRNA 200c |  |
| PARK7 | Parkinsonism Associated Deglycase | Q99497 |
| SNAP29 | Synaptosome Associated Protein 29 | O95721 |
| SUCLG1 | Succinate-CoA Ligase GDP/ADP-Forming Subunit Alpha | P53597 |
| PF4 | Platelet Factor 4 | P02776 |
| FGF13 | Fibroblast Growth Factor 13 | Q92913 |
| HNRNPR | Heterogeneous Nuclear Ribonucleoprotein R | O43390 |
| HDAC2 | Histone Deacetylase 2 | Q92769 |
| ADAMTS13 | ADAM Metallopeptidase With Thrombospondin Type 1 Motif 13 | Q76LX8 |
| NRG1 | Neuregulin 1 | Q02297 |
| SEMA3A | Semaphorin 3A | Q14563 |
| MYO5A | Myosin VA | Q9Y4I1 |
| FGB | Fibrinogen Beta Chain | P02675 |
| ANXA1 | Annexin A1 | P04083 |
| MIR199B | MicroRNA 199b |  |
| LRP8 | LDL Receptor Related Protein 8 | Q14114 |
| HNRNPH1 | Heterogeneous Nuclear Ribonucleoprotein H1 | P31943 |
| SGCG | Sarcoglycan Gamma | Q13326 |
| SPTBN1 | Spectrin Beta, Non-Erythrocytic 1 | Q01082 |
| HSPA1B | Heat Shock Protein Family A (Hsp70) Member 1B | P0DMV9 |
| IL17RA | Interleukin 17 Receptor A | Q96F46 |
| ABCC2 | ATP Binding Cassette Subfamily C Member 2 | Q92887 |
| GMPPB | GDP-Mannose Pyrophosphorylase B | Q9Y5P6 |
| ALG12 | ALG12 Alpha-1,6-Mannosyltransferase | Q9BV10 |
| KCNJ8 | Potassium Inwardly Rectifying Channel Subfamily J Member 8 | Q15842 |
| YY1 | YY1 Transcription Factor | P25490 |
| CX3CR1 | C-X3-C Motif Chemokine Receptor 1 | P49238 |
| MME | Membrane Metalloendopeptidase | P08473 |
| HPD | 4-Hydroxyphenylpyruvate Dioxygenase | P32754 |
| PTGER4 | Prostaglandin E Receptor 4 | P35408 |
| CAMK2A | Calcium/Calmodulin Dependent Protein Kinase II Alpha | Q9UQM7 |
| SLC1A2 | Solute Carrier Family 1 Member 2 | P43004 |
| COL6A2 | Collagen Type VI Alpha 2 Chain | P12110 |
| COG2 | Component Of Oligomeric Golgi Complex 2 | Q14746 |
| SUMF1 | Sulfatase Modifying Factor 1 | Q8NBK3 |
| MRE11 | MRE11 Homolog, Double Strand Break Repair Nuclease | P49959 |
| BMPR2 | Bone Morphogenetic Protein Receptor Type 2 | Q13873 |
| MPL | MPL Proto-Oncogene, Thrombopoietin Receptor | P40238 |
| DCC | DCC Netrin 1 Receptor | P43146 |
| GFM1 | G Elongation Factor Mitochondrial 1 | Q96RP9 |
| UFM1 | Ubiquitin Fold Modifier 1 | P61960 |
| G6PD | Glucose-6-Phosphate Dehydrogenase | P11413 |
| NRCAM | Neuronal Cell Adhesion Molecule | Q92823 |
| POMGNT2 | Protein O-Linked Mannose N-Acetylglucosaminyltransferase 2 (Beta 1,4-) | Q8NAT1 |
| CYP11A1 | Cytochrome P450 Family 11 Subfamily A Member 1 | P05108 |
| CD79A | CD79a Molecule | P11912 |
| MIR20B | MicroRNA 20b |  |
| CA2 | Carbonic Anhydrase 2 | P00918 |
| POLR1D | RNA Polymerase I And III Subunit D | P0DPB6 |
| GLS | Glutaminase | O94925 |
| CRYAA | Crystallin Alpha A | P02489 |
| ARID2 | AT-Rich Interaction Domain 2 | Q68CP9 |
| MIR216A | MicroRNA 216a |  |
| IAPP | Islet Amyloid Polypeptide | P10997 |
| MYF5 | Myogenic Factor 5 | P13349 |
| LOC106627981 | GBA Recombination Region |  |
| HNRNPA1 | Heterogeneous Nuclear Ribonucleoprotein A1 | P09651 |
| SMARCD1 | SWI/SNF Related, Matrix Associated, Actin Dependent Regulator Of Chromatin, Subfamily D, Member 1 | Q96GM5 |
| PLCE1 | Phospholipase C Epsilon 1 | Q9P212 |
| PTPN6 | Protein Tyrosine Phosphatase Non-Receptor Type 6 | P29350 |
| GAST | Gastrin | P01350 |
| HAPLN1 | Hyaluronan And Proteoglycan Link Protein 1 | P10915 |
| PROCR | Protein C Receptor | Q9UNN8 |
| SLC39A8 | Solute Carrier Family 39 Member 8 | Q9C0K1 |
| CYP2C9 | Cytochrome P450 Family 2 Subfamily C Member 9 | P11712 |
| MIR17HG | MiR-17-92a-1 Cluster Host Gene | Q75NE6 |
| MIR320A | MicroRNA 320a |  |
| SLC16A1 | Solute Carrier Family 16 Member 1 | P53985 |
| GORASP1 | Golgi Reassembly Stacking Protein 1 | Q9BQQ3 |
| BPI | Bactericidal Permeability Increasing Protein | P17213 |
| VCAN | Versican | P13611 |
| GRHL3 | Grainyhead Like Transcription Factor 3 | Q8TE85 |
| NEK1 | NIMA Related Kinase 1 | Q96PY6 |
| MCM4 | Minichromosome Maintenance Complex Component 4 | P33991 |
| PLCB4 | Phospholipase C Beta 4 | Q15147 |
| CDX2 | Caudal Type Homeobox 2 | Q99626 |
| AMER1 | APC Membrane Recruitment Protein 1 | Q5JTC6 |
| DLL4 | Delta Like Canonical Notch Ligand 4 | Q9NR61 |
| GBA2 | Glucosylceramidase Beta 2 | Q9HCG7 |
| OXT | Oxytocin/Neurophysin I Prepropeptide | P01178 |
| GRM7 | Glutamate Metabotropic Receptor 7 | Q14831 |
| FABP4 | Fatty Acid Binding Protein 4 | P15090 |
| CALCR | Calcitonin Receptor | P30988 |
| ATP7B | ATPase Copper Transporting Beta | P35670 |
| MIR206 | MicroRNA 206 |  |
| GPNMB | Glycoprotein Nmb | Q14956 |
| ERCC3 | ERCC Excision Repair 3, TFIIH Core Complex Helicase Subunit | P19447 |
| PCSK9 | Proprotein Convertase Subtilisin/Kexin Type 9 | Q8NBP7 |
| TRPV6 | Transient Receptor Potential Cation Channel Subfamily V Member 6 | Q9H1D0 |
| RUNX3 | RUNX Family Transcription Factor 3 | Q13761 |
| SLC39A4 | Solute Carrier Family 39 Member 4 | Q6P5W5 |
| CYGB | Cytoglobin | Q8WWM9 |
| A2M | Alpha-2-Macroglobulin | P01023 |
| S100A8 | S100 Calcium Binding Protein A8 | P05109 |
| ZNF469 | Zinc Finger Protein 469 | Q96JG9 |
| HEY2 | Hes Related Family BHLH Transcription Factor With YRPW Motif 2 | Q9UBP5 |
| S100A1 | S100 Calcium Binding Protein A1 | P23297 |
| HSD11B2 | Hydroxysteroid 11-Beta Dehydrogenase 2 | P80365 |
| GNRHR | Gonadotropin Releasing Hormone Receptor | P30968 |
| ADAMTS3 | ADAM Metallopeptidase With Thrombospondin Type 1 Motif 3 | O15072 |
| TMC8 | Transmembrane Channel Like 8 | Q8IU68 |
| EIF2S1 | Eukaryotic Translation Initiation Factor 2 Subunit Alpha | P05198 |
| AAAS | Aladin WD Repeat Nucleoporin | Q9NRG9 |
| LOC102724058 | Uncharacterized LOC102724058 |  |
| DBH | Dopamine Beta-Hydroxylase | P09172 |
| LIPC | Lipase C, Hepatic Type | P11150 |
| CTSL | Cathepsin L | P07711 |
| TUBA8 | Tubulin Alpha 8 | Q9NY65 |
| EYA1 | EYA Transcriptional Coactivator And Phosphatase 1 | Q99502 |
| ANXA2 | Annexin A2 | P07355 |
| CSMD1 | CUB And Sushi Multiple Domains 1 | Q96PZ7 |
| PKD2 | Polycystin 2, Transient Receptor Potential Cation Channel | Q13563 |
| CYB5A | Cytochrome B5 Type A | P00167 |
| FGFRL1 | Fibroblast Growth Factor Receptor Like 1 | Q8N441 |
| DACT1 | Dishevelled Binding Antagonist Of Beta Catenin 1 | Q9NYF0 |
| CXCL2 | C-X-C Motif Chemokine Ligand 2 | P19875 |
| ABCC3 | ATP Binding Cassette Subfamily C Member 3 | O15438 |
| ANG | Angiogenin | P03950 |
| DNASE1L3 | Deoxyribonuclease 1 Like 3 | Q13609 |
| UGT1A1 | UDP Glucuronosyltransferase Family 1 Member A1 | P22309 |
| NCOA3 | Nuclear Receptor Coactivator 3 | Q9Y6Q9 |
| CDH11 | Cadherin 11 | P55287 |
| MRPS34 | Mitochondrial Ribosomal Protein S34 | P82930 |
| ITGA1 | Integrin Subunit Alpha 1 | P56199 |
| MIR31HG | MIR31 Host Gene |  |
| HNF1A | HNF1 Homeobox A | P20823 |
| PPARD | Peroxisome Proliferator Activated Receptor Delta | Q03181 |
| PROM1 | Prominin 1 | O43490 |
| CHD2 | Chromodomain Helicase DNA Binding Protein 2 | O14647 |
| LGI1 | Leucine Rich Glioma Inactivated 1 | O95970 |
| DYNLL1 | Dynein Light Chain LC8-Type 1 | P63167 |
| PUF60 | Poly(U) Binding Splicing Factor 60 | Q9UHX1 |
| MSTN | Myostatin | O14793 |
| CACNA1C | Calcium Voltage-Gated Channel Subunit Alpha1 C | Q13936 |
| TNNT2 | Troponin T2, Cardiac Type | P45379 |
| RAB3GAP1 | RAB3 GTPase Activating Protein Catalytic Subunit 1 | Q15042 |
| TTTY10 | Testis Expressed Transcript, Y-Linked 10 | Q9BZA0 |
| MK280073-023 | |  |
| MK280073-058 | |  |
| MK280073-197 | |  |
| MK280073-346 | |  |
| MK280073-501 | |  |
| MK280073-020 | |  |
| MK280073-022 | |  |
| MK280073-049 | |  |
| MK280073-055 | |  |
| MK280073-060 | |  |
| MK280073-063 | |  |
| MK280073-083 | |  |
| MK280073-093 | |  |
| MK280073-120 | |  |
| MK280073-121 | |  |
| MK280073-164 | |  |
| MK280073-202 | |  |
| MK280073-300 | |  |
| MK280073-334 | |  |
| MK280073-475 | |  |
| MK280073-493 | |  |
| MK280073-510 | |  |
| MK280073-521 | |  |
| MK280073-571 | |  |
| MK280073-623 | |  |
| MK280073-018 | |  |
| MK280073-025 | |  |
| MK280073-027 | |  |
| MK280073-028 | |  |
| MK280073-030 | |  |
| MK280073-040 | |  |
| MK280073-044 | |  |
| MK280073-052 | |  |
| MK280073-059 | |  |
| MK280073-072 | |  |
| MK280073-081 | |  |
| MK280073-082 | |  |
| MK280073-090 | |  |
| MK280073-091 | |  |
| MK280073-115 | |  |
| MK280073-117 | |  |
| MK280073-123 | |  |
| MK280073-140 | |  |
| MK280073-149 | |  |
| MK280073-150 | |  |
| MK280073-153 | |  |
| MK280073-175 | |  |
| MK280073-176 | |  |
| MK280073-180 | |  |
| MK280073-182 | |  |
| MK280073-199 | |  |
| MK280073-203 | |  |
| MK280073-205 | |  |
| MK280073-206 | |  |
| MK280073-207 | |  |
| MK280073-216 | |  |
| MK280073-243 | |  |
| MK280073-273 | |  |
| MK280073-283 | |  |
| MK280073-295 | |  |
| MK280073-296 | |  |
| MK280073-351 | |  |
| MK280073-353 | |  |
| MK280073-354 | |  |
| MK280073-359 | |  |
| MK280073-389 | |  |
| MK280073-453 | |  |
| MK280073-456 | |  |
| MK280073-460 | |  |
| MK280073-461 | |  |
| MK280073-464 | |  |
| MK280073-468 | |  |
| MK280073-470 | |  |
| MK280073-473 | |  |
| MK280073-490 | |  |
| MK280073-513 | |  |
| MK280073-519 | |  |
| MK280073-522 | |  |
| MK280073-523 | |  |
| MK280073-524 | |  |
| MK280073-525 | |  |
| MK280073-527 | |  |
| MK280073-533 | |  |
| MK280073-544 | |  |
| MK280073-557 | |  |
| MK280073-561 | |  |
| MK280073-586 | |  |
| MK280073-609 | |  |
| MK280073-621 | |  |
| MK280073-016 | |  |
| MK280073-017 | |  |
| MK280073-019 | |  |
| MK280073-021 | |  |
| MK280073-024 | |  |
| MK280073-026 | |  |
| MK280073-029 | |  |
| MK280073-031 | |  |
| MK280073-032 | |  |
| MK280073-033 | |  |
| MK280073-034 | |  |
| MK280073-035 | |  |
| MK280073-036 | |  |
| MK280073-037 | |  |
| MK280073-038 | |  |
| MK280073-039 | |  |
| MK280073-041 | |  |
| MK280073-042 | |  |
| MK280073-043 | |  |
| MK280073-045 | |  |
| MK280073-046 | |  |
| MK280073-047 | |  |
| MK280073-048 | |  |
| MK280073-050 | |  |
| MK280073-051 | |  |
| MK280073-053 | |  |
| MK280073-054 | |  |
| MK280073-056 | |  |
| MK280073-057 | |  |
| MK280073-061 | |  |
| MK280073-062 | |  |
| MK280073-064 | |  |
| MK280073-065 | |  |
| MK280073-066 | |  |
| MK280073-068 | |  |
| MK280073-069 | |  |
| MK280073-070 | |  |
| MK280073-071 | |  |
| MK280073-073 | |  |
| MK280073-074 | |  |
| MK280073-075 | |  |
| MK280073-076 | |  |
| MK280073-077 | |  |
| MK280073-078 | |  |
| MK280073-079 | |  |
| MK280073-080 | |  |
| MK280073-084 | |  |
| MK280073-085 | |  |
| MK280073-086 | |  |
| MK280073-087 | |  |
| MK280073-088 | |  |
| MK280073-089 | |  |
| MK280073-092 | |  |
| MK280073-094 | |  |
| MK280073-095 | |  |
| MK280073-096 | |  |
| MK280073-097 | |  |
| MK280073-098 | |  |
| MK280073-101 | |  |
| MK280073-102 | |  |
| MK280073-103 | |  |
| MK280073-104 | |  |
| MK280073-105 | |  |
| MK280073-106 | |  |
| MK280073-107 | |  |
| MK280073-108 | |  |
| MK280073-109 | |  |
| MK280073-110 | |  |
| MK280073-111 | |  |
| MK280073-112 | |  |
| MK280073-113 | |  |
| MK280073-114 | |  |
| MK280073-116 | |  |
| MK280073-118 | |  |
| MK280073-119 | |  |
| MK280073-122 | |  |
| MK280073-124 | |  |
| MK280073-125 | |  |
| MK280073-126 | |  |
| MK280073-127 | |  |
| MK280073-128 | |  |
| MK280073-131 | |  |
| MK280073-132 | |  |
| MK280073-133 | |  |
| MK280073-134 | |  |
| MK280073-135 | |  |
| MK280073-136 | |  |
| MK280073-137 | |  |
| MK280073-138 | |  |
| MK280073-139 | |  |
| MK280073-141 | |  |
| MK280073-142 | |  |
| MK280073-143 | |  |
| MK280073-144 | |  |
| MK280073-145 | |  |
| MK280073-146 | |  |
| MK280073-147 | |  |
| MK280073-148 | |  |
| MK280073-151 | |  |
| MK280073-152 | |  |
| MK280073-154 | |  |
| MK280073-155 | |  |
| MK280073-156 | |  |
| MK280073-157 | |  |
| MK280073-158 | |  |
| MK280073-159 | |  |
| MK280073-160 | |  |
| MK280073-161 | |  |
| MK280073-162 | |  |
| MK280073-163 | |  |
| MK280073-165 | |  |
| MK280073-168 | |  |
| MK280073-169 | |  |
| MK280073-170 | |  |
| MK280073-171 | |  |
| MK280073-172 | |  |
| MK280073-173 | |  |
| MK280073-174 | |  |
| MK280073-177 | |  |
| MK280073-178 | |  |
| MK280073-179 | |  |
| MK280073-181 | |  |
| MK280073-183 | |  |
| MK280073-184 | |  |
| MK280073-185 | |  |
| MK280073-186 | |  |
| MK280073-187 | |  |
| MK280073-188 | |  |
| MK280073-189 | |  |
| MK280073-190 | |  |
| MK280073-191 | |  |
| MK280073-192 | |  |
| MK280073-193 | |  |
| MK280073-194 | |  |
| MK280073-195 | |  |
| MK280073-196 | |  |
| MK280073-198 | |  |
| MK280073-200 | |  |
| MK280073-201 | |  |
| MK280073-204 | |  |
| MK280073-208 | |  |
| MK280073-209 | |  |
| MK280073-210 | |  |
| MK280073-211 | |  |
| MK280073-212 | |  |
| MK280073-213 | |  |
| MK280073-214 | |  |
| MK280073-215 | |  |
| MK280073-217 | |  |
| MK280073-218 | |  |
| MK280073-219 | |  |
| MK280073-220 | |  |
| MK280073-221 | |  |
| MK280073-222 | |  |
| MK280073-224 | |  |
| MK280073-225 | |  |
| MK280073-226 | |  |
| MK280073-227 | |  |
| MK280073-228 | |  |
| MK280073-229 | |  |
| MK280073-230 | |  |
| MK280073-231 | |  |
| MK280073-232 | |  |
| MK280073-233 | |  |
| MK280073-234 | |  |
| MK280073-235 | |  |
| MK280073-236 | |  |
| MK280073-237 | |  |
| MK280073-238 | |  |
| MK280073-239 | |  |
| MK280073-240 | |  |
| MK280073-241 | |  |
| MK280073-242 | |  |
| MK280073-244 | |  |
| MK280073-245 | |  |
| MK280073-246 | |  |
| MK280073-247 | |  |
| MK280073-248 | |  |
| MK280073-249 | |  |
| MK280073-250 | |  |
| MK280073-251 | |  |
| MK280073-252 | |  |
| MK280073-253 | |  |
| MK280073-255 | |  |
| MK280073-257 | |  |
| MK280073-258 | |  |
| MK280073-259 | |  |
| MK280073-260 | |  |
| MK280073-261 | |  |
| MK280073-262 | |  |
| MK280073-264 | |  |
| MK280073-265 | |  |
| MK280073-266 | |  |
| MK280073-267 | |  |
| MK280073-268 | |  |
| MK280073-269 | |  |
| MK280073-270 | |  |
| MK280073-271 | |  |
| MK280073-272 | |  |
| MK280073-274 | |  |
| MK280073-275 | |  |
| MK280073-276 | |  |
| MK280073-277 | |  |
| MK280073-278 | |  |
| MK280073-279 | |  |
| MK280073-280 | |  |
| MK280073-281 | |  |
| MK280073-282 | |  |
| MK280073-284 | |  |
| MK280073-285 | |  |
| MK280073-286 | |  |
| MK280073-287 | |  |
| MK280073-288 | |  |
| MK280073-289 | |  |
| MK280073-290 | |  |
| MK280073-291 | |  |
| MK280073-292 | |  |
| MK280073-293 | |  |
| MK280073-294 | |  |
| MK280073-297 | |  |
| MK280073-298 | |  |
| MK280073-299 | |  |
| MK280073-301 | |  |
| MK280073-302 | |  |
| MK280073-303 | |  |
| MK280073-304 | |  |
| MK280073-305 | |  |
| MK280073-306 | |  |
| MK280073-307 | |  |
| MK280073-308 | |  |
| MK280073-309 | |  |
| MK280073-310 | |  |
| MK280073-311 | |  |
| MK280073-312 | |  |
| MK280073-313 | |  |
| MK280073-314 | |  |
| MK280073-315 | |  |
| MK280073-316 | |  |
| MK280073-317 | |  |
| MK280073-318 | |  |
| MK280073-319 | |  |
| MK280073-320 | |  |
| MK280073-321 | |  |
| MK280073-322 | |  |
| MK280073-323 | |  |
| MK280073-324 | |  |
| MK280073-325 | |  |
| MK280073-326 | |  |
| MK280073-328 | |  |
| MK280073-329 | |  |
| MK280073-330 | |  |
| MK280073-331 | |  |
| MK280073-332 | |  |
| MK280073-333 | |  |
| MK280073-335 | |  |
| MK280073-336 | |  |
| MK280073-337 | |  |
| MK280073-338 | |  |
| MK280073-339 | |  |
| MK280073-340 | |  |
| MK280073-341 | |  |
| MK280073-342 | |  |
| MK280073-343 | |  |
| MK280073-344 | |  |
| MK280073-345 | |  |
| MK280073-347 | |  |
| MK280073-348 | |  |
| MK280073-349 | |  |
| MK280073-350 | |  |
| MK280073-352 | |  |
| MK280073-355 | |  |
| MK280073-356 | |  |
| MK280073-357 | |  |
| MK280073-358 | |  |
| MK280073-360 | |  |
| MK280073-361 | |  |
| MK280073-362 | |  |
| MK280073-363 | |  |
| MK280073-364 | |  |
| MK280073-365 | |  |
| MK280073-366 | |  |
| MK280073-367 | |  |
| MK280073-368 | |  |
| MK280073-369 | |  |
| MK280073-370 | |  |
| MK280073-371 | |  |
| MK280073-372 | |  |
| MK280073-373 | |  |
| MK280073-374 | |  |
| MK280073-375 | |  |
| MK280073-376 | |  |
| MK280073-377 | |  |
| MK280073-378 | |  |
| MK280073-379 | |  |
| MK280073-380 | |  |
| MK280073-381 | |  |
| MK280073-382 | |  |
| MK280073-383 | |  |
| MK280073-384 | |  |
| MK280073-385 | |  |
| MK280073-386 | |  |
| MK280073-387 | |  |
| MK280073-388 | |  |
| MK280073-390 | |  |
| MK280073-391 | |  |
| MK280073-395 | |  |
| MK280073-396 | |  |
| MK280073-397 | |  |
| MK280073-398 | |  |
| MK280073-399 | |  |
| MK280073-402 | |  |
| MK280073-403 | |  |
| MK280073-404 | |  |
| MK280073-405 | |  |
| MK280073-406 | |  |
| MK280073-407 | |  |
| MK280073-409 | |  |
| MK280073-410 | |  |
| MK280073-411 | |  |
| MK280073-412 | |  |
| MK280073-413 | |  |
| MK280073-414 | |  |
| MK280073-415 | |  |
| MK280073-416 | |  |
| MK280073-417 | |  |
| MK280073-418 | |  |
| MK280073-419 | |  |
| MK280073-420 | |  |
| MK280073-427 | |  |
| MK280073-428 | |  |
| MK280073-430 | |  |
| MK280073-431 | |  |
| MK280073-432 | |  |
| MK280073-433 | |  |
| MK280073-434 | |  |
| MK280073-435 | |  |
| MK280073-436 | |  |
| MK280073-437 | |  |
| MK280073-438 | |  |
| MK280073-439 | |  |
| MK280073-440 | |  |
| MK280073-441 | |  |
| MK280073-442 | |  |
| MK280073-443 | |  |
| MK280073-444 | |  |
| MK280073-445 | |  |
| MK280073-446 | |  |
| MK280073-447 | |  |
| MK280073-448 | |  |
| MK280073-449 | |  |
| MK280073-450 | |  |
| MK280073-451 | |  |
| MK280073-452 | |  |
| MK280073-454 | |  |
| MK280073-455 | |  |
| MK280073-457 | |  |
| MK280073-458 | |  |
| MK280073-459 | |  |
| MK280073-462 | |  |
| MK280073-463 | |  |
| MK280073-465 | |  |
| MK280073-466 | |  |
| MK280073-467 | |  |
| MK280073-469 | |  |
| MK280073-471 | |  |
| MK280073-472 | |  |
| MK280073-474 | |  |
| MK280073-476 | |  |
| MK280073-477 | |  |
| MK280073-478 | |  |
| MK280073-479 | |  |
| MK280073-480 | |  |
| MK280073-481 | |  |
| MK280073-482 | |  |
| MK280073-483 | |  |
| MK280073-484 | |  |
| MK280073-485 | |  |
| MK280073-486 | |  |
| MK280073-487 | |  |
| MK280073-488 | |  |
| MK280073-491 | |  |
| MK280073-492 | |  |
| MK280073-494 | |  |
| MK280073-495 | |  |
| MK280073-496 | |  |
| MK280073-497 | |  |
| MK280073-498 | |  |
| MK280073-499 | |  |
| MK280073-500 | |  |
| MK280073-502 | |  |
| MK280073-503 | |  |
| MK280073-504 | |  |
| MK280073-505 | |  |
| MK280073-506 | |  |
| MK280073-507 | |  |
| MK280073-508 | |  |
| MK280073-509 | |  |
| MK280073-511 | |  |
| MK280073-512 | |  |
| MK280073-514 | |  |
| MK280073-515 | |  |
| MK280073-516 | |  |
| MK280073-517 | |  |
| MK280073-518 | |  |
| MK280073-526 | |  |
| MK280073-528 | |  |
| MK280073-529 | |  |
| MK280073-530 | |  |
| MK280073-531 | |  |
| MK280073-532 | |  |
| MK280073-534 | |  |
| MK280073-535 | |  |
| MK280073-536 | |  |
| MK280073-537 | |  |
| MK280073-538 | |  |
| MK280073-540 | |  |
| MK280073-541 | |  |
| MK280073-542 | |  |
| MK280073-543 | |  |
| MK280073-545 | |  |
| MK280073-546 | |  |
| MK280073-547 | |  |
| MK280073-548 | |  |
| MK280073-549 | |  |
| MK280073-550 | |  |
| MK280073-551 | |  |
| MK280073-552 | |  |
| MK280073-553 | |  |
| MK280073-554 | |  |
| MK280073-555 | |  |
| MK280073-556 | |  |
| MK280073-558 | |  |
| MK280073-559 | |  |
| MK280073-560 | |  |
| MK280073-562 | |  |
| MK280073-563 | |  |
| MK280073-564 | |  |
| MK280073-565 | |  |
| MK280073-566 | |  |
| MK280073-567 | |  |
| MK280073-568 | |  |
| MK280073-569 | |  |
| MK280073-570 | |  |
| MK280073-572 | |  |
| MK280073-573 | |  |
| MK280073-574 | |  |
| MK280073-575 | |  |
| MK280073-577 | |  |
| MK280073-578 | |  |
| MK280073-579 | |  |
| MK280073-580 | |  |
| MK280073-581 | |  |
| MK280073-582 | |  |
| MK280073-583 | |  |
| MK280073-584 | |  |
| MK280073-585 | |  |
| MK280073-588 | |  |
| MK280073-589 | |  |
| MK280073-590 | |  |
| MK280073-591 | |  |
| MK280073-592 | |  |
| MK280073-593 | |  |
| MK280073-594 | |  |
| MK280073-595 | |  |
| MK280073-596 | |  |
| MK280073-597 | |  |
| MK280073-598 | |  |
| MK280073-599 | |  |
| MK280073-600 | |  |
| MK280073-606 | |  |
| MK280073-615 | |  |
| MK280073-618 | |  |
| MK280073-624 | |  |
| MK280073-625 | |  |
| MK280073-629 | |  |
| MK280073-654 | |  |
| MK280073-655 | |  |
| MK280073-656 | |  |
| MK280073-659 | |  |
| MK280073-670 | |  |
| MK280073-674 | |  |
| MK280073-676 | |  |
| MK280073-678 | |  |
| MK280073-683 | |  |
| MK280073-686 | |  |
| MK280073-688 | |  |
| MK280073-689 | |  |
| MK280073-691 | |  |
| MK280073-067 | |  |
| MK280073-099 | |  |
| MK280073-100 | |  |
| MK280073-129 | |  |
| MK280073-130 | |  |
| MK280073-166 | |  |
| MK280073-167 | |  |
| MK280073-223 | |  |
| MK280073-254 | |  |
| MK280073-256 | |  |
| MK280073-263 | |  |
| MK280073-327 | |  |
| MK280073-392 | |  |
| MK280073-393 | |  |
| MK280073-394 | |  |
| MK280073-400 | |  |
| MK280073-401 | |  |
| MK280073-408 | |  |
| MK280073-421 | |  |
| MK280073-422 | |  |
| MK280073-423 | |  |
| MK280073-424 | |  |
| MK280073-425 | |  |
| MK280073-426 | |  |
| MK280073-429 | |  |
| MK280073-489 | |  |
| MK280073-520 | |  |
| MK280073-539 | |  |
| MK280073-576 | |  |
| MK280073-587 | |  |
| MK280073-601 | |  |
| MK280073-602 | |  |
| MK280073-603 | |  |
| MK280073-604 | |  |
| MK280073-605 | |  |
| MK280073-607 | |  |
| MK280073-608 | |  |
| MK280073-610 | |  |
| MK280073-611 | |  |
| MK280073-612 | |  |
| MK280073-613 | |  |
| MK280073-614 | |  |
| MK280073-616 | |  |
| MK280073-617 | |  |
| MK280073-619 | |  |
| MK280073-620 | |  |
| MK280073-622 | |  |
| MK280073-626 | |  |
| MK280073-627 | |  |
| MK280073-628 | |  |
| MK280073-630 | |  |
| MK280073-631 | |  |
| MK280073-632 | |  |
| MK280073-633 | |  |
| MK280073-634 | |  |
| MK280073-635 | |  |
| MK280073-636 | |  |
| MK280073-637 | |  |
| MK280073-638 | |  |
| MK280073-639 | |  |
| MK280073-640 | |  |
| MK280073-641 | |  |
| MK280073-642 | |  |
| MK280073-643 | |  |
| MK280073-644 | |  |
| MK280073-645 | |  |
| MK280073-646 | |  |
| MK280073-647 | |  |
| MK280073-648 | |  |
| MK280073-649 | |  |
| MK280073-650 | |  |
| MK280073-651 | |  |
| MK280073-652 | |  |
| MK280073-653 | |  |
| MK280073-657 | |  |
| MK280073-658 | |  |
| MK280073-660 | |  |
| MK280073-661 | |  |
| MK280073-662 | |  |
| MK280073-663 | |  |
| MK280073-664 | |  |
| MK280073-665 | |  |
| MK280073-666 | |  |
| MK280073-667 | |  |
| MK280073-668 | |  |
| MK280073-669 | |  |
| MK280073-671 | |  |
| MK280073-672 | |  |
| MK280073-673 | |  |
| MK280073-675 | |  |
| MK280073-677 | |  |
| MK280073-679 | |  |
| MK280073-680 | |  |
| MK280073-681 | |  |
| MK280073-682 | |  |
| MK280073-684 | |  |
| MK280073-685 | |  |
| MK280073-687 | |  |
| MK280073-690 | |  |
| MK280073-692 | |  |
| MK280073-693 | |  |
| MK280073-694 | |  |
| MK280073-695 | |  |
| MK280073-696 | |  |
| MK280073-697 | |  |
| MK280073-698 | |  |
| MK280073-699 | |  |
| MK280073-700 | |  |
| MK280073-701 | |  |
| MK280073-702 | |  |
| MK280073-703 | |  |
| MK280073-704 | |  |
| MK280073-705 | |  |
| MK280073-706 | |  |
| MK280073-707 | |  |
| MK280073-708 | |  |
| MK280073-709 | |  |
| MK280073-710 | |  |
| MK280073-711 | |  |
| MK280073-712 | |  |
| MK280073-713 | |  |
| MK280073-714 | |  |
| MK280073-715 | |  |
| MK280073-716 | |  |
| MK280073-717 | |  |
| MK280073-718 | |  |
| MK280073-719 | |  |
| MK280073-720 | |  |
| MK280073-721 | |  |
| MK280073-722 | |  |
| DPH2 | Diphthamide Biosynthesis 2 | Q9BQC3 |
| GP1BA | Glycoprotein Ib Platelet Subunit Alpha | P07359 |
| RELB | RELB Proto-Oncogene, NF-KB Subunit | Q01201 |
| IRX1 | Iroquois Homeobox 1 | P78414 |
| FGF10 | Fibroblast Growth Factor 10 | O15520 |
| LINC-PINT | Long Intergenic Non-Protein Coding RNA, P53 Induced Transcript | A0A455ZAR2 |
| ARSA | Arylsulfatase A | P15289 |
| AHCY | Adenosylhomocysteinase | P23526 |
| GHRH | Growth Hormone Releasing Hormone | P01286 |
| P2RX3 | Purinergic Receptor P2X 3 | P56373 |
| ADRB2 | Adrenoceptor Beta 2 | P07550 |
| IGHMBP2 | Immunoglobulin Mu DNA Binding Protein 2 | P38935 |
| GOLGA2 | Golgin A2 | Q08379 |
| DDB2 | Damage Specific DNA Binding Protein 2 | Q92466 |
| SPTBN2 | Spectrin Beta, Non-Erythrocytic 2 | O15020 |
| C2CD3 | C2 Domain Containing 3 Centriole Elongation Regulator | Q4AC94 |
| CYP11B2 | Cytochrome P450 Family 11 Subfamily B Member 2 | P19099 |
| ABCD1 | ATP Binding Cassette Subfamily D Member 1 | P33897 |
| GOLPH3 | Golgi Phosphoprotein 3 | Q9H4A6 |
| MIR339 | MicroRNA 339 |  |
| CHRD | Chordin | Q9H2X0 |
| HK2 | Hexokinase 2 | P52789 |
| MIPEP | Mitochondrial Intermediate Peptidase | Q99797 |
| CCN1 | Cellular Communication Network Factor 1 | O00622 |
| SULF1 | Sulfatase 1 | Q8IWU6 |
| CCDC47 | Coiled-Coil Domain Containing 47 | Q96A33 |
| TACR1 | Tachykinin Receptor 1 | P25103 |
| GDI1 | GDP Dissociation Inhibitor 1 | P31150 |
| COQ8A | Coenzyme Q8A | Q8NI60 |
| ITGB6 | Integrin Subunit Beta 6 | P18564 |
| NID1 | Nidogen 1 | P14543 |
| HDAC3 | Histone Deacetylase 3 | O15379 |
| LINC01554 | Long Intergenic Non-Protein Coding RNA 1554 | Q52M75 |
| FGF4 | Fibroblast Growth Factor 4 | P08620 |
| LINC01370 | Long Intergenic Non-Protein Coding RNA 1370 |  |
| SAG | S-Antigen Visual Arrestin | P10523 |
| S100A6 | S100 Calcium Binding Protein A6 | P06703 |
| KCNMA1 | Potassium Calcium-Activated Channel Subfamily M Alpha 1 | Q12791 |
| ISG15 | ISG15 Ubiquitin Like Modifier | P05161 |
| NPHS2 | NPHS2 Stomatin Family Member, Podocin | Q9NP85 |
| SKIC2 | SKI2 Subunit Of Superkiller Complex | Q15477 |
| AZU1 | Azurocidin 1 | P20160 |
| EHMT2 | Euchromatic Histone Lysine Methyltransferase 2 | Q96KQ7 |
| NME1 | NME/NM23 Nucleoside Diphosphate Kinase 1 | P15531 |
| IRF2 | Interferon Regulatory Factor 2 | P14316 |
| CYP24A1 | Cytochrome P450 Family 24 Subfamily A Member 1 | Q07973 |
| ADORA2B | Adenosine A2b Receptor | P29275 |
| PLA2G2A | Phospholipase A2 Group IIA | P14555 |
| PEX1 | Peroxisomal Biogenesis Factor 1 | O43933 |
| ASS1 | Argininosuccinate Synthase 1 | P00966 |
| KIAA0753 | KIAA0753 | Q2KHM9 |
| FAT4 | FAT Atypical Cadherin 4 | Q6V0I7 |
| CSN1S1 | Casein Alpha S1 | P47710 |
| NR5A1 | Nuclear Receptor Subfamily 5 Group A Member 1 | Q13285 |
| NTF3 | Neurotrophin 3 | P20783 |
| CACNA2D1 | Calcium Voltage-Gated Channel Auxiliary Subunit Alpha2delta 1 | P54289 |
| ARF3 | ADP Ribosylation Factor 3 | P61204 |
| TRAPPC12 | Trafficking Protein Particle Complex Subunit 12 | Q8WVT3 |
| OTUD5 | OTU Deubiquitinase 5 | Q96G74 |
| TGM2 | Transglutaminase 2 | P21980 |
| HSP90B1 | Heat Shock Protein 90 Beta Family Member 1 | P14625 |
| ADCY10 | Adenylate Cyclase 10 | Q96PN6 |
| F8 | Coagulation Factor VIII | P00451 |
| USF3 | Upstream Transcription Factor Family Member 3 | Q68DE3 |
| BDKRB2 | Bradykinin Receptor B2 | P30411 |
| IRAK4 | Interleukin 1 Receptor Associated Kinase 4 | Q9NWZ3 |
| DKK3 | Dickkopf WNT Signaling Pathway Inhibitor 3 | Q9UBP4 |
| NEFH | Neurofilament Heavy Chain | P12036 |
| DPF2 | Double PHD Fingers 2 | Q92785 |
| TREM2 | Triggering Receptor Expressed On Myeloid Cells 2 | Q9NZC2 |
| SMPD3 | Sphingomyelin Phosphodiesterase 3 | Q9NY59 |
| ABCG5 | ATP Binding Cassette Subfamily G Member 5 | Q9H222 |
| CHIT1 | Chitinase 1 | Q13231 |
| LAMP1 | Lysosomal Associated Membrane Protein 1 | P11279 |
| RARG | Retinoic Acid Receptor Gamma | P13631 |
| GRIN1 | Glutamate Ionotropic Receptor NMDA Type Subunit 1 | Q05586 |
| MUS81 | MUS81 Structure-Specific Endonuclease Subunit | Q96NY9 |
| ATP2B1 | ATPase Plasma Membrane Ca2+ Transporting 1 | P20020 |
| GFRA1 | GDNF Family Receptor Alpha 1 | P56159 |
| WLS | Wnt Ligand Secretion Mediator | Q5T9L3 |
| PCAT2 | Prostate Cancer Associated Transcript 2 |  |
| CNR2 | Cannabinoid Receptor 2 | P34972 |
| CD55 | CD55 Molecule (Cromer Blood Group) | P08174 |
| MTAP | Methylthioadenosine Phosphorylase | Q13126 |
| AMBP | Alpha-1-Microglobulin/Bikunin Precursor | P02760 |
| WNT4 | Wnt Family Member 4 | P56705 |
| SOAT1 | Sterol O-Acyltransferase 1 | P35610 |
| CRHR1 | Corticotropin Releasing Hormone Receptor 1 | P34998 |
| PRPF6 | Pre-MRNA Processing Factor 6 | O94906 |
| SASH1 | SAM And SH3 Domain Containing 1 | O94885 |
| ITGA2B | Integrin Subunit Alpha 2b | P08514 |
| CTNNA2 | Catenin Alpha 2 | P26232 |
| CHKB | Choline Kinase Beta | Q9Y259 |
| PAX8 | Paired Box 8 | Q06710 |
| CKB | Creatine Kinase B | P12277 |
| NLRP1 | NLR Family Pyrin Domain Containing 1 | Q9C000 |
| SERPINB5 | Serpin Family B Member 5 | P36952 |
| PRKCB | Protein Kinase C Beta | P05771 |
| UGCG | UDP-Glucose Ceramide Glucosyltransferase | Q16739 |
| SMPD2 | Sphingomyelin Phosphodiesterase 2 | O60906 |
| IL17F | Interleukin 17F | Q96PD4 |
| SCARB1 | Scavenger Receptor Class B Member 1 | Q8WTV0 |
| CNTNAP2 | Contactin Associated Protein 2 | Q9UHC6 |
| PRSS1 | Serine Protease 1 | P07477 |
| PACRG | Parkin Coregulated | Q96M98 |
| MIR27B | MicroRNA 27b |  |
| CDK5RAP3 | CDK5 Regulatory Subunit Associated Protein 3 | Q96JB5 |
| ENAM | Enamelin | Q9NRM1 |
| ALX1 | ALX Homeobox 1 | Q15699 |
| ABCG8 | ATP Binding Cassette Subfamily G Member 8 | Q9H221 |
| ID1 | Inhibitor Of DNA Binding 1 | P41134 |
| STAR | Steroidogenic Acute Regulatory Protein | P49675 |
| RAB4B-EGLN2 | RAB4B-EGLN2 Readthrough (NMD Candidate) |  |
| RPS14 | Ribosomal Protein S14 | P62263 |
| BMI1 | BMI1 Proto-Oncogene, Polycomb Ring Finger | P35226 |
| MMP20 | Matrix Metallopeptidase 20 | O60882 |
| NTRK3 | Neurotrophic Receptor Tyrosine Kinase 3 | Q16288 |
| GPX1 | Glutathione Peroxidase 1 | P07203 |
| FBLN1 | Fibulin 1 | P23142 |
| IRAK3 | Interleukin 1 Receptor Associated Kinase 3 | Q9Y616 |
| DPP4 | Dipeptidyl Peptidase 4 | P27487 |
| MATN1 | Matrilin 1 | P21941 |
| LAMB2 | Laminin Subunit Beta 2 | P55268 |
| VPS33A | VPS33A Core Subunit Of CORVET And HOPS Complexes | Q96AX1 |
| DIO2 | Iodothyronine Deiodinase 2 | Q92813 |
| TRIM32 | Tripartite Motif Containing 32 | Q13049 |
| HMBS | Hydroxymethylbilane Synthase | P08397 |
| USP18 | Ubiquitin Specific Peptidase 18 | Q9UMW8 |
| RNY3 | RNA, Ro60-Associated Y3 |  |
| UCP2 | Uncoupling Protein 2 | P55851 |
| FIBP | FGF1 Intracellular Binding Protein | O43427 |
| CCL18 | C-C Motif Chemokine Ligand 18 | P55774 |
| DDR2 | Discoidin Domain Receptor Tyrosine Kinase 2 | Q16832 |
| ETS2 | ETS Proto-Oncogene 2, Transcription Factor | P15036 |
| ITGA5 | Integrin Subunit Alpha 5 | P08648 |
| CLNK | Cytokine Dependent Hematopoietic Cell Linker | Q7Z7G1 |
| CCR3 | C-C Motif Chemokine Receptor 3 | P51677 |
| PHACTR1 | Phosphatase And Actin Regulator 1 | Q9C0D0 |
| THRA | Thyroid Hormone Receptor Alpha | P10827 |
| LRP1 | LDL Receptor Related Protein 1 | Q07954 |
| HOXA9 | Homeobox A9 | P31269 |
| AVP | Arginine Vasopressin | P01185 |
| SFTA3 | Surfactant Associated 3 | P0C7M3 |
| EMD | Emerin | P50402 |
| CTNNA1 | Catenin Alpha 1 | P35221 |
| GTF2E2 | General Transcription Factor IIE Subunit 2 | P29084 |
| DDX41 | DEAD-Box Helicase 41 | Q9UJV9 |
| CGA | Glycoprotein Hormones, Alpha Polypeptide | P01215 |
| XBP1 | X-Box Binding Protein 1 | P17861 |
| MATR3 | Matrin 3 | P43243 |
| EGR2 | Early Growth Response 2 | P11161 |
| DDIT4 | DNA Damage Inducible Transcript 4 | Q9NX09 |
| DLL3 | Delta Like Canonical Notch Ligand 3 | Q9NYJ7 |
| INHBA | Inhibin Subunit Beta A | P08476 |
| MIR874 | MicroRNA 874 |  |
| F2RL2 | Coagulation Factor II Thrombin Receptor Like 2 | O00254 |
| NCOA2 | Nuclear Receptor Coactivator 2 | Q15596 |
| MFGE8 | Milk Fat Globule EGF And Factor V/VIII Domain Containing | Q08431 |
| PEX2 | Peroxisomal Biogenesis Factor 2 | P28328 |
| UTRN | Utrophin | P46939 |
| PAPPA | Pappalysin 1 | Q13219 |
| AMPD1 | Adenosine Monophosphate Deaminase 1 | P23109 |
| NR1H4 | Nuclear Receptor Subfamily 1 Group H Member 4 | Q96RI1 |
| SGCB | Sarcoglycan Beta | Q16585 |
| FRZB | Frizzled Related Protein | Q92765 |
| SPTLC1 | Serine Palmitoyltransferase Long Chain Base Subunit 1 | O15269 |
| FTO | FTO Alpha-Ketoglutarate Dependent Dioxygenase | Q9C0B1 |
| CHST14 | Carbohydrate Sulfotransferase 14 | Q8NCH0 |
| TMEM126B | Transmembrane Protein 126B | Q8IUX1 |
| TMEM237 | Transmembrane Protein 237 | Q96Q45 |
| POLR1A | RNA Polymerase I Subunit A | O95602 |
| HIC1 | HIC ZBTB Transcriptional Repressor 1 | Q14526 |
| USP2 | Ubiquitin Specific Peptidase 2 | O75604 |
| MIR381 | MicroRNA 381 |  |
| SAMD9L | Sterile Alpha Motif Domain Containing 9 Like | Q8IVG5 |
| MFSD8 | Major Facilitator Superfamily Domain Containing 8 | Q8NHS3 |
| SFRP1 | Secreted Frizzled Related Protein 1 | Q8N474 |
| M6PR | Mannose-6-Phosphate Receptor, Cation Dependent | P20645 |
| HTR1A | 5-Hydroxytryptamine Receptor 1A | P08908 |
| PTGER2 | Prostaglandin E Receptor 2 | P43116 |
| TMEM38B | Transmembrane Protein 38B | Q9NVV0 |
| PHF21A | PHD Finger Protein 21A | Q96BD5 |
| TRH | Thyrotropin Releasing Hormone | P20396 |
| TAF6 | TATA-Box Binding Protein Associated Factor 6 | P49848 |
| FDPS | Farnesyl Diphosphate Synthase | P14324 |
| BNIP1 | BCL2 Interacting Protein 1 | Q12981 |
| HCCAT5 | Hepatocellular Carcinoma Associated Transcript 5 |  |
| MIR4306 | MicroRNA 4306 |  |
| PIGY | Phosphatidylinositol Glycan Anchor Biosynthesis Class Y | Q3MUY2 |
| FOXA1 | Forkhead Box A1 | P55317 |
| USP4 | Ubiquitin Specific Peptidase 4 | Q13107 |
| SLCO2A1 | Solute Carrier Organic Anion Transporter Family Member 2A1 | Q92959 |
| AGRN | Agrin | O00468 |
| SLC2A3 | Solute Carrier Family 2 Member 3 | P11169 |
| MYH2 | Myosin Heavy Chain 2 | Q9UKX2 |
| CYP11B1 | Cytochrome P450 Family 11 Subfamily B Member 1 | P15538 |
| SERAC1 | Serine Active Site Containing 1 | Q96JX3 |
| NR3C2 | Nuclear Receptor Subfamily 3 Group C Member 2 | P08235 |
| CTSA | Cathepsin A | P10619 |
| KCNQ3 | Potassium Voltage-Gated Channel Subfamily Q Member 3 | O43525 |
| DUSP19 | Dual Specificity Phosphatase 19 | Q8WTR2 |
| TNXB | Tenascin XB | P22105 |
| NR4A1 | Nuclear Receptor Subfamily 4 Group A Member 1 | P22736 |
| SLC19A1 | Solute Carrier Family 19 Member 1 | P41440 |
| ANK3 | Ankyrin 3 | Q12955 |
| FMOD | Fibromodulin | Q06828 |
| ANXA11 | Annexin A11 | P50995 |
| SERPINF2 | Serpin Family F Member 2 | P08697 |
| SALL1 | Spalt Like Transcription Factor 1 | Q9NSC2 |
| UTP4 | UTP4 Small Subunit Processome Component | Q969X6 |
| CCL7 | C-C Motif Chemokine Ligand 7 | P80098 |
| PINK1 | PTEN Induced Kinase 1 | Q9BXM7 |
| DVL2 | Dishevelled Segment Polarity Protein 2 | O14641 |
| GHSR | Growth Hormone Secretagogue Receptor | Q92847 |
| PEX14 | Peroxisomal Biogenesis Factor 14 | O75381 |
| EXO1 | Exonuclease 1 | Q9UQ84 |
| GAL | Galanin And GMAP Prepropeptide | P22466 |
| COLEC10 | Collectin Subfamily Member 10 | Q9Y6Z7 |
| CXCL11 | C-X-C Motif Chemokine Ligand 11 | O14625 |
| FAM20C | FAM20C Golgi Associated Secretory Pathway Kinase | Q8IXL6 |
| LGR5 | Leucine Rich Repeat Containing G Protein-Coupled Receptor 5 | O75473 |
| MIR196A2 | MicroRNA 196a-2 |  |
| DGCR8 | DGCR8 Microprocessor Complex Subunit | Q8WYQ5 |
| DNAI2 | Dynein Axonemal Intermediate Chain 2 | Q9GZS0 |
| PMP22 | Peripheral Myelin Protein 22 | Q01453 |
| EXOSC9 | Exosome Component 9 | Q06265 |
| CD83 | CD83 Molecule | Q01151 |
| UFL1 | UFM1 Specific Ligase 1 | O94874 |
| AP3B1 | Adaptor Related Protein Complex 3 Subunit Beta 1 | O00203 |
| CACNA1H | Calcium Voltage-Gated Channel Subunit Alpha1 H | O95180 |
| CCBE1 | Collagen And Calcium Binding EGF Domains 1 | Q6UXH8 |
| DNM3 | Dynamin 3 | Q9UQ16 |
| TELO2 | Telomere Maintenance 2 | Q9Y4R8 |
| F9 | Coagulation Factor IX | P00740 |
| FZD5 | Frizzled Class Receptor 5 | Q13467 |
| TRD-GTC9-1 | TRNA-Asp (Anticodon GTC) 9-1 |  |
| NCOA1 | Nuclear Receptor Coactivator 1 | Q15788 |
| SLC25A13 | Solute Carrier Family 25 Member 13 | Q9UJS0 |
| ITGAV | Integrin Subunit Alpha V | P06756 |
| MCM5 | Minichromosome Maintenance Complex Component 5 | P33992 |
| CYP26C1 | Cytochrome P450 Family 26 Subfamily C Member 1 | Q6V0L0 |
| LMBRD1 | LMBR1 Domain Containing 1 | Q9NUN5 |
| RIN2 | Ras And Rab Interactor 2 | Q8WYP3 |
| KISS1R | KISS1 Receptor | Q969F8 |
| HIBCH | 3-Hydroxyisobutyryl-CoA Hydrolase | Q6NVY1 |
| TCF7 | Transcription Factor 7 | P36402 |
| AGK | Acylglycerol Kinase | Q53H12 |
| WDPCP | WD Repeat Containing Planar Cell Polarity Effector | O95876 |
| CUX1 | Cut Like Homeobox 1 | Q13948 |
| HNRNPA2B1 | Heterogeneous Nuclear Ribonucleoprotein A2/B1 | P22626 |
| APOC3 | Apolipoprotein C3 | P02656 |
| NR1H2 | Nuclear Receptor Subfamily 1 Group H Member 2 | P55055 |
| PSMA7 | Proteasome 20S Subunit Alpha 7 | O14818 |
| RNPC3 | RNA Binding Region (RNP1, RRM) Containing 3 | Q96LT9 |
| GALNT3 | Polypeptide N-Acetylgalactosaminyltransferase 3 | Q14435 |
| GYPA | Glycophorin A (MNS Blood Group) | P02724 |
| PDP1 | Pyruvate Dehydrogenase Phosphatase Catalytic Subunit 1 | Q9P0J1 |
| DLEU1 | Deleted In Lymphocytic Leukemia 1 | O43261 |
| SGCD | Sarcoglycan Delta | Q92629 |
| NEB | Nebulin | P20929 |
| MIR3922 | MicroRNA 3922 |  |
| DANCR | Differentiation Antagonizing Non-Protein Coding RNA | P0C864 |
| HMGA1 | High Mobility Group AT-Hook 1 | P17096 |
| GDF2 | Growth Differentiation Factor 2 | Q9UK05 |
| PTPA | Protein Phosphatase 2 Phosphatase Activator | Q15257 |
| KDM6B | Lysine Demethylase 6B | O15054 |
| SMG8 | SMG8 Nonsense Mediated MRNA Decay Factor | Q8ND04 |
| HMMR | Hyaluronan Mediated Motility Receptor | O75330 |
| PIN1 | Peptidylprolyl Cis/Trans Isomerase, NIMA-Interacting 1 | Q13526 |
| KPNB1 | Karyopherin Subunit Beta 1 | Q14974 |
| CRAT | Carnitine O-Acetyltransferase | P43155 |
| ARSH | Arylsulfatase Family Member H | Q5FYA8 |
| EZH1 | Enhancer Of Zeste 1 Polycomb Repressive Complex 2 Subunit | Q92800 |
| TSEN34 | TRNA Splicing Endonuclease Subunit 34 | Q9BSV6 |
| AXIN2 | Axin 2 | Q9Y2T1 |
| RBP4 | Retinol Binding Protein 4 | P02753 |
| ARRB2 | Arrestin Beta 2 | P32121 |
| POU2F1 | POU Class 2 Homeobox 1 | P14859 |
| CSMD3 | CUB And Sushi Multiple Domains 3 | Q7Z407 |
| CLEC4A | C-Type Lectin Domain Family 4 Member A | Q9UMR7 |
| RBL2 | RB Transcriptional Corepressor Like 2 | Q08999 |
| NUDT6 | Nudix Hydrolase 6 | P53370 |
| ADORA3 | Adenosine A3 Receptor | P0DMS8 |
| GPER1 | G Protein-Coupled Estrogen Receptor 1 | Q99527 |
| PNPT1 | Polyribonucleotide Nucleotidyltransferase 1 | Q8TCS8 |
| LOC126862586 | CDK7 Strongly-Dependent Group 2 Enhancer GRCh37_chr17:48273702-48274901 |  |
| PPARGC1B | PPARG Coactivator 1 Beta | Q86YN6 |
| TMEM165 | Transmembrane Protein 165 | Q9HC07 |
| FABP3 | Fatty Acid Binding Protein 3 | P05413 |
| CRABP2 | Cellular Retinoic Acid Binding Protein 2 | P29373 |
| RPS6KA5 | Ribosomal Protein S6 Kinase A5 | O75582 |
| PAK4 | P21 (RAC1) Activated Kinase 4 | O96013 |
| KAT5 | Lysine Acetyltransferase 5 | Q92993 |
| ERCC6L2 | ERCC Excision Repair 6 Like 2 | Q5T890 |
| LIPE | Lipase E, Hormone Sensitive Type | Q05469 |
| NCOR2 | Nuclear Receptor Corepressor 2 | Q9Y618 |
| HNMT | Histamine N-Methyltransferase | P50135 |
| MTUS2 | Microtubule Associated Scaffold Protein 2 | Q5JR59 |
| MYF6 | Myogenic Factor 6 | P23409 |
| SIRT6 | Sirtuin 6 | Q8N6T7 |
| VSX1 | Visual System Homeobox 1 | Q9NZR4 |
| ANO5 | Anoctamin 5 | Q75V66 |
| ACD | ACD Shelterin Complex Subunit And Telomerase Recruitment Factor | Q96AP0 |
| CGB5 | Chorionic Gonadotropin Subunit Beta 5 | P0DN86 |
| UBE2I | Ubiquitin Conjugating Enzyme E2 I | P63279 |
| ID2 | Inhibitor Of DNA Binding 2 | Q02363 |
| HAX1 | HCLS1 Associated Protein X-1 | O00165 |
| APOA4 | Apolipoprotein A4 | P06727 |
| FXN | Frataxin | Q16595 |
| ACADM | Acyl-CoA Dehydrogenase Medium Chain | P11310 |
| ABCC8 | ATP Binding Cassette Subfamily C Member 8 | Q09428 |
| TRPV5 | Transient Receptor Potential Cation Channel Subfamily V Member 5 | Q9NQA5 |
| RREB1 | Ras Responsive Element Binding Protein 1 | Q92766 |
| RNU6ATAC | RNA, U6atac Small Nuclear |  |
| HAMP | Hepcidin Antimicrobial Peptide | P81172 |
| NBAS | NBAS Subunit Of NRZ Tethering Complex | A2RRP1 |
| MYO16 | Myosin XVI | Q9Y6X6 |
| IGFBP6 | Insulin Like Growth Factor Binding Protein 6 | P24592 |
| RPS15A | Ribosomal Protein S15a | P62244 |
| SLC39A7 | Solute Carrier Family 39 Member 7 | Q92504 |
| KMT2C | Lysine Methyltransferase 2C | Q8NEZ4 |
| MIR615 | MicroRNA 615 |  |
| IGFBP4 | Insulin Like Growth Factor Binding Protein 4 | P22692 |
| ALOX15 | Arachidonate 15-Lipoxygenase | P16050 |
| HOXB1 | Homeobox B1 | P14653 |
| TNNT1 | Troponin T1, Slow Skeletal Type | P13805 |
| OCRL | OCRL Inositol Polyphosphate-5-Phosphatase | Q01968 |
| RAB33A | RAB33A, Member RAS Oncogene Family | Q14088 |
| GMDS-DT | GMDS Divergent Transcript |  |
| LBX1-AS1 | LBX1 Antisense RNA 1 |  |
| DGKQ | Diacylglycerol Kinase Theta | P52824 |
| CRPPA | CDP-L-Ribitol Pyrophosphorylase A | A4D126 |
| PCAT1 | Prostate Cancer Associated Transcript 1 |  |
| TRAPPC2B | Trafficking Protein Particle Complex Subunit 2B | P0DI82 |
| ASPH | Aspartate Beta-Hydroxylase | Q12797 |
| CHPF | Chondroitin Polymerizing Factor | Q8IZ52 |
| UFSP1 | UFM1 Specific Peptidase 1 (Inactive) | Q6NVU6 |
| QRICH1 | Glutamine Rich 1 | Q2TAL8 |
| HTR3A | 5-Hydroxytryptamine Receptor 3A | P46098 |
| WNT11 | Wnt Family Member 11 | O96014 |
| BLOC1S1 | Biogenesis Of Lysosomal Organelles Complex 1 Subunit 1 | P78537 |
| CILK1 | Ciliogenesis Associated Kinase 1 | Q9UPZ9 |
| FKBP1A | FKBP Prolyl Isomerase 1A | P62942 |
| DYNC2I2 | Dynein 2 Intermediate Chain 2 | Q96EX3 |
| PRKCQ | Protein Kinase C Theta | Q04759 |
| FABP2 | Fatty Acid Binding Protein 2 | P12104 |
| TET3 | Tet Methylcytosine Dioxygenase 3 | O43151 |
| GP1BB | Glycoprotein Ib Platelet Subunit Beta | P13224 |
| RNU6-1 | RNA, U6 Small Nuclear 1 |  |
| DDHD1 | DDHD Domain Containing 1 | Q8NEL9 |
| LPA | Lipoprotein(A) | P08519 |
| MIR155HG | MIR155 Host Gene |  |
| CCN4 | Cellular Communication Network Factor 4 | O95388 |
| RNU6-2 | RNA, U6 Small Nuclear 2 |  |
| RNU6-7 | RNA, U6 Small Nuclear 7 |  |
| RNU6-8 | RNA, U6 Small Nuclear 8 |  |
| RNU6-9 | RNA, U6 Small Nuclear 9 |  |
| RNU6-1-001 | |  |
| RNU6-1-002 | |  |
| RNU6-1-003 | |  |
| RNU6-1-004 | |  |
| RAB18 | RAB18, Member RAS Oncogene Family | Q9NP72 |
| CLCN3 | Chloride Voltage-Gated Channel 3 | P51790 |
| MIR1207 | MicroRNA 1207 |  |
| SLC26A1 | Solute Carrier Family 26 Member 1 | Q9H2B4 |
| LINC01191 | Long Intergenic Non-Protein Coding RNA 1191 |  |
| CYP2R1 | Cytochrome P450 Family 2 Subfamily R Member 1 | Q6VVX0 |
| HYDIN | HYDIN Axonemal Central Pair Apparatus Protein | Q4G0P3 |
| KIF5C | Kinesin Family Member 5C | O60282 |
| CLDN5 | Claudin 5 | O00501 |
| MAD2L1 | Mitotic Arrest Deficient 2 Like 1 | Q13257 |
| P2RY12 | Purinergic Receptor P2Y12 | Q9H244 |
| SPG7 | SPG7 Matrix AAA Peptidase Subunit, Paraplegin | Q9UQ90 |
| MDK | Midkine | P21741 |
| RORA | RAR Related Orphan Receptor A | P35398 |
| LUC7L2 | LUC7 Like 2, Pre-MRNA Splicing Factor | Q9Y383 |
| LEF1 | Lymphoid Enhancer Binding Factor 1 | Q9UJU2 |
| MIR26A1 | MicroRNA 26a-1 |  |
| RPL38 | Ribosomal Protein L38 | P63173 |
| ARG1 | Arginase 1 | P05089 |
| CD2AP | CD2 Associated Protein | Q9Y5K6 |
| ASH1L | ASH1 Like Histone Lysine Methyltransferase | Q9NR48 |
| SNRNP200 | Small Nuclear Ribonucleoprotein U5 Subunit 200 | O75643 |
| SNHG12 | Small Nucleolar RNA Host Gene 12 | Q9BXW3 |
| PAPPA2 | Pappalysin 2 | Q9BXP8 |
| MAN2C1 | Mannosidase Alpha Class 2C Member 1 | Q9NTJ4 |
| CYP27A1 | Cytochrome P450 Family 27 Subfamily A Member 1 | Q02318 |
| ALCAM | Activated Leukocyte Cell Adhesion Molecule | Q13740 |
| ADAMTSL4 | ADAMTS Like 4 | Q6UY14 |
| XPO1 | Exportin 1 | O14980 |
| ASAH2 | N-Acylsphingosine Amidohydrolase 2 | Q9NR71 |
| EXOSC5 | Exosome Component 5 | Q9NQT4 |
| MEF2A | Myocyte Enhancer Factor 2A | Q02078 |
| ENSG00000232995 | Regulator Of G Protein Signaling 5 |  |
| PPP1R12A | Protein Phosphatase 1 Regulatory Subunit 12A | O14974 |
| FAM201A | Family With Sequence Similarity 201 Member A | Q5SY85 |
| GRB10 | Growth Factor Receptor Bound Protein 10 | Q13322 |
| SIRT3 | Sirtuin 3 | Q9NTG7 |
| S100A2 | S100 Calcium Binding Protein A2 | P29034 |
| ST3GAL4 | ST3 Beta-Galactoside Alpha-2,3-Sialyltransferase 4 | Q11206 |
| RPL22 | Ribosomal Protein L22 | P35268 |
| IRF9 | Interferon Regulatory Factor 9 | Q00978 |
| SPAST | Spastin | Q9UBP0 |
| G6PC3 | Glucose-6-Phosphatase Catalytic Subunit 3 | Q9BUM1 |
| MIR340 | MicroRNA 340 |  |
| ADIPOR1 | Adiponectin Receptor 1 | Q96A54 |
| COQ9 | Coenzyme Q9 | O75208 |
| ATP13A2 | ATPase Cation Transporting 13A2 | Q9NQ11 |
| MIR645 | MicroRNA 645 |  |
| PISD | Phosphatidylserine Decarboxylase | Q9UG56 |
| EXOSC8 | Exosome Component 8 | Q96B26 |
| RAB43 | RAB43, Member RAS Oncogene Family | Q86YS6 |
| IL21R | Interleukin 21 Receptor | Q9HBE5 |
| TMEM70 | Transmembrane Protein 70 | Q9BUB7 |
| TET2 | Tet Methylcytosine Dioxygenase 2 | Q6N021 |
| STMN2 | Stathmin 2 | Q93045 |
| CNTLN | Centlein | Q9NXG0 |
| HABP2 | Hyaluronan Binding Protein 2 | Q14520 |
| CTNS | Cystinosin, Lysosomal Cystine Transporter | O60931 |
| KLF2 | KLF Transcription Factor 2 | Q9Y5W3 |
| GFPT1 | Glutamine--Fructose-6-Phosphate Transaminase 1 | Q06210 |
| HBA1 | Hemoglobin Subunit Alpha 1 | P69905 |
| MIRLET7A3 | MicroRNA Let-7a-3 |  |
| RECQL | RecQ Like Helicase | P46063 |
| MKKS | MKKS Centrosomal Shuttling Protein | Q9NPJ1 |
| PDCD4 | Programmed Cell Death 4 | Q53EL6 |
| MIRLET7A2 | MicroRNA Let-7a-2 |  |
| CDKAL1 | CDK5 Regulatory Subunit Associated Protein 1 Like 1 | Q5VV42 |
| HRH2 | Histamine Receptor H2 | P25021 |
| HBG2 | Hemoglobin Subunit Gamma 2 | P69892 |
| PIGB | Phosphatidylinositol Glycan Anchor Biosynthesis Class B | Q92521 |
| TTPA | Alpha Tocopherol Transfer Protein | P49638 |
| FST | Follistatin | P19883 |
| IGF2-AS | IGF2 Antisense RNA | Q6U949 |
| PSPH | Phosphoserine Phosphatase | P78330 |
| UFD1 | Ubiquitin Recognition Factor In ER Associated Degradation 1 | Q92890 |
| MYOCD | Myocardin | Q8IZQ8 |
| IFNAR2 | Interferon Alpha And Beta Receptor Subunit 2 | P48551 |
| BMP3 | Bone Morphogenetic Protein 3 | P12645 |
| GFI1 | Growth Factor Independent 1 Transcriptional Repressor | Q99684 |
| ABCB11 | ATP Binding Cassette Subfamily B Member 11 | O95342 |
| MAP2 | Microtubule Associated Protein 2 | P11137 |
| ATP2A2 | ATPase Sarcoplasmic/Endoplasmic Reticulum Ca2+ Transporting 2 | P16615 |
| AOC3 | Amine Oxidase Copper Containing 3 | Q16853 |
| MIR585 | MicroRNA 585 |  |
| KCNJ11 | Potassium Inwardly Rectifying Channel Subfamily J Member 11 | Q14654 |
| MIR582 | MicroRNA 582 |  |
| SLC30A2 | Solute Carrier Family 30 Member 2 | Q9BRI3 |
| LARGE1 | LARGE Xylosyl- And Glucuronyltransferase 1 | O95461 |
| DSE | Dermatan Sulfate Epimerase | Q9UL01 |
| PAX5 | Paired Box 5 | Q02548 |
| ADM2 | Adrenomedullin 2 | Q7Z4H4 |
| CD38 | CD38 Molecule | P28907 |
| CANX | Calnexin | P27824 |
| RBL1 | RB Transcriptional Corepressor Like 1 | P28749 |
| SPTB | Spectrin Beta, Erythrocytic | P11277 |
| EXOSC1 | Exosome Component 1 | Q9Y3B2 |
| FBF1 | Fas Binding Factor 1 | Q8TES7 |
| OFCC1 | Orofacial Cleft 1 Candidate 1 (Pseudogene) | Q8IZS5 |
| TRE-TTC3-1 | TRNA-Glu (Anticodon TTC) 3-1 |  |
| ANXA6 | Annexin A6 | P08133 |
| MIR138-1 | MicroRNA 138-1 |  |
| PIGT | Phosphatidylinositol Glycan Anchor Biosynthesis Class T | Q969N2 |
| ATG7 | Autophagy Related 7 | O95352 |
| TAGLN | Transgelin | Q01995 |
| FSHR | Follicle Stimulating Hormone Receptor | P23945 |
| LIPA | Lipase A, Lysosomal Acid Type | P38571 |
| GANAB | Glucosidase II Alpha Subunit | Q14697 |
| NR5A2 | Nuclear Receptor Subfamily 5 Group A Member 2 | O00482 |
| PTN | Pleiotrophin | P21246 |
| IFT81 | Intraflagellar Transport 81 | Q8WYA0 |
| ACVR2A | Activin A Receptor Type 2A | P27037 |
| MC2R | Melanocortin 2 Receptor | Q01718 |
| TUBA4A | Tubulin Alpha 4a | P68366 |
| GJA4 | Gap Junction Protein Alpha 4 | P35212 |
| MIR4709 | MicroRNA 4709 |  |
| MIR4747 | MicroRNA 4747 |  |
| MIR5572 | MicroRNA 5572 |  |
| MIR4441 | MicroRNA 4441 |  |
| MIR4472-1 | MicroRNA 4472-1 |  |
| MIR4472-2 | MicroRNA 4472-2 |  |
| DGCR5 | DiGeorge Syndrome Critical Region Gene 5 |  |
| AVPR2 | Arginine Vasopressin Receptor 2 | P30518 |
| OPN1LW | Opsin 1, Long Wave Sensitive | P04000 |
| IFIT1 | Interferon Induced Protein With Tetratricopeptide Repeats 1 | P09914 |
| KLF10 | KLF Transcription Factor 10 | Q13118 |
| HGD | Homogentisate 1,2-Dioxygenase | Q93099 |
| TGFBR3 | Transforming Growth Factor Beta Receptor 3 | Q03167 |
| NPHS1 | NPHS1 Adhesion Molecule, Nephrin | O60500 |
| CGB3 | Chorionic Gonadotropin Subunit Beta 3 | P0DN86 |
| TRPA1 | Transient Receptor Potential Cation Channel Subfamily A Member 1 | O75762 |
| PRR12 | Proline Rich 12 | Q9ULL5 |
| STS | Steroid Sulfatase | P08842 |
| SCT | Secretin | P09683 |
| SMAD9 | SMAD Family Member 9 | O15198 |
| TBC1D20 | TBC1 Domain Family Member 20 | Q96BZ9 |
| CARD9 | Caspase Recruitment Domain Family Member 9 | Q9H257 |
| FGD5-AS1 | FGD5 Antisense RNA 1 |  |
| CLN3 | CLN3 Lysosomal/Endosomal Transmembrane Protein, Battenin | Q13286 |
| SH2B3 | SH2B Adaptor Protein 3 | Q9UQQ2 |
| NFATC2 | Nuclear Factor Of Activated T Cells 2 | Q13469 |
| KLK3 | Kallikrein Related Peptidase 3 | P07288 |
| SEPTIN9 | Septin 9 | Q9UHD8 |
| LOC107133510 | Origin Of Replication At HBB |  |
| SHOX2 | SHOX Homeobox 2 | O60902 |
| XPR1 | Xenotropic And Polytropic Retrovirus Receptor 1 | Q9UBH6 |
| RNF213 | Ring Finger Protein 213 | Q63HN8 |
| ACVR2B | Activin A Receptor Type 2B | Q13705 |
| RAP1A | RAP1A, Member Of RAS Oncogene Family | P62834 |
| JAK3 | Janus Kinase 3 | P52333 |
| EWSAT1 | Ewing Sarcoma Associated Transcript 1 |  |
| SLC1A5 | Solute Carrier Family 1 Member 5 | Q15758 |
| ARRB1 | Arrestin Beta 1 | P49407 |
| POLI | DNA Polymerase Iota | Q9UNA4 |
| SCGB1A1 | Secretoglobin Family 1A Member 1 | P11684 |
| AASS | Aminoadipate-Semialdehyde Synthase | Q9UDR5 |
| CLN6 | CLN6 Transmembrane ER Protein | Q9NWW5 |
| BMP15 | Bone Morphogenetic Protein 15 | O95972 |
| THRB | Thyroid Hormone Receptor Beta | P10828 |
| DYNLT2B | Dynein Light Chain Tctex-Type 2B | Q8WW35 |
| EMILIN1 | Elastin Microfibril Interfacer 1 | Q9Y6C2 |
| PCK2 | Phosphoenolpyruvate Carboxykinase 2, Mitochondrial | Q16822 |
| HES1 | Hes Family BHLH Transcription Factor 1 | Q14469 |
| MIR1246 | MicroRNA 1246 |  |
| ADORA1 | Adenosine A1 Receptor | P30542 |
| VPS35 | VPS35 Retromer Complex Component | Q96QK1 |
| FRS2 | Fibroblast Growth Factor Receptor Substrate 2 | Q8WU20 |
| ABCG1 | ATP Binding Cassette Subfamily G Member 1 | P45844 |
| TFEB | Transcription Factor EB | P19484 |
| CHRM2 | Cholinergic Receptor Muscarinic 2 | P08172 |
| RPS15 | Ribosomal Protein S15 | P62841 |
| CETP | Cholesteryl Ester Transfer Protein | P11597 |
| RPL6 | Ribosomal Protein L6 | Q02878 |
| ANK1 | Ankyrin 1 | P16157 |
| SRP72 | Signal Recognition Particle 72 | O76094 |
| PRPF31 | Pre-MRNA Processing Factor 31 | Q8WWY3 |
| ATF6 | Activating Transcription Factor 6 | P18850 |
| GRK2 | G Protein-Coupled Receptor Kinase 2 | P25098 |
| MYT1L | Myelin Transcription Factor 1 Like | Q9UL68 |
| DNAJB11 | DnaJ Heat Shock Protein Family (Hsp40) Member B11 | Q9UBS4 |
| STX6 | Syntaxin 6 | O43752 |
| PANDAR | Promoter Of CDKN1A Antisense DNA Damage Activated RNA |  |
| NPR3 | Natriuretic Peptide Receptor 3 | P17342 |
| NEUROD1 | Neuronal Differentiation 1 | Q13562 |
| SCYL2 | SCY1 Like Pseudokinase 2 | Q6P3W7 |
| HMGB2 | High Mobility Group Box 2 | P26583 |
| APOA5 | Apolipoprotein A5 | Q6Q788 |
| SHROOM3 | Shroom Family Member 3 | Q8TF72 |
| FZD7 | Frizzled Class Receptor 7 | O75084 |
| SI | Sucrase-Isomaltase | P14410 |
| MAP3K20 | Mitogen-Activated Protein Kinase Kinase Kinase 20 | Q9NYL2 |
| PTGIR | Prostaglandin I2 Receptor | P43119 |
| TBX20 | T-Box Transcription Factor 20 | Q9UMR3 |
| ALMS1 | ALMS1 Centrosome And Basal Body Associated Protein | Q8TCU4 |
| NUTM1 | NUT Midline Carcinoma Family Member 1 | Q86Y26 |
| PRPF4 | Pre-MRNA Processing Factor 4 | O43172 |
| SLC30A1 | Solute Carrier Family 30 Member 1 | Q9Y6M5 |
| CDK9 | Cyclin Dependent Kinase 9 | P50750 |
| GAA | Alpha Glucosidase | P10253 |
| H4C16 | H4 Histone 16 | P62805 |
| CS | Citrate Synthase | O75390 |
| P2RX5-TAX1BP3 | P2RX5-TAX1BP3 Readthrough (NMD Candidate) |  |
| CCDC85B | Coiled-Coil Domain Containing 85B | Q15834 |
| UAP1 | UDP-N-Acetylglucosamine Pyrophosphorylase 1 | Q16222 |
| TERF1 | Telomeric Repeat Binding Factor 1 | P54274 |
| RAB5A | RAB5A, Member RAS Oncogene Family | P20339 |
| CACNA1D | Calcium Voltage-Gated Channel Subunit Alpha1 D | Q01668 |
| HYCC1 | Hyccin PI4KA Lipid Kinase Complex Subunit 1 | Q9BYI3 |
| PTGDR | Prostaglandin D2 Receptor | Q13258 |
| ADAM15 | ADAM Metallopeptidase Domain 15 | Q13444 |
| RAB27A | RAB27A, Member RAS Oncogene Family | P51159 |
| CTNNBIP1 | Catenin Beta Interacting Protein 1 | Q9NSA3 |
| SLC39A6 | Solute Carrier Family 39 Member 6 | Q13433 |
| CLCF1 | Cardiotrophin Like Cytokine Factor 1 | Q9UBD9 |
| HRH1 | Histamine Receptor H1 | P35367 |
| MANF | Mesencephalic Astrocyte Derived Neurotrophic Factor | P55145 |
| TERF2 | Telomeric Repeat Binding Factor 2 | Q15554 |
| TK1 | Thymidine Kinase 1 | P04183 |
| PYCR1 | Pyrroline-5-Carboxylate Reductase 1 | P32322 |
| ANKRD26 | Ankyrin Repeat Domain Containing 26 | Q9UPS8 |
| AP1S1 | Adaptor Related Protein Complex 1 Subunit Sigma 1 | P61966 |
| PACSIN3 | Protein Kinase C And Casein Kinase Substrate In Neurons 3 | Q9UKS6 |
| ELOF1 | Elongation Factor 1 | P60002 |
| HIRA | Histone Cell Cycle Regulator | P54198 |
| PRKACB | Protein Kinase CAMP-Activated Catalytic Subunit Beta | P22694 |
| ITPA | Inosine Triphosphatase | Q9BY32 |
| CHST2 | Carbohydrate Sulfotransferase 2 | Q9Y4C5 |
| PIGK | Phosphatidylinositol Glycan Anchor Biosynthesis Class K | Q92643 |
| MANBA | Mannosidase Beta | O00462 |
| SLX1A-SULT1A3 | SLX1A-SULT1A3 Readthrough (NMD Candidate) |  |
| CCDC134 | Coiled-Coil Domain Containing 134 | Q9H6E4 |
| GINS1 | GINS Complex Subunit 1 | Q14691 |
| CAV3 | Caveolin 3 | P56539 |
| MIR7-1 | MicroRNA 7-1 |  |
| MIR128-1 | MicroRNA 128-1 |  |
| EPIC1 | Epigenetically Induced MYC Interacting LncRNA 1 |  |
| H4C5 | H4 Clustered Histone 5 | P62805 |
| DROSHA | Drosha Ribonuclease III | Q9NRR4 |
| B4GAT1 | Beta-1,4-Glucuronyltransferase 1 | O43505 |
| PON2 | Paraoxonase 2 | Q15165 |
| CLCN4 | Chloride Voltage-Gated Channel 4 | P51793 |
| THBS3 | Thrombospondin 3 | P49746 |
| TRPC6 | Transient Receptor Potential Cation Channel Subfamily C Member 6 | Q9Y210 |
| VLDLR | Very Low Density Lipoprotein Receptor | P98155 |
| TRPC1 | Transient Receptor Potential Cation Channel Subfamily C Member 1 | P48995 |
| MYO9A | Myosin IXA | B2RTY4 |
| CRIPTO | Cripto, EGF-CFC Family Member | P13385 |
| DHH | Desert Hedgehog Signaling Molecule | O43323 |
| KLF6 | KLF Transcription Factor 6 | Q99612 |
| CRELD2 | Cysteine Rich With EGF Like Domains 2 | Q6UXH1 |
| PIEZO2 | Piezo Type Mechanosensitive Ion Channel Component 2 | Q9H5I5 |
| NR2F2 | Nuclear Receptor Subfamily 2 Group F Member 2 | P24468 |
| RXRB | Retinoid X Receptor Beta | P28702 |
| DGCR2 | DiGeorge Syndrome Critical Region Gene 2 | P98153 |
| RGS5 | Regulator Of G Protein Signaling 5 | O15539 |
| MCM2 | Minichromosome Maintenance Complex Component 2 | P49736 |
| PDLIM5 | PDZ And LIM Domain 5 | Q96HC4 |
| PAEP | Progestagen Associated Endometrial Protein | P09466 |
| DLEU7-AS1 | DLEU7 Antisense RNA 1 |  |
| ADAM8 | ADAM Metallopeptidase Domain 8 | P78325 |
| CGAS | Cyclic GMP-AMP Synthase | Q8N884 |
| ANOS1 | Anosmin 1 | P23352 |
| LIPG | Lipase G, Endothelial Type | Q9Y5X9 |
| SLC29A3 | Solute Carrier Family 29 Member 3 | Q9BZD2 |
| BTD | Biotinidase | P43251 |
| APOC1 | Apolipoprotein C1 | P02654 |
| CSTA | Cystatin A | P01040 |
| LINC00473 | Long Intergenic Non-Protein Coding RNA 473 | A8K010 |
| MIR452 | MicroRNA 452 |  |
| CPQ | Carboxypeptidase Q | Q9Y646 |
| MEAK7 | MTOR Associated Protein, Eak-7 Homolog | Q6P9B6 |
| MIR933 | MicroRNA 933 |  |
| HTR2C | 5-Hydroxytryptamine Receptor 2C | P28335 |
| OLIG2 | Oligodendrocyte Transcription Factor 2 | Q13516 |
| ARNT | Aryl Hydrocarbon Receptor Nuclear Translocator | P27540 |
| CALCRL | Calcitonin Receptor Like Receptor | Q16602 |
| ITLN1 | Intelectin 1 | Q8WWA0 |
| CLCNKB | Chloride Voltage-Gated Channel Kb | P51801 |
| GCKR | Glucokinase Regulator | Q14397 |
| HSP90AB1 | Heat Shock Protein 90 Alpha Family Class B Member 1 | P08238 |
| FLT3 | Fms Related Receptor Tyrosine Kinase 3 | P36888 |
| DUSP6 | Dual Specificity Phosphatase 6 | Q16828 |
| IL34 | Interleukin 34 | Q6ZMJ4 |
| WNT9A | Wnt Family Member 9A | O14904 |
| PYGM | Glycogen Phosphorylase, Muscle Associated | P11217 |
| CRB2 | Crumbs Cell Polarity Complex Component 2 | Q5IJ48 |
| MIR1-1 | MicroRNA 1-1 |  |
| ADIPOR2 | Adiponectin Receptor 2 | Q86V24 |
| MAP3K11 | Mitogen-Activated Protein Kinase Kinase Kinase 11 | Q16584 |
| PER1 | Period Circadian Regulator 1 | O15534 |
| SOX2-OT | SOX2 Overlapping Transcript |  |
| DNASE2 | Deoxyribonuclease 2, Lysosomal | O00115 |
| PAPLN | Papilin, Proteoglycan Like Sulfated Glycoprotein | O95428 |
| ADAMTSL3 | ADAMTS Like 3 | P82987 |
| CA10 | Carbonic Anhydrase 10 | Q9NS85 |
| IDE | Insulin Degrading Enzyme | P14735 |
| TRPM6 | Transient Receptor Potential Cation Channel Subfamily M Member 6 | Q9BX84 |
| SLC22A18 | Solute Carrier Family 22 Member 18 | Q96BI1 |
| MIR9-2 | MicroRNA 9-2 |  |
| SNRPE | Small Nuclear Ribonucleoprotein Polypeptide E | P62304 |
| PRKACG | Protein Kinase CAMP-Activated Catalytic Subunit Gamma | P22612 |
| TRN-GTT2-5 | TRNA-Asn (Anticodon GTT) 2-5 |  |
| TRN-GTT2-6 | TRNA-Asn (Anticodon GTT) 2-6 |  |
| TRN-GTT2-1 | TRNA-Asn (Anticodon GTT) 2-1 |  |
| TRN-GTT2-3 | TRNA-Asn (Anticodon GTT) 2-3 |  |
| TRN-GTT2-2 | TRNA-Asn (Anticodon GTT) 2-2 |  |
| TRN-GTT2-4 | TRNA-Asn (Anticodon GTT) 2-4 |  |
| TRN-GTT2-7 | TRNA-Asn (Anticodon GTT) 2-7 |  |
| TRN-GTT2-8 | TRNA-Asn (Anticodon GTT) 2-8 |  |
| KMT5B | Lysine Methyltransferase 5B | Q4FZB7 |
| EEF2 | Eukaryotic Translation Elongation Factor 2 | P13639 |
| P4HA3 | Prolyl 4-Hydroxylase Subunit Alpha 3 | Q7Z4N8 |
| MSTO1 | Misato Mitochondrial Distribution And Morphology Regulator 1 | Q9BUK6 |
| BSND | Barttin CLCNK Type Accessory Subunit Beta | Q8WZ55 |
| SLC39A10 | Solute Carrier Family 39 Member 10 | Q9ULF5 |
| ALG8 | ALG8 Alpha-1,3-Glucosyltransferase | Q9BVK2 |
| PBX1 | PBX Homeobox 1 | P40424 |
| ANGPTL4 | Angiopoietin Like 4 | Q9BY76 |
| ERC1 | ELKS/RAB6-Interacting/CAST Family Member 1 | Q8IUD2 |
| ZMYND11 | Zinc Finger MYND-Type Containing 11 | Q15326 |
| WNT16 | Wnt Family Member 16 | Q9UBV4 |
| MT-TL1 | Mitochondrially Encoded TRNA-Leu (UUA/G) 1 |  |
| RPS3 | Ribosomal Protein S3 | P23396 |
| POP4 | POP4 Homolog, Ribonuclease P/MRP Subunit | O95707 |
| MTHFS | Methenyltetrahydrofolate Synthetase | P49914 |
| ARHGAP1 | Rho GTPase Activating Protein 1 | Q07960 |
| SLC5A7 | Solute Carrier Family 5 Member 7 | Q9GZV3 |
| RGS2 | Regulator Of G Protein Signaling 2 | P41220 |
| DCD | Dermcidin | P81605 |
| TLX1NB | TLX1 Neighbor | P0CAT3 |
| DNAJB6 | DnaJ Heat Shock Protein Family (Hsp40) Member B6 | O75190 |
| NMNAT1 | Nicotinamide Nucleotide Adenylyltransferase 1 | Q9HAN9 |
| DDHD2 | DDHD Domain Containing 2 | O94830 |
| RUNX1-IT1 | RUNX1 Intronic Transcript 1 |  |
| RIMBP3 | RIMS Binding Protein 3 | Q9UFD9 |
| RYR2 | Ryanodine Receptor 2 | Q92736 |
| ASCC1 | Activating Signal Cointegrator 1 Complex Subunit 1 | Q8N9N2 |
| ADAMTS8 | ADAM Metallopeptidase With Thrombospondin Type 1 Motif 8 | Q9UP79 |
| SLC9A3 | Solute Carrier Family 9 Member A3 | P48764 |
| PPP3R1 | Protein Phosphatase 3 Regulatory Subunit B, Alpha | P63098 |
| CSF2RB | Colony Stimulating Factor 2 Receptor Subunit Beta | P32927 |
| EXTL2 | Exostosin Like Glycosyltransferase 2 | Q9UBQ6 |
| CAMKMT | Calmodulin-Lysine N-Methyltransferase | Q7Z624 |
| PLA2G10 | Phospholipase A2 Group X | O15496 |
| HACE1 | HECT Domain And Ankyrin Repeat Containing E3 Ubiquitin Protein Ligase 1 | Q8IYU2 |
| LRG1 | Leucine Rich Alpha-2-Glycoprotein 1 | P02750 |
| YEATS2 | YEATS Domain Containing 2 | Q9ULM3 |
| TPR | Translocated Promoter Region, Nuclear Basket Protein | P12270 |
| SRGAP2 | SLIT-ROBO Rho GTPase Activating Protein 2 | O75044 |
| PSEN2 | Presenilin 2 | P49810 |
| MTMR2 | Myotubularin Related Protein 2 | Q13614 |
| H4C3 | H4 Clustered Histone 3 | P62805 |
| ANAPC1 | Anaphase Promoting Complex Subunit 1 | Q9H1A4 |
| RGS14 | Regulator Of G Protein Signaling 14 | O43566 |
| TIMM50 | Translocase Of Inner Mitochondrial Membrane 50 | Q3ZCQ8 |
| CDH13 | Cadherin 13 | P55290 |
| SULT1A3 | Sulfotransferase Family 1A Member 3 | P0DMM9 |
| CPA6 | Carboxypeptidase A6 | Q8N4T0 |
| BDKRB1 | Bradykinin Receptor B1 | P46663 |
| CHST11 | Carbohydrate Sulfotransferase 11 | Q9NPF2 |
| GNE | Glucosamine (UDP-N-Acetyl)-2-Epimerase/N-Acetylmannosamine Kinase | Q9Y223 |
| EDN3 | Endothelin 3 | P14138 |
| OMD | Osteomodulin | Q99983 |
| DMBT1 | Deleted In Malignant Brain Tumors 1 | Q9UGM3 |
| FNDC5 | Fibronectin Type III Domain Containing 5 | Q8NAU1 |
| MC4R | Melanocortin 4 Receptor | P32245 |
| MCM3 | Minichromosome Maintenance Complex Component 3 | P25205 |
| LINC00520 | Long Intergenic Non-Protein Coding RNA 520 |  |
| CARMN | Cardiac Mesoderm Enhancer-Associated Non-Coding RNA |  |
| ZMYND10 | Zinc Finger MYND-Type Containing 10 | O75800 |
| PLCB3 | Phospholipase C Beta 3 | Q01970 |
| MED15 | Mediator Complex Subunit 15 | Q96RN5 |
| CEP19 | Centrosomal Protein 19 | Q96LK0 |
| METTL14 | Methyltransferase 14, N6-Adenosine-Methyltransferase Subunit | Q9HCE5 |
| PLIN1 | Perilipin 1 | O60240 |
| PTGIS | Prostaglandin I2 Synthase | Q16647 |
| CYP51A1 | Cytochrome P450 Family 51 Subfamily A Member 1 | Q16850 |
| COL12A1 | Collagen Type XII Alpha 1 Chain | Q99715 |
| PREPL | Prolyl Endopeptidase Like | Q4J6C6 |
| MIR137HG | MIR137 Host Gene |  |
| PKHD1 | PKHD1 Ciliary IPT Domain Containing Fibrocystin/Polyductin | P08F94 |
| ELAVL3 | ELAV Like RNA Binding Protein 3 | Q14576 |
| TRPM8 | Transient Receptor Potential Cation Channel Subfamily M Member 8 | Q7Z2W7 |
| EYA4 | EYA Transcriptional Coactivator And Phosphatase 4 | O95677 |
| CORIN | Corin, Serine Peptidase | Q9Y5Q5 |
| CAST | Calpastatin | P20810 |
| TMEM43 | Transmembrane Protein 43 | Q9BTV4 |
| KCNK3 | Potassium Two Pore Domain Channel Subfamily K Member 3 | O14649 |
| ABCB7 | ATP Binding Cassette Subfamily B Member 7 | O75027 |
| KCNJ1 | Potassium Inwardly Rectifying Channel Subfamily J Member 1 | P48048 |
| ATOX1 | Antioxidant 1 Copper Chaperone | O00244 |
| PHETA1 | PH Domain Containing Endocytic Trafficking Adaptor 1 | Q8N4B1 |
| CHST6 | Carbohydrate Sulfotransferase 6 | Q9GZX3 |
| SLC30A4 | Solute Carrier Family 30 Member 4 | O14863 |
| HSD3B1 | Hydroxy-Delta-5-Steroid Dehydrogenase, 3 Beta- And Steroid Delta-Isomerase 1 | P14060 |
| RPL3 | Ribosomal Protein L3 | P39023 |
| DGAT1 | Diacylglycerol O-Acyltransferase 1 | O75907 |
| ACKR3 | Atypical Chemokine Receptor 3 | P25106 |
| NFKBIZ | NFKB Inhibitor Zeta | Q9BYH8 |
| SLC18A3 | Solute Carrier Family 18 Member A3 | Q16572 |
| SFRP2 | Secreted Frizzled Related Protein 2 | Q96HF1 |
| SERPINA7 | Serpin Family A Member 7 | P05543 |
| KDM3B | Lysine Demethylase 3B | Q7LBC6 |
| SLC4A2 | Solute Carrier Family 4 Member 2 | P04920 |
| SACS | Sacsin Molecular Chaperone | Q9NZJ4 |
| BANCR | BRAF-Activated Non-Protein Coding RNA |  |
| NTN1 | Netrin 1 | O95631 |
| RPL29 | Ribosomal Protein L29 | P47914 |
| HOXD3 | Homeobox D3 | P31249 |
| CNN1 | Calponin 1 | P51911 |
| LCOR | Ligand Dependent Nuclear Receptor Corepressor | Q96JN0 |
| MIR647 | MicroRNA 647 |  |
| NKX3-1 | NK3 Homeobox 1 | Q99801 |
| PLEKHG5 | Pleckstrin Homology And RhoGEF Domain Containing G5 | O94827 |
| SYNE3 | Spectrin Repeat Containing Nuclear Envelope Family Member 3 | Q6ZMZ3 |
| SAR1A | Secretion Associated Ras Related GTPase 1A | Q9NR31 |
| FBXO7 | F-Box Protein 7 | Q9Y3I1 |
| CYP7A1 | Cytochrome P450 Family 7 Subfamily A Member 1 | P22680 |
| MIR654 | MicroRNA 654 |  |
| RP9 | RP9 Pre-MRNA Splicing Factor | Q8TA86 |
| GPKOW | G-Patch Domain And KOW Motifs | Q92917 |
| CERS6 | Ceramide Synthase 6 | Q6ZMG9 |
| EXOSC2 | Exosome Component 2 | Q13868 |
| DINOL | Damage Induced Long Noncoding RNA |  |
| CCNF | Cyclin F | P41002 |
| LTA4H | Leukotriene A4 Hydrolase | P09960 |
| DTNBP1 | Dystrobrevin Binding Protein 1 | Q96EV8 |
| ADAMTS15 | ADAM Metallopeptidase With Thrombospondin Type 1 Motif 15 | Q8TE58 |
| ADAMTS9 | ADAM Metallopeptidase With Thrombospondin Type 1 Motif 9 | Q9P2N4 |
| HSD3B2 | Hydroxy-Delta-5-Steroid Dehydrogenase, 3 Beta- And Steroid Delta-Isomerase 2 | P26439 |
| MIR505 | MicroRNA 505 |  |
| HNF1B | HNF1 Homeobox B | P35680 |
| DPM3 | Dolichyl-Phosphate Mannosyltransferase Subunit 3, Regulatory | Q9P2X0 |
| RNA18SN1 | RNA, 18S Ribosomal N1 |  |
| HYOU1 | Hypoxia Up-Regulated 1 | Q9Y4L1 |
| FDX2 | Ferredoxin 2 | Q6P4F2 |
| IMMP2L | Inner Mitochondrial Membrane Peptidase Subunit 2 | Q96T52 |
| FBXO4 | F-Box Protein 4 | Q9UKT5 |
| SAMD12 | Sterile Alpha Motif Domain Containing 12 | Q8N8I0 |
| SNORD43 | Small Nucleolar RNA, C/D Box 43 |  |
| LUM | Lumican | P51884 |
| GOLIM4 | Golgi Integral Membrane Protein 4 | O00461 |
| CES1 | Carboxylesterase 1 | P23141 |
| SOD3 | Superoxide Dismutase 3 | P08294 |
| NR1I3 | Nuclear Receptor Subfamily 1 Group I Member 3 | Q14994 |
| TACR3 | Tachykinin Receptor 3 | P29371 |
| LHCGR | Luteinizing Hormone/Choriogonadotropin Receptor | P22888 |
| SRP19 | Signal Recognition Particle 19 | P09132 |
| MORC2 | MORC Family CW-Type Zinc Finger 2 | Q9Y6X9 |
| OPCML | Opioid Binding Protein/Cell Adhesion Molecule Like | Q14982 |
| STN1 | STN1 Subunit Of CST Complex | Q9H668 |
| SPAM1 | Sperm Adhesion Molecule 1 | P38567 |
| PPP1R15A | Protein Phosphatase 1 Regulatory Subunit 15A | O75807 |
| P4HA1 | Prolyl 4-Hydroxylase Subunit Alpha 1 | P13674 |
| GPR33 | G Protein-Coupled Receptor 33 | Q49SQ1 |
| MIR4511 | MicroRNA 4511 |  |
| HOXA13 | Homeobox A13 | P31271 |
| PLS3 | Plastin 3 | P13797 |
| IFNGR2 | Interferon Gamma Receptor 2 | P38484 |
| LRBA | LPS Responsive Beige-Like Anchor Protein | P50851 |
| PKN2 | Protein Kinase N2 | Q16513 |
| E2F7 | E2F Transcription Factor 7 | Q96AV8 |
| MEGF11 | Multiple EGF Like Domains 11 | A6BM72 |
| AMBRA1 | Autophagy And Beclin 1 Regulator 1 | Q9C0C7 |
| H4C9 | H4 Clustered Histone 9 | P62805 |
| FIGN | Fidgetin, Microtubule Severing Factor | Q5HY92 |
| TYROBP | Transmembrane Immune Signaling Adaptor TYROBP | O43914 |
| CLN5 | CLN5 Intracellular Trafficking Protein | O75503 |
| ERGIC1 | Endoplasmic Reticulum-Golgi Intermediate Compartment 1 | Q969X5 |
| MAGI2 | Membrane Associated Guanylate Kinase, WW And PDZ Domain Containing 2 | Q86UL8 |
| HOXC6 | Homeobox C6 | P09630 |
| MIR124-1HG | MIR124-1 Host Gene |  |
| PDE11A | Phosphodiesterase 11A | Q9HCR9 |
| ARTN | Artemin | Q5T4W7 |
| MTPAP | Mitochondrial Poly(A) Polymerase | Q9NVV4 |
| LOC110006319 | Beta-Globin Gene 3' Regulatory Region |  |
| NRTN | Neurturin | Q99748 |
| TRB | T Cell Receptor Beta Locus | P0DSE2 |
| WIF1 | WNT Inhibitory Factor 1 | Q9Y5W5 |
| H4C11 | H4 Clustered Histone 11 | P62805 |
| WNT5B | Wnt Family Member 5B | Q9H1J7 |
| MCM10 | Minichromosome Maintenance 10 Replication Initiation Factor | Q7L590 |
| ST18 | ST18 C2H2C-Type Zinc Finger Transcription Factor | O60284 |
| TM2D3 | TM2 Domain Containing 3 | Q9BRN9 |
| NHS | NHS Actin Remodeling Regulator | Q6T4R5 |
| SLC20A1 | Solute Carrier Family 20 Member 1 | Q8WUM9 |
| CLCN6 | Chloride Voltage-Gated Channel 6 | P51797 |
| NAALAD2 | N-Acetylated Alpha-Linked Acidic Dipeptidase 2 | Q9Y3Q0 |
| MIR4293 | MicroRNA 4293 |  |
| ENSG00000255426 | Novel Transcript |  |
| BCYRN1 | Brain Cytoplasmic RNA 1 |  |
| YTHDC2 | YTH N6-Methyladenosine RNA Binding Protein C2 | Q9H6S0 |
| SMYD1 | SET And MYND Domain Containing 1 | Q8NB12 |
| SCARF1 | Scavenger Receptor Class F Member 1 | Q14162 |
| ALOX12 | Arachidonate 12-Lipoxygenase, 12S Type | P18054 |
| RBBP7 | RB Binding Protein 7, Chromatin Remodeling Factor | Q16576 |
| MYBL2 | MYB Proto-Oncogene Like 2 | P10244 |
| FAF2 | Fas Associated Factor Family Member 2 | Q96CS3 |
| VKORC1 | Vitamin K Epoxide Reductase Complex Subunit 1 | Q9BQB6 |
| DSTYK | Dual Serine/Threonine And Tyrosine Protein Kinase | Q6XUX3 |
| GNAI1 | G Protein Subunit Alpha I1 | P63096 |
| SLC7A11 | Solute Carrier Family 7 Member 11 | Q9UPY5 |
| LNCAROD | LncRNA Activating Regulator Of DKK1 |  |
| PRPSAP1 | Phosphoribosyl Pyrophosphate Synthetase Associated Protein 1 | Q14558 |
| PRDM5 | PR/SET Domain 5 | Q9NQX1 |
| ANKRD17 | Ankyrin Repeat Domain 17 | O75179 |
| DRAP1 | DR1 Associated Protein 1 | Q14919 |
| MGAM | Maltase-Glucoamylase | O43451 |
| GDF11 | Growth Differentiation Factor 11 | O95390 |
| CLN8 | CLN8 Transmembrane ER And ERGIC Protein | Q9UBY8 |
| HOXA5 | Homeobox A5 | P20719 |
| PTPRD | Protein Tyrosine Phosphatase Receptor Type D | P23468 |
| EIF2AK1 | Eukaryotic Translation Initiation Factor 2 Alpha Kinase 1 | Q9BQI3 |
| PAG1 | Phosphoprotein Membrane Anchor With Glycosphingolipid Microdomains 1 | Q9NWQ8 |
| EP300-AS1 | EP300 Antisense RNA 1 |  |
| LOC126863158 | BRD4-Independent Group 4 Enhancer GRCh37_chr22:41547383-41548582 |  |
| PCSK5 | Proprotein Convertase Subtilisin/Kexin Type 5 | Q92824 |
| MARCHF8 | Membrane Associated Ring-CH-Type Finger 8 | Q5T0T0 |
| VTCN1 | V-Set Domain Containing T Cell Activation Inhibitor 1 | Q7Z7D3 |
| SYNE2 | Spectrin Repeat Containing Nuclear Envelope Protein 2 | Q8WXH0 |
| ADAMTS20 | ADAM Metallopeptidase With Thrombospondin Type 1 Motif 20 | P59510 |
| P2RX4 | Purinergic Receptor P2X 4 | Q99571 |
| RSL24D1 | Ribosomal L24 Domain Containing 1 | Q9UHA3 |
| HOXA11 | Homeobox A11 | P31270 |
| GCK | Glucokinase | P35557 |
| LAMC3 | Laminin Subunit Gamma 3 | Q9Y6N6 |
| NPTX1 | Neuronal Pentraxin 1 | Q15818 |
| SDR9C7 | Short Chain Dehydrogenase/Reductase Family 9C Member 7 | Q8NEX9 |
| HOXA10 | Homeobox A10 | P31260 |
| KAZALD1 | Kazal Type Serine Peptidase Inhibitor Domain 1 | Q96I82 |
| VASP | Vasodilator Stimulated Phosphoprotein | P50552 |
| PAPPA-AS1 | PAPPA Antisense RNA 1 | Q5QFB9 |
| LYRM7 | LYR Motif Containing 7 | Q5U5X0 |
| GPR26 | G Protein-Coupled Receptor 26 | Q8NDV2 |
| HEMGN | Hemogen | Q9BXL5 |
| MIR378C | MicroRNA 378c |  |
| H2BC21 | H2B Clustered Histone 21 | Q16778 |
| MIR486-2 | MicroRNA 486-2 |  |
| NUP98 | Nucleoporin 98 And 96 Precursor | P52948 |
| IL17RC | Interleukin 17 Receptor C | Q8NAC3 |
| MTREX | Mtr4 Exosome RNA Helicase | P42285 |
| MCHR1 | Melanin Concentrating Hormone Receptor 1 | Q99705 |
| SPATA7 | Spermatogenesis Associated 7 | Q9P0W8 |
| PTPRJ | Protein Tyrosine Phosphatase Receptor Type J | Q12913 |
| TOB1 | Transducer Of ERBB2, 1 | P50616 |
| CARHSP1 | Calcium Regulated Heat Stable Protein 1 | Q9Y2V2 |
| CRLF3 | Cytokine Receptor Like Factor 3 | Q8IUI8 |
| FERMT3 | FERM Domain Containing Kindlin 3 | Q86UX7 |
| PGK1 | Phosphoglycerate Kinase 1 | P00558 |
| KCNC3 | Potassium Voltage-Gated Channel Subfamily C Member 3 | Q14003 |
| TRPC3 | Transient Receptor Potential Cation Channel Subfamily C Member 3 | Q13507 |
| RF03967-002 | |  |
| MKRN2 | Makorin Ring Finger Protein 2 | Q9H000 |
| CFTR-AS1 | CFTR Antisense RNA 1 |  |
| SSR1 | Signal Sequence Receptor Subunit 1 | P43307 |
| SLC12A1 | Solute Carrier Family 12 Member 1 | Q13621 |
| PCDH15 | Protocadherin Related 15 | Q96QU1 |
| PUM1 | Pumilio RNA Binding Family Member 1 | Q14671 |
| TLE5 | TLE Family Member 5, Transcriptional Modulator | Q08117 |
| PRICKLE4 | Prickle Planar Cell Polarity Protein 4 | Q2TBC4 |
| INVS | Inversin | Q9Y283 |
| MMRN1 | Multimerin 1 | Q13201 |
| XPNPEP3 | X-Prolyl Aminopeptidase 3 | Q9NQH7 |
| EXOC4 | Exocyst Complex Component 4 | Q96A65 |
| C9orf163 | Chromosome 9 Putative Open Reading Frame 163 | Q8N9P6 |
| SLC8A1 | Solute Carrier Family 8 Member A1 | P32418 |
| KLHDC2 | Kelch Domain Containing 2 | Q9Y2U9 |
| KCNQ1-AS1 | KCNQ1 Antisense RNA 1 |  |
| SLC4A4 | Solute Carrier Family 4 Member 4 | Q9Y6R1 |
| CAPZB | Capping Actin Protein Of Muscle Z-Line Subunit Beta | P47756 |
| MIR596 | MicroRNA 596 |  |
| CBX5 | Chromobox 5 | P45973 |
| FZD8 | Frizzled Class Receptor 8 | Q9H461 |
| NMD3 | NMD3 Ribosome Export Adaptor | Q96D46 |
| RPF2 | Ribosome Production Factor 2 Homolog | Q9H7B2 |
| CTBS | Chitobiase | Q01459 |
| SMOC1 | SPARC Related Modular Calcium Binding 1 | Q9H4F8 |
| CPT1A | Carnitine Palmitoyltransferase 1A | P50416 |
| VPS37A | VPS37A Subunit Of ESCRT-I | Q8NEZ2 |
| TMED2 | Transmembrane P24 Trafficking Protein 2 | Q15363 |
| MED30 | Mediator Complex Subunit 30 | Q96HR3 |
| SNU13 | Small Nuclear Ribonucleoprotein 13 | P55769 |
| TLE1 | TLE Family Member 1, Transcriptional Corepressor | Q04724 |
| DLG2 | Discs Large MAGUK Scaffold Protein 2 | Q15700 |
| PIK3C3 | Phosphatidylinositol 3-Kinase Catalytic Subunit Type 3 | Q8NEB9 |
| SF1 | Splicing Factor 1 | Q15637 |
| MIR495 | MicroRNA 495 |  |
| RASIP1 | Ras Interacting Protein 1 | Q5U651 |
| CCDC6 | Coiled-Coil Domain Containing 6 | Q16204 |
| S100A11 | S100 Calcium Binding Protein A11 | P31949 |
| MIR4487 | MicroRNA 4487 |  |
| PAM | Peptidylglycine Alpha-Amidating Monooxygenase | P19021 |
| CEACAM1 | CEA Cell Adhesion Molecule 1 | P13688 |
| MED22 | Mediator Complex Subunit 22 | Q15528 |
| ESRRA | Estrogen Related Receptor Alpha | P11474 |
| COPS3 | COP9 Signalosome Subunit 3 | Q9UNS2 |
| MIR4284 | MicroRNA 4284 |  |
| SRPRA | SRP Receptor Subunit Alpha | P08240 |
| OGDH | Oxoglutarate Dehydrogenase | Q02218 |
| PODXL | Podocalyxin Like | O00592 |
| PER2 | Period Circadian Regulator 2 | O15055 |
| LNPK | Lunapark, ER Junction Formation Factor | Q9C0E8 |
| AEBP1 | AE Binding Protein 1 | Q8IUX7 |
| NTAN1 | N-Terminal Asparagine Amidase | Q96AB6 |
| LINC02870 | Long Intergenic Non-Protein Coding RNA 2870 |  |
| LINC00494 | Long Intergenic Non-Protein Coding RNA 494 |  |
| MIR3137 | MicroRNA 3137 |  |
| UBE2O | Ubiquitin Conjugating Enzyme E2 O | Q9C0C9 |
| FGR | FGR Proto-Oncogene, Src Family Tyrosine Kinase | P09769 |
| ANK2 | Ankyrin 2 | Q01484 |
| P3H2 | Prolyl 3-Hydroxylase 2 | Q8IVL5 |
| FGF19 | Fibroblast Growth Factor 19 | O95750 |
| ATP6V1E1 | ATPase H+ Transporting V1 Subunit E1 | P36543 |
| CELF1 | CUGBP Elav-Like Family Member 1 | Q92879 |
| IL1RAPL1 | Interleukin 1 Receptor Accessory Protein Like 1 | Q9NZN1 |
| P2RX1 | Purinergic Receptor P2X 1 | P51575 |
| UGT8 | UDP Glycosyltransferase 8 | Q16880 |
| DERL1 | Derlin 1 | Q9BUN8 |
| LRRC7 | Leucine Rich Repeat Containing 7 | Q96NW7 |
| MRPL28 | Mitochondrial Ribosomal Protein L28 | Q13084 |
| PANX1 | Pannexin 1 | Q96RD7 |
| H2AZ2 | H2A.Z Variant Histone 2 | Q71UI9 |
| MIR516B1 | MicroRNA 516b-1 |  |
| MIR516B2 | MicroRNA 516b-2 |  |
| DIS3 | DIS3 Homolog, Exosome Endoribonuclease And 3'-5' Exoribonuclease | Q9Y2L1 |
| SELPLG | Selectin P Ligand | Q14242 |
| SNORA75 | Small Nucleolar RNA, H/ACA Box 75 |  |
| WNT7B | Wnt Family Member 7B | P56706 |
| TFG | Trafficking From ER To Golgi Regulator | Q92734 |
| CCR8 | C-C Motif Chemokine Receptor 8 | P51685 |
| MYO1F | Myosin IF | O00160 |
| HOXD9 | Homeobox D9 | P28356 |
| RBBP4 | RB Binding Protein 4, Chromatin Remodeling Factor | Q09028 |
| B4GALT6 | Beta-1,4-Galactosyltransferase 6 | Q9UBX8 |
| SPRY2 | Sprouty RTK Signaling Antagonist 2 | O43597 |
| HTR2B | 5-Hydroxytryptamine Receptor 2B | P41595 |
| NAV2-AS1 | NAV2 Antisense RNA 1 |  |
| SETDB1 | SET Domain Bifurcated Histone Lysine Methyltransferase 1 | Q15047 |
| EIF4B | Eukaryotic Translation Initiation Factor 4B | P23588 |
| MTHFSD | Methenyltetrahydrofolate Synthetase Domain Containing | Q2M296 |
| ESM1 | Endothelial Cell Specific Molecule 1 | Q9NQ30 |
| S1PR1 | Sphingosine-1-Phosphate Receptor 1 | P21453 |
| ATP5MK | ATP Synthase Membrane Subunit K | Q96IX5 |
| CITED2 | Cbp/P300 Interacting Transactivator With Glu/Asp Rich Carboxy-Terminal Domain 2 | Q99967 |
| H4C1 | H4 Clustered Histone 1 | P62805 |
| PDE10A | Phosphodiesterase 10A | Q9Y233 |
| FERMT2 | FERM Domain Containing Kindlin 2 | Q96AC1 |
| SNRPA | Small Nuclear Ribonucleoprotein Polypeptide A | P09012 |
| VASH1 | Vasohibin 1 | Q7L8A9 |
| SEC24B | SEC24 Homolog B, COPII Coat Complex Component | O95487 |
| MR1 | Major Histocompatibility Complex, Class I-Related | Q95460 |
| VGLL4 | Vestigial Like Family Member 4 | Q14135 |
| NPAS3 | Neuronal PAS Domain Protein 3 | Q8IXF0 |
| VIL1 | Villin 1 | P09327 |
| AMN | Amnion Associated Transmembrane Protein | Q9BXJ7 |
| RASGRP1 | RAS Guanyl Releasing Protein 1 | O95267 |
| PCDH20 | Protocadherin 20 | Q8N6Y1 |
| RAB6A | RAB6A, Member RAS Oncogene Family | P20340 |
| CBLB | Cbl Proto-Oncogene B | Q13191 |
| RASAL3 | RAS Protein Activator Like 3 | Q86YV0 |
| PPP1R27 | Protein Phosphatase 1 Regulatory Subunit 27 | Q86WC6 |
| COL14A1 | Collagen Type XIV Alpha 1 Chain | Q05707 |
| ZYX | Zyxin | Q15942 |
| NFIC | Nuclear Factor I C | P08651 |
| LYVE1 | Lymphatic Vessel Endothelial Hyaluronan Receptor 1 | Q9Y5Y7 |
| CTDP1 | CTD Phosphatase Subunit 1 | Q9Y5B0 |
| GORASP2 | Golgi Reassembly Stacking Protein 2 | Q9H8Y8 |
| COX17 | Cytochrome C Oxidase Copper Chaperone COX17 | Q14061 |
| LINC02154 | Long Intergenic Non-Protein Coding RNA 2154 |  |
| H2AZ1 | H2A.Z Variant Histone 1 | P0C0S5 |
| MIR22HG | MIR22 Host Gene | Q0VDD5 |
| LINC01550 | Long Intergenic Non-Protein Coding RNA 1550 |  |
| MIR7-3 | MicroRNA 7-3 |  |
| MED11 | Mediator Complex Subunit 11 | Q9P086 |
| SLC2A11 | Solute Carrier Family 2 Member 11 | Q9BYW1 |
| HSD17B1 | Hydroxysteroid 17-Beta Dehydrogenase 1 | P14061 |
| FUCA2 | Alpha-L-Fucosidase 2 | Q9BTY2 |
| CLTCL1 | Clathrin Heavy Chain Like 1 | P53675 |
| SIAH2 | Siah E3 Ubiquitin Protein Ligase 2 | O43255 |
| FSTL1 | Follistatin Like 1 | Q12841 |
| JAM2 | Junctional Adhesion Molecule 2 | P57087 |
| HBZ | Hemoglobin Subunit Zeta | P02008 |
| SMAD5 | SMAD Family Member 5 | Q99717 |
| RNPS1 | RNA Binding Protein With Serine Rich Domain 1 | Q15287 |
| ABCF2 | ATP Binding Cassette Subfamily F Member 2 | Q9UG63 |
| SMOC2 | SPARC Related Modular Calcium Binding 2 | Q9H3U7 |
| HSPE1P1 | Heat Shock Protein Family E (Hsp10) Member 1 Pseudogene 1 |  |
| STK17A | Serine/Threonine Kinase 17a | Q9UEE5 |
| H4C15 | H4 Clustered Histone 15 | P62805 |
| RIMS1 | Regulating Synaptic Membrane Exocytosis 1 | Q86UR5 |
| RPS4X | Ribosomal Protein S4 X-Linked | P62701 |
| MIR7-2 | MicroRNA 7-2 |  |
| DMGDH | Dimethylglycine Dehydrogenase | Q9UI17 |
| NCOA6 | Nuclear Receptor Coactivator 6 | Q14686 |
| IL12RB2 | Interleukin 12 Receptor Subunit Beta 2 | Q99665 |
| ZNHIT1 | Zinc Finger HIT-Type Containing 1 | O43257 |
| PHKA2 | Phosphorylase Kinase Regulatory Subunit Alpha 2 | P46019 |
| MAP1LC3A | Microtubule Associated Protein 1 Light Chain 3 Alpha | Q9H492 |
| CARM1 | Coactivator Associated Arginine Methyltransferase 1 | Q86X55 |
| CKAP5 | Cytoskeleton Associated Protein 5 | Q14008 |
| TIAM1 | TIAM Rac1 Associated GEF 1 | Q13009 |
| FAN1 | FANCD2 And FANCI Associated Nuclease 1 | Q9Y2M0 |
| PITRM1 | Pitrilysin Metallopeptidase 1 | Q5JRX3 |
| FGD4 | FYVE, RhoGEF And PH Domain Containing 4 | Q96M96 |
| BCAR4 | Breast Cancer Anti-Estrogen Resistance 4 |  |
| TCL1A | TCL1 Family AKT Coactivator A | P56279 |
| CCN3 | Cellular Communication Network Factor 3 | P48745 |
| GPR89B | G Protein-Coupled Receptor 89B | P0CG08 |
| SCIN | Scinderin | Q9Y6U3 |
| ORC5 | Origin Recognition Complex Subunit 5 | O43913 |
| TLN2 | Talin 2 | Q9Y4G6 |
| CALB1 | Calbindin 1 | P05937 |
| DNAJC5 | DnaJ Heat Shock Protein Family (Hsp40) Member C5 | Q9H3Z4 |
| CHST15 | Carbohydrate Sulfotransferase 15 | Q7LFX5 |
| NFAT5 | Nuclear Factor Of Activated T Cells 5 | O94916 |
| NIP7 | Nucleolar Pre-RRNA Processing Protein NIP7 | Q9Y221 |
| LSG1 | Large 60S Subunit Nuclear Export GTPase 1 | Q9H089 |
| MRTO4 | MRT4 Homolog, Ribosome Maturation Factor | Q9UKD2 |
| HSD17B2 | Hydroxysteroid 17-Beta Dehydrogenase 2 | P37059 |
| LEF1-AS1 | LEF1 Antisense RNA 1 |  |
| TMEM263 | Transmembrane Protein 263 | Q8WUH6 |
| TET1 | Tet Methylcytosine Dioxygenase 1 | Q8NFU7 |
| TSPAN2 | Tetraspanin 2 | O60636 |
| CBLN2 | Cerebellin 2 Precursor | Q8IUK8 |
| DRD4 | Dopamine Receptor D4 | P21917 |
| LINGO2 | Leucine Rich Repeat And Ig Domain Containing 2 | Q7L985 |
| RFX6 | Regulatory Factor X6 | Q8HWS3 |
| RGS8 | Regulator Of G Protein Signaling 8 | P57771 |
| INO80 | INO80 Complex ATPase Subunit | Q9ULG1 |
| SLC31A1 | Solute Carrier Family 31 Member 1 | O15431 |
| LINC01152 | Long Intergenic Non-Protein Coding RNA 1152 |  |
| ZNF326 | Zinc Finger Protein 326 | Q5BKZ1 |
| PDZK1 | PDZ Domain Containing 1 | Q5T2W1 |
| LZTS1 | Leucine Zipper Tumor Suppressor 1 | Q9Y250 |
| CLEC2D | C-Type Lectin Domain Family 2 Member D | Q9UHP7 |
| HOXB9 | Homeobox B9 | P17482 |
| ROCR | Regulator Of Chondrogenesis RNA |  |
| AMPD3 | Adenosine Monophosphate Deaminase 3 | Q01432 |
| PHF5A | PHD Finger Protein 5A | Q7RTV0 |
| TRIML2 | Tripartite Motif Family Like 2 | Q8N7C3 |
| KCNJ16 | Potassium Inwardly Rectifying Channel Subfamily J Member 16 | Q9NPI9 |
| IL18RAP | Interleukin 18 Receptor Accessory Protein | O95256 |
| CD248 | CD248 Molecule | Q9HCU0 |
| BRINP3 | BMP/Retinoic Acid Inducible Neural Specific 3 | Q76B58 |
| MIR133A2 | MicroRNA 133a-2 |  |
| RND3 | Rho Family GTPase 3 | P61587 |
| GZMM | Granzyme M | P51124 |
| CETN3 | Centrin 3 | O15182 |
| ARMC5 | Armadillo Repeat Containing 5 | Q96C12 |
| EIF5 | Eukaryotic Translation Initiation Factor 5 | P55010 |
| GNA15 | G Protein Subunit Alpha 15 | P30679 |
| SERPINA5 | Serpin Family A Member 5 | P05154 |
| CAMK4 | Calcium/Calmodulin Dependent Protein Kinase IV | Q16566 |
| AMH | Anti-Mullerian Hormone | P03971 |
| BEAN1 | Brain Expressed Associated With NEDD4 1 | Q3B7T3 |
| TMEM11 | Transmembrane Protein 11 | P17152 |
| ATOH1 | Atonal BHLH Transcription Factor 1 | Q92858 |
| NME8 | NME/NM23 Family Member 8 | Q8N427 |
| PDE3A | Phosphodiesterase 3A | Q14432 |
| HDAC7 | Histone Deacetylase 7 | Q8WUI4 |
| EID3 | EP300 Interacting Inhibitor Of Differentiation 3 | Q8N140 |
| HOXA@ | Homeobox A Cluster |  |
| AJAP1 | Adherens Junctions Associated Protein 1 | Q9UKB5 |
| MARCKS | Myristoylated Alanine Rich Protein Kinase C Substrate | P29966 |
| SLC37A2 | Solute Carrier Family 37 Member 2 | Q8TED4 |
| TLDC2 | TBC/LysM-Associated Domain Containing 2 | A0PJX2 |
| SCG2 | Secretogranin II | P13521 |
| CYP3A7 | Cytochrome P450 Family 3 Subfamily A Member 7 | P24462 |
| ZCCHC8 | Zinc Finger CCHC-Type Containing 8 | Q6NZY4 |
| STPG2 | Sperm Tail PG-Rich Repeat Containing 2 | Q8N412 |
| TMEM100 | Transmembrane Protein 100 | Q9NV29 |
| MIR138-2 | MicroRNA 138-2 |  |
| MIR576 | MicroRNA 576 |  |
| MIR551A | MicroRNA 551a |  |
| H4C8 | H4 Clustered Histone 8 | P62805 |
| EHHADH | Enoyl-CoA Hydratase And 3-Hydroxyacyl CoA Dehydrogenase | Q08426 |
| PXT1 | Peroxisomal Testis Enriched Protein 1 | Q8NFP0 |
| EVA1A | Eva-1 Homolog A, Regulator Of Programmed Cell Death | Q9H8M9 |
| NTM | Neurotrimin | Q9P121 |
| SNORA66 | Small Nucleolar RNA, H/ACA Box 66 |  |
| PTBP2 | Polypyrimidine Tract Binding Protein 2 | Q9UKA9 |
| PFDN5 | Prefoldin Subunit 5 | Q99471 |
| PRLR | Prolactin Receptor | P16471 |
| CAND1 | Cullin Associated And Neddylation Dissociated 1 | Q86VP6 |
| RAB3A | RAB3A, Member RAS Oncogene Family | P20336 |
| FAP | Fibroblast Activation Protein Alpha | Q12884 |
| TNN | Tenascin N | Q9UQP3 |
| SCAP | SREBF Chaperone | Q12770 |
| BRMS1 | BRMS1 Transcriptional Repressor And Anoikis Regulator | Q9HCU9 |
| NR4A3 | Nuclear Receptor Subfamily 4 Group A Member 3 | Q92570 |
| MAN2B2 | Mannosidase Alpha Class 2B Member 2 | Q9Y2E5 |
| MAP3K12 | Mitogen-Activated Protein Kinase Kinase Kinase 12 | Q12852 |
| CNTN5 | Contactin 5 | O94779 |
| RBM7 | RNA Binding Motif Protein 7 | Q9Y580 |
| ATG14 | Autophagy Related 14 | Q6ZNE5 |
| IGSF3 | Immunoglobulin Superfamily Member 3 | O75054 |
| MACROD2 | Mono-ADP Ribosylhydrolase 2 | A1Z1Q3 |
| FEZ2 | Fasciculation And Elongation Protein Zeta 2 | Q9UHY8 |
| TMEM182 | Transmembrane Protein 182 | Q6ZP80 |
| SEMA3C | Semaphorin 3C | Q99985 |
| ADRA1B | Adrenoceptor Alpha 1B | P35368 |
| EIF3H | Eukaryotic Translation Initiation Factor 3 Subunit H | O15372 |
| ZHX1 | Zinc Fingers And Homeoboxes 1 | Q9UKY1 |
| UTP23 | UTP23 Small Subunit Processome Component | Q9BRU9 |
| PKDREJ | Polycystin Family Receptor For Egg Jelly | Q9NTG1 |
| OR4D6 | Olfactory Receptor Family 4 Subfamily D Member 6 | Q8NGJ1 |
| GASK1A | Golgi Associated Kinase 1A | Q9UFP1 |
| ZNF713 | Zinc Finger Protein 713 | Q8N859 |
| AARD | Alanine And Arginine Rich Domain Containing Protein | Q4LEZ3 |
| RAD21-AS1 | RAD21 Antisense RNA 1 |  |
| MIR3610 | MicroRNA 3610 |  |
| PRICKLE1 | Prickle Planar Cell Polarity Protein 1 | Q96MT3 |
| DUSP5 | Dual Specificity Phosphatase 5 | Q16690 |
| DLC1 | DLC1 Rho GTPase Activating Protein | Q96QB1 |
| DNPEP | Aspartyl Aminopeptidase | Q9ULA0 |
| H4C4 | H4 Clustered Histone 4 | P62805 |
| H4C6 | H4 Clustered Histone 6 | P62805 |
| DAZ1 | Deleted In Azoospermia 1 | Q9NQZ3 |
| DECR1 | 2,4-Dienoyl-CoA Reductase 1 | Q16698 |
| TLL1 | Tolloid Like 1 | O43897 |
| SRD5A1 | Steroid 5 Alpha-Reductase 1 | P18405 |
| FKBP4 | FKBP Prolyl Isomerase 4 | Q02790 |
| SOCS2 | Suppressor Of Cytokine Signaling 2 | O14508 |
| TTC7B | Tetratricopeptide Repeat Domain 7B | Q86TV6 |
| MSRA | Methionine Sulfoxide Reductase A | Q9UJ68 |
| KIAA1549 | KIAA1549 | Q9HCM3 |
| PER3 | Period Circadian Regulator 3 | P56645 |
| MCM6 | Minichromosome Maintenance Complex Component 6 | Q14566 |
| NAALADL2 | N-Acetylated Alpha-Linked Acidic Dipeptidase Like 2 | Q58DX5 |
| MELTF | Melanotransferrin | P08582 |
| URB2 | URB2 Ribosome Biogenesis Homolog | Q14146 |
| LOC123497917 | Sharpr-MPRA Regulatory Region 14877 |  |
| PTPN1 | Protein Tyrosine Phosphatase Non-Receptor Type 1 | P18031 |
| SFMBT2 | Scm Like With Four Mbt Domains 2 | Q5VUG0 |
| PTPN9 | Protein Tyrosine Phosphatase Non-Receptor Type 9 | P43378 |
| PDLIM7 | PDZ And LIM Domain 7 | Q9NR12 |
| COL20A1 | Collagen Type XX Alpha 1 Chain | Q9P218 |
| H4C12 | H4 Clustered Histone 12 | P62805 |
| H4C2 | H4 Clustered Histone 2 | P62805 |
| H4C13 | H4 Clustered Histone 13 | P62805 |
| H4C14 | H4 Clustered Histone 14 | P62805 |
| GLRX5 | Glutaredoxin 5 | Q86SX6 |
| ARSG | Arylsulfatase G | Q96EG1 |
| ORC3 | Origin Recognition Complex Subunit 3 | Q9UBD5 |
| SPMIP6 | Sperm Microtubule Inner Protein 6 | Q8NCR6 |
| IFNLR1 | Interferon Lambda Receptor 1 | Q8IU57 |
| COQ5 | Coenzyme Q5, Methyltransferase | Q5HYK3 |
| MIR1306 | MicroRNA 1306 |  |
| ADRA2B | Adrenoceptor Alpha 2B | P18089 |
| TNKS | Tankyrase | O95271 |
| NAB1 | NGFI-A Binding Protein 1 | Q13506 |
| PREX1 | Phosphatidylinositol-3,4,5-Trisphosphate Dependent Rac Exchange Factor 1 | Q8TCU6 |
| EXOSC7 | Exosome Component 7 | Q15024 |
| EXOSC4 | Exosome Component 4 | Q9NPD3 |
| EXOSC6 | Exosome Component 6 | Q5RKV6 |
| DIS3L | DIS3 Like Exosome 3'-5' Exoribonuclease | Q8TF46 |
| ALDH1L1 | Aldehyde Dehydrogenase 1 Family Member L1 | O75891 |
| UHRF1 | Ubiquitin Like With PHD And Ring Finger Domains 1 | Q96T88 |
| FKBP5 | FKBP Prolyl Isomerase 5 | Q13451 |
| PCBP2 | Poly(RC) Binding Protein 2 | Q15366 |
| SLC34A2 | Solute Carrier Family 34 Member 2 | O95436 |
| YPEL1 | Yippee Like 1 | O60688 |
| EIF2AK4 | Eukaryotic Translation Initiation Factor 2 Alpha Kinase 4 | Q9P2K8 |
| SWT1 | SWT1 RNA Endoribonuclease Homolog | Q5T5J6 |
| SPON1 | Spondin 1 | Q9HCB6 |
| HOXB4 | Homeobox B4 | P17483 |
| ZNF346 | Zinc Finger Protein 346 | Q9UL40 |
| UTS2 | Urotensin 2 | O95399 |
| CLEC16A | C-Type Lectin Domain Containing 16A | Q2KHT3 |
| GSTA2 | Glutathione S-Transferase Alpha 2 | P09210 |
| POLE2 | DNA Polymerase Epsilon 2, Accessory Subunit | P56282 |
| MIR329-1 | MicroRNA 329-1 |  |
| TGOLN2 | Trans-Golgi Network Protein 2 | O43493 |
| SLC25A38 | Solute Carrier Family 25 Member 38 | Q96DW6 |
| RAP1GDS1 | Rap1 GTPase-GDP Dissociation Stimulator 1 | P52306 |
| SLC12A3 | Solute Carrier Family 12 Member 3 | P55017 |
| GPC5 | Glypican 5 | P78333 |
| DNAJC3 | DnaJ Heat Shock Protein Family (Hsp40) Member C3 | Q13217 |
| OSBPL9 | Oxysterol Binding Protein Like 9 | Q96SU4 |
| DTNB | Dystrobrevin Beta | O60941 |
| MIR3175 | MicroRNA 3175 |  |
| CYP4F2 | Cytochrome P450 Family 4 Subfamily F Member 2 | P78329 |
| TMCO4 | Transmembrane And Coiled-Coil Domains 4 | Q5TGY1 |
| EHBP1L1 | EH Domain Binding Protein 1 Like 1 | Q8N3D4 |
| CLTA | Clathrin Light Chain A | P09496 |
| GNA13 | G Protein Subunit Alpha 13 | Q14344 |
| PPP1R14A | Protein Phosphatase 1 Regulatory Inhibitor Subunit 14A | Q96A00 |
| PNPLA8 | Patatin Like Phospholipase Domain Containing 8 | Q9NP80 |
| PLN | Phospholamban | P26678 |
| RPH3A | Rabphilin 3A | Q9Y2J0 |
| B3GALT4 | Beta-1,3-Galactosyltransferase 4 | O96024 |
| ZHX3 | Zinc Fingers And Homeoboxes 3 | Q9H4I2 |
| MAML3 | Mastermind Like Transcriptional Coactivator 3 | Q96JK9 |
| ATG13 | Autophagy Related 13 | O75143 |
| DGKB | Diacylglycerol Kinase Beta | Q9Y6T7 |
| PARVA | Parvin Alpha | Q9NVD7 |
| GOLGA1 | Golgin A1 | Q92805 |
| CCDC33 | Coiled-Coil Domain Containing 33 | Q8N5R6 |
| SEMA7A | Semaphorin 7A (John Milton Hagen Blood Group) | O75326 |
| ADPRH | ADP-Ribosylarginine Hydrolase | P54922 |
| CMSS1 | Cms1 Ribosomal Small Subunit Homolog | Q9BQ75 |
| RMST | Rhabdomyosarcoma 2 Associated Transcript |  |
| SNCAIP | Synuclein Alpha Interacting Protein | Q9Y6H5 |
| EIF3B | Eukaryotic Translation Initiation Factor 3 Subunit B | P55884 |
| CALM3 | Calmodulin 3 | P0DP25 |
| PARD3 | Par-3 Family Cell Polarity Regulator | Q8TEW0 |
| TUT4 | Terminal Uridylyl Transferase 4 | Q5TAX3 |
| APPL1 | Adaptor Protein, Phosphotyrosine Interacting With PH Domain And Leucine Zipper 1 | Q9UKG1 |
| CBX3 | Chromobox 3 | Q13185 |
| ARHGEF18 | Rho/Rac Guanine Nucleotide Exchange Factor 18 | Q6ZSZ5 |
| CCAT2 | Colon Cancer Associated Transcript 2 |  |
| L3MBTL3 | L3MBTL Histone Methyl-Lysine Binding Protein 3 | Q96JM7 |
| CCNB2 | Cyclin B2 | O95067 |
| GLYAT | Glycine-N-Acyltransferase | Q6IB77 |
| MARCO | Macrophage Receptor With Collagenous Structure | Q9UEW3 |
| LPXN | Leupaxin | O60711 |
| ATRAID | All-Trans Retinoic Acid Induced Differentiation Factor | Q6UW56 |
| CDC5L | Cell Division Cycle 5 Like | Q99459 |
| HECTD4 | HECT Domain E3 Ubiquitin Protein Ligase 4 | Q9Y4D8 |
| LOC110973015 | NOS3 5' Regulatory Region |  |
| UBXN4 | UBX Domain Protein 4 | Q92575 |
| ROBO2 | Roundabout Guidance Receptor 2 | Q9HCK4 |
| SAR1B | Secretion Associated Ras Related GTPase 1B | Q9Y6B6 |
| ST2 | Suppression Of Tumorigenicity 2 |  |
| SLC24A3 | Solute Carrier Family 24 Member 3 | Q9HC58 |
| H1-3 | H1.3 Linker Histone, Cluster Member | P16402 |
| KMT5A | Lysine Methyltransferase 5A | Q9NQR1 |
| DBF4 | DBF4 Zinc Finger | Q9UBU7 |
| TICRR | TOPBP1 Interacting Checkpoint And Replication Regulator | Q7Z2Z1 |
| ZNF621 | Zinc Finger Protein 621 | Q6ZSS3 |
| LGR4 | Leucine Rich Repeat Containing G Protein-Coupled Receptor 4 | Q9BXB1 |
| FAM234A | Family With Sequence Similarity 234 Member A | Q9H0X4 |
| RABGAP1 | RAB GTPase Activating Protein 1 | Q9Y3P9 |
| CDC16 | Cell Division Cycle 16 | Q13042 |
| NCOA7 | Nuclear Receptor Coactivator 7 | Q8NI08 |
| PDIA2 | Protein Disulfide Isomerase Family A Member 2 | Q13087 |
| MRPL23 | Mitochondrial Ribosomal Protein L23 | Q16540 |
| DDX1 | DEAD-Box Helicase 1 | Q92499 |
| TRIM14 | Tripartite Motif Containing 14 | Q14142 |
| HOXC8 | Homeobox C8 | P31273 |
| CRBN | Cereblon | Q96SW2 |
| HIF1A-AS2 | HIF1A Antisense RNA 2 |  |
| SART1 | Spliceosome Associated Factor 1, Recruiter Of U4/U6.U5 Tri-SnRNP | O43290 |
| RXFP2 | Relaxin Family Peptide Receptor 2 | Q8WXD0 |
| CPEB2 | Cytoplasmic Polyadenylation Element Binding Protein 2 | Q7Z5Q1 |
| PDLIM1 | PDZ And LIM Domain 1 | O00151 |
| OSBPL2 | Oxysterol Binding Protein Like 2 | Q9H1P3 |
| ARHGEF16 | Rho Guanine Nucleotide Exchange Factor 16 | Q5VV41 |
| ARID4A | AT-Rich Interaction Domain 4A | P29374 |
| LGMN | Legumain | Q99538 |
| MYLK2 | Myosin Light Chain Kinase 2 | Q9H1R3 |
| PSD3 | Pleckstrin And Sec7 Domain Containing 3 | Q9NYI0 |
| GULP1 | GULP PTB Domain Containing Engulfment Adaptor 1 | Q9UBP9 |
| NME4 | NME/NM23 Nucleoside Diphosphate Kinase 4 | O00746 |
| CA12 | Carbonic Anhydrase 12 | O43570 |
| NFYC | Nuclear Transcription Factor Y Subunit Gamma | Q13952 |
| EPB41L3 | Erythrocyte Membrane Protein Band 4.1 Like 3 | Q9Y2J2 |
| PTGDR2 | Prostaglandin D2 Receptor 2 | Q9Y5Y4 |
| EFNA5 | Ephrin A5 | P52803 |
| RNASE2 | Ribonuclease A Family Member 2 | P10153 |
| CRYBB3 | Crystallin Beta B3 | P26998 |
| PRODH | Proline Dehydrogenase 1 | O43272 |
| PITPNM3 | PITPNM Family Member 3 | Q9BZ71 |
| LIN28A | Lin-28 Homolog A | Q9H9Z2 |
| STK32B | Serine/Threonine Kinase 32B | Q9NY57 |
| ASPN | Asporin | Q9BXN1 |
| LINC00963 | Long Intergenic Non-Protein Coding RNA 963 |  |
| ZCCHC7 | Zinc Finger CCHC-Type Containing 7 | Q8N3Z6 |
| GTF3C3 | General Transcription Factor IIIC Subunit 3 | Q9Y5Q9 |
| SPP2 | Secreted Phosphoprotein 2 | Q13103 |
| GBA3 | Glucosylceramidase Beta 3 (Gene/Pseudogene) | Q9H227 |
| CRACD | Capping Protein Inhibiting Regulator Of Actin Dynamics | Q6ZU35 |
| APOO | Apolipoprotein O | Q9BUR5 |
| CACNA2D3 | Calcium Voltage-Gated Channel Auxiliary Subunit Alpha2delta 3 | Q8IZS8 |
| SERBP1 | SERPINE1 MRNA Binding Protein 1 | Q8NC51 |
| UBP1 | Upstream Binding Protein 1 | Q9NZI7 |
| FAM215A | Family With Sequence Similarity 215 Member A | Q9Y5M1 |
| CCL14 | C-C Motif Chemokine Ligand 14 | Q16627 |
| SETD7 | SET Domain Containing 7, Histone Lysine Methyltransferase | Q8WTS6 |
| MMP24 | Matrix Metallopeptidase 24 | Q9Y5R2 |
| ABI3BP | ABI Family Member 3 Binding Protein | Q7Z7G0 |
| TNR | Tenascin R | Q92752 |
| TBC1D8 | TBC1 Domain Family Member 8 | O95759 |
| RNF4 | Ring Finger Protein 4 | P78317 |
| PPP4R4 | Protein Phosphatase 4 Regulatory Subunit 4 | Q6NUP7 |
| INTS7 | Integrator Complex Subunit 7 | Q9NVH2 |
| KPNA1 | Karyopherin Subunit Alpha 1 | P52294 |
| C5orf64 | Chromosome 5 Putative Open Reading Frame 64 | Q2M2E5 |
| MIR579 | MicroRNA 579 |  |
| APIP | APAF1 Interacting Protein | Q96GX9 |
| TRPC5 | Transient Receptor Potential Cation Channel Subfamily C Member 5 | Q9UL62 |
| PFDN4 | Prefoldin Subunit 4 | Q9NQP4 |
| SNORD24 | Small Nucleolar RNA, C/D Box 24 |  |
| ANKRD36B | Ankyrin Repeat Domain 36B | Q8N2N9 |
| RIOX1 | Ribosomal Oxygenase 1 | Q9H6W3 |
| SPOCK3 | SPARC (Osteonectin), Cwcv And Kazal Like Domains Proteoglycan 3 | Q9BQ16 |
| UBAP2L | Ubiquitin Associated Protein 2 Like | Q14157 |
| MIR217 | MicroRNA 217 |  |
| JMY | Junction Mediating And Regulatory Protein, P53 Cofactor | Q8N9B5 |
| NPY1R | Neuropeptide Y Receptor Y1 | P25929 |
| PLBD2 | Phospholipase B Domain Containing 2 | Q8NHP8 |
| MIR329-2 | MicroRNA 329-2 |  |
| KHDRBS3 | KH RNA Binding Domain Containing, Signal Transduction Associated 3 | O75525 |
| TLE3 | TLE Family Member 3, Transcriptional Corepressor | Q04726 |
| ID4 | Inhibitor Of DNA Binding 4 | P47928 |
| MCF2 | MCF.2 Cell Line Derived Transforming Sequence | P10911 |
| MDGA2 | MAM Domain Containing Glycosylphosphatidylinositol Anchor 2 | Q7Z553 |
| MMP28 | Matrix Metallopeptidase 28 | Q9H239 |
| LINC00339 | Long Intergenic Non-Protein Coding RNA 339 |  |
| MIR99AHG | Mir-99a-Let-7c Cluster Host Gene |  |
| MIR4523 | MicroRNA 4523 |  |
| NAP1L4 | Nucleosome Assembly Protein 1 Like 4 | Q99733 |
| DPP10 | Dipeptidyl Peptidase Like 10 | Q8N608 |
| SLC10A2 | Solute Carrier Family 10 Member 2 | Q12908 |
| SLC16A7 | Solute Carrier Family 16 Member 7 | O60669 |
| CREB5 | CAMP Responsive Element Binding Protein 5 | Q02930 |
| PWWP2A | PWWP Domain Containing 2A | Q96N64 |
| INTS3 | Integrator Complex Subunit 3 | Q68E01 |
| ANKRD44 | Ankyrin Repeat Domain 44 | Q8N8A2 |
| SMC2 | Structural Maintenance Of Chromosomes 2 | O95347 |
| TMTC2 | Transmembrane O-Mannosyltransferase Targeting Cadherins 2 | Q8N394 |
| GYS1 | Glycogen Synthase 1 | P13807 |
| GNA12 | G Protein Subunit Alpha 12 | Q03113 |
| CBLN1 | Cerebellin 1 Precursor | P23435 |
| SCG5 | Secretogranin V | P05408 |
| GREB1 | Growth Regulating Estrogen Receptor Binding 1 | Q4ZG55 |
| DNM3OS | DNM3 Opposite Strand/Antisense RNA |  |
| KAZN | Kazrin, Periplakin Interacting Protein | Q674X7 |
| PTPRG | Protein Tyrosine Phosphatase Receptor Type G | P23470 |
| IL36A | Interleukin 36 Alpha | Q9UHA7 |
| QKI | QKI, KH Domain Containing RNA Binding | Q96PU8 |
| MCUR1 | Mitochondrial Calcium Uniporter Regulator 1 | Q96AQ8 |
| GCFC2 | GC-Rich Sequence DNA-Binding Factor 2 | P16383 |
| TRIB2 | Tribbles Pseudokinase 2 | Q92519 |
| PTPRM | Protein Tyrosine Phosphatase Receptor Type M | P28827 |
| ENSG00000285959 | Novel Transcript |  |
| TSBP1 | Testis Expressed Basic Protein 1 | Q5SRN2 |
| TANC1 | Tetratricopeptide Repeat, Ankyrin Repeat And Coiled-Coil Containing 1 | Q9C0D5 |
| INSL3 | Insulin Like 3 | P51460 |
| PPM1E | Protein Phosphatase, Mg2+/Mn2+ Dependent 1E | Q8WY54 |
| TPD52 | Tumor Protein D52 | P55327 |
| MIR760 | MicroRNA 760 |  |
| MIR1469 | MicroRNA 1469 |  |
